# Boreal chorus frog (*Pseudacris maculata*) spring calling activity, habitat metrics, and associated environmental data

**DOI:** 10.1016/j.dib.2020.106581

**Published:** 2020-11-24

**Authors:** Emma M. Brinley Buckley, Benjamin L. Gottesman, Andrew J. Caven, Mary J. Harner, Bryan C. Pijanowski

**Affiliations:** a Department of Communication, University of Nebraska at Kearney, Kearney, NE 68849, United States; b Center for Global Soundscapes and Department of Forestry & Natural Resources, Purdue University, West Lafayette, IN 47907, United States; c Platte River Whooping Crane Maintenance Trust, Wood River, NE 68883, United States; d Department of Biology, University of Nebraska at Kearney, Kearney, NE 68849, United States

**Keywords:** *Pseudacris maculata*, Boreal chorus frog, Acoustic monitoring, Multimodal, Wetlands, Central platte river, Wet meadows, Sloughs, Image-analysis, Phenology

## Abstract

Data were collected using multimodal monitoring technologies pairing sound recorders with time-lapse camera systems. In the spring of 2015, 2016, and 2017, sound recordings and imagery were collected at a wet meadow and forested slough in the Central Platte River Valley of Nebraska. Boreal chorus frog (*Pseudacris maculata*) calling activity was obtained from analysing sound recordings. Habitat metrics including vegetation phenology and wet meadow hydropattern were derived from image analysis. This dataset can be used to assess phenology, anuran vocalization activity, and environmental change, as well as to further understanding of wetland ecology. Data are co-submitted with manuscript Brinley Buckley, E. M., Gottesman, B. L., Caven, A. J., Harner, M. J., and Pijanowski, B. C., Assessing ecological and environmental influences on Boreal Chorus Frog (*Pseudacris maculata*) spring calling phenology using multimodal passive monitoring technologies. *Ecological Indicators* (In Press).

**Specifications Table**

**Table:** 

Subject	Environmental Science
Specific subject area	Boreal chorus frog (*Pseudacris maculata*) spring calling activity and associated environmental factors
Type of data	Table
How data were acquired	Acoustic recording devices (Wildlife Acoustics SM2), Time-lapse camera systems (TRLcam), NOAA, USGS
Data format	Raw
Parameters for data collection	The datasets were derived from sound recordings, time-lapse imagery, and NOAA/USGS at a wet meadow and slough with varying hydrologic regimes. The study period was focused on the boreal chorus frog breeding period in the spring.
Description of data collection	In the spring of 2015, 2016, and 2017, sound was recorded one minute out of every twenty minutes using Wildlife Acoustics SM2 at two habitats in the Central Platte River Valley of Nebraska. Sound recordings were analyzed to detect Boreal Chorus Frog vocalizations. Time-lapse camera systems were paired with the sound recorders. Vegetation and wet meadow inundation were derived from image analysis. Weather and streamflow variables were obtained from NOAA and USGS, respectively.
Data source location	Buffalo and Hall Counties, Nebraska, United States (40.799, −98.417 and 40.667, −98.892)
Data accessibility	Repository name: Mendeley Data Direct URL to data: http://dx.doi.org/10.17632/p6nbn2hyz9.1
Related research article	Brinley Buckley, E. M., Gottesman, B. L., Caven, A. J., Harner, M. J., and Pijanowski, B. C., Assessing ecological and environmental influences on Boreal Chorus Frog (*Pseudacris maculata*) spring calling phenology using multimodal passive monitoring technologies. *Ecological Indicators* (https://doi.org/10.1016/j.ecolind.2020.107171)

## Value of the Data

• Data describe spring phenological activity of boreal chorus frog calling, vegetation greenness, hydropattern, and weather variables for two wetland types in the Central Platte River Valley of Nebraska. The study sites are within a major migratory bird route and have undergone extensive changes in land and water-use, with implications for wildlife.
• These data are useful for researchers examining anuran activity, phenology, and climate science, as well as land managers interested in wetland hydropattern, associated biological components, and implications for restoration and management.
• Data can be used to assess phenology and phenological changes and provide a baseline reference of boreal chorus frog vocalization activity during the breeding season. Further studies could expand upon the data to understand the influence of ecological factors on anuran calling activity at various temporal and spatial extents by coupling additional data sources.
• Data derived from multimodal monitoring may provide a framework to answer ecological questions and understand complex interactions.

## 1 Data Description

Data consist of daily values for eighteen variables including site (wet meadow or slough), year, date (m/d/yyyy), missing (1 if boreal chorus frog calling activity is missing, 0 otherwise), count (boreal chorus frog calling activity index scaled from 0 to 1), GCC (green chromatic coordinates; vegetation phenology), HYDRO (hydropattern scaled from 0 to 1; water inundation at the wet meadow and Platte River streamflow at the slough), WND (average wind speed; mph), PRCP (daily accumulated precipitation; cm), SNOW (daily snow accumulation; cm), SNWD (total snow depth; cm), TAVG (average temperature; °C), TMAX (maximum temperature; °C), TMIN (minimum temperature; °C), PRCP7 (weekly precipitation accumulation; cm), PRCP1 (one day prior daily precipitation accumulation; cm), DOY (day of the year), and month.

## 2 Experimental Design, Materials and Methods

Wildlife Acoustics SM2 sound recorders were paired with time-lapse camera systems at two sites within the Central Platte River Valley of Nebraska from 1 March 2015 to 1 June 2017. The time-lapse cameras were installed prior to the acoustic recorders as part of the Platte Basin Timelapse Project [1]. The first site, wet meadow habitat, was in open grassland habitat and exhibited variable hydrology. The second site, a slough with more permanent hydrology, was forested and adjacent to a channel of the Platte River.

SM2s with two omnidirectional microphones recorded one minute out of every twenty minutes with a sampling rate of 44.1 kHz, a gain of 36 dB, and a bit depth of 16 bits. Analysis of sound was conducted for days with complete set of recordings (72) using spectral cross-correlation with a window length of 256, a temporal overlap of 75%, and a cutoff threshold of 0.4. Sound recordings were analysed with twelve templates of various frequencies to detect boreal chorus frog activity using the R package monitoR [2]. The resulting dataset is an index of daily boreal chorus frog calling activity scaled from 0 to 1.

Image analysis was performed on imagery between 10:00 and 14:00 to standardize sun exposure. Vegetation phenology was obtained from the time-lapse images at each site by calculating the green chromatic coordinates (GCC = *G*/[*R* + *G* + *B*]) within a region of interest [3]. Water inundation, the hydropattern of the wet meadow, was obtained using a batch macro in ImageJ/FIJI, Java-based open-access program [4,5]. The results are the percent of the image classified as open water, scaled from 0 to 1. Hydropattern at the slough was acquired from USGS streamflow data of the Platte River near Kearney, Nebraska (USGS 06770200). Weather data for the forested slough was obtained from a NOAA station near Kearney, NE (station USC00254335) and at Grand Island, NE (station USW00014935) for the wet meadow [6].

## CRediT Author Statement

**Emma Brinley Buckley:** Conceptualization, Methodology, Data Curation. **Benjamin Gottesman:** Conceptualization, Methodology, Data Curation. **Andrew Caven:** Conceptualization, Review. **Mary Harner:** Funding acquisition, Project administration. **Bryan Pijanowski:** Supervision, Funding acquisition.

## Declaration of Competing Interest

The authors declare that they have no known competing financial interests or personal relationships which have or could be perceived to have influenced the work reported in this article.

## Appendix Supplementary materials

Image, application 1
