# Supplementary material for: Boreal chorus frog (Pseudacris maculata) spring calling activity, habitat metrics, and associated environmental data
Source: Data Brief. 2020 Nov 24;33:106581. doi: 10.1016/j.dib.2020.106581 (PMC7710638; doi:10.1016/j.dib.2020.106581)
Supplement: Supplementary file 1 [file ECOLIND-18486.pdf › ECOLIND-18486.pdf]

Ecological Indicators
Manuscript Draft

Manuscript Number: ECOLIND-18486R1

Title: Assessing ecological and environmental influences on boreal chorus frog (Pseudacris maculata) spring calling phenology using multimodal passive monitoring technologies

Article Type: Research paper

Keywords: Pseudacris maculata; autonomous acoustic recording; wetlands; Central Platte River; multimodal monitoring; image analysis; wet meadows; sloughs

Abstract: Although anuran reproductive behavior follows a phenological pattern, environmental factors may influence the timing of anuran activity. To characterize the spring calling phenology of Pseudacris maculata (boreal chorus frog) we installed acoustic recorders during three spring breeding seasons (2015-2017) within wet meadow and forested slough habitats in central Nebraska near the Platte River. The recorders were paired with time-lapse camera systems that were used to measure vegetation change and wet meadow inundation. We examined the relationship between environmental variables, including temperature, precipitation, and hydropattern, and P. maculata calling activity using random forest models. Calling activity exhibited diel and seasonal variation with differences observed between habitats. Hourly call activity was highest between 20:00 and 01:00, with diurnal and sometimes near-continuous calling observed at the wet meadow but not at the forested slough. Random forest models explained 73% of calling activity variance at the wet meadow and 45% at the forested slough, suggesting additional factors, such as predation, may be influential at the forested slough, as it exhibited near permanent hydrology. P. maculata was predicted to increase chorusing at the 70th day of the year at the wet meadow but not until the 100th at the forested slough. When average air temperatures exceeded 5□C, calling activity was predicted to increase at both wetlands, and calling declined below this temperature. Hydropattern and weekly precipitation accumulation were important predictors of P. maculata calling activity. Our findings highlight the importance of climatic factors such as temperature and precipitation regimes, in addition to habitat conditions such as water-availability, on P. maculata calling activity. Furthermore, this study demonstrates the utility of multimodal passive monitoring technologies, including both visual and auditory tools, for understanding the environmental factors that influence biological activity. Multimodal approaches can be especially useful for documenting and measuring vocal-taxa diversity, activity, and drivers of change.

Response to Reviewers: Reviewer #1: The paper has a great interest for the ecology of Pseudacris maculata.
The major concern regards a very long and detailed discussion that is more adapt for an ecological/biological journal.
Comment: We agree with the reviewer's comments that the discussion is extensive and detailed. We have attempted to condense and reframe the discussion accordingly.

Line 16: "passive" not necessary
Edited

Line 21: Random Forest Models or random forest model
Edited

Line 24: RF is an acronym without previous references. I suggest to use randon forest models
Edited

Line 37: diversity better than biodiversity because is referred to vocal-taxa
Agreed, edited

Line 40: , random forest model,
Edited

Line 66: I suggest to include other references after Gasc
Added Abrahams and Geary 2020

Line 72: it seems that biotic factor are not described
Included habitat, vegetation, hydrology, food availability, and climatic factors

Line 74: "Changes in these factors" this sentence seems obvious and "factors" is really too generic
Agreed, changed to changes in climatic and habitat conditions, suc as an altered hydrologic regime or vegetation phenophase.

Line 79: there are several types of ecotones I suggest "aquatic-terrestrial ecotone"
Agreed, I like the specificity

Line 86: "calling" or "choruses"?
Edited

Line 108: sound recorders and acoustic analysis, do you intend aural survey and acoustic metrics?
Yes, edited

Line 140: I would prefer wetland hydrologic connectivity and wildlife
Edited

Line 145: Boreal Chorus Frog. why not boreal chorus frog
Edited

Line 150: ".. two acoustic monitoring stations" I suggest " two digital autonomous acoustic recorders?
Edited

Line 160 and 167: capital letters seem not necessary in common names Sandhill Cranes, Whooping Cranes.
Edited to lower case

Line 172: acoustic sensors see comment at line 150
Edited

Line 176: please explain better the procedure
Changed to: All incomplete recording days, defined as a day with less than the expected 72 one-minute recordings per 24-hour period, were removed prior to analysis (Now Line 214).

Line 178: Next, stereo tracks were converted to mono by selecting the track
with greater high frequency sensitivity, as rain and other environmental factors can affect microphone sensitivity across mid- to high-frequencies. I don't understand the relationship between the mono conversion and the microphone sensitivity, please rephrase and clarify.
Edited to: Next, stereo tracks were converted to mono. In some cases, the sensitivity of one of the two microphones degraded over the course of deployment, possibly due to rain or other environmental factors. In these cases, we selected the channel of the intact microphone. If there were no microphone performance issues, the channel was randomly selected (Now Line 217).

Line 219: I suggest to anticipate the point 2.3 at 2.2
Switched .2.2 and 2.3

Line 374: The discussion is very long and some narrative results outside the aim of the Journal that should focus on ecological indicators and less on details about the physiology/biology of species. I suggest to reduce the discussion.
Comment: Reduced the discussion, honed in narrative tailored more to aims of journal, and removed extraneous narrative outside natural history details relevant to results (Lines 382-571)

Line 414 random forest not Randon Forest?
Edited

Line 437 WHCR use the acronym close to Whooping Crane for the first time
Edited

Line 493, 573: P. maculata in Italics
Edited

Reviewer #1: The paper has a great interest for the ecology of Pseudacris maculata.
The major concern regards a very long and detailed discussion that is more adapt for an ecological/biological journal.
We agree with the reviewer's comments that the discussion is extensive and detailed. We have attempted to condense and reframe the discussion accordingly.

Line 16: "passive" not necessary
Edited
Line 21: Random Forest Models or random forest model
Edited
Line 24: RF is an acronym without previous references. I suggest to use randon forest models
Edited
Line 37: diversity better than biodiversity because is referred to vocal-taxa
Agreed, edited
Line 40: , random forest model,
Edited
Line 66: I suggest to include other references after Gasc
Added Abrahams and Geary 2020
Line 72: it seems that biotic factor are not described
Included habitat, vegetation, hydrology, food availability, and climatic factors
Line 74: "Changes in these factors" this sentence seems obvious and "factors" is really too generic
Agreed, changed to changes in climatic and habitat conditions, suc as an altered hydrologic regime or vegetation phenophase.
Line 79: there are several types of ecotones I suggest "aquatic-terrestrial ecotone"
Agreed, I like the specificity
Line 86: "calling" or "choruses"?
Edited
Line 108: sound recorders and acoustic analysis, do you intend aural survey and acoustic metrics?
Yes, edited
Line 140: I would prefer wetland hydrologic connectivity and wildlife
Edited
Line 145: Boreal Chorus Frog. why not boreal chorus frog
Edited
Line 150: ".. two acoustic monitoring stations" I suggest " two digital autonomous acoustic recorders?
Edited
Line 160 and 167: capital letters seem not necessary in common names Sandhill Cranes, Whooping Cranes.
Edited to lower case

Line 172: acoustic sensors see comment at line 150
Edited
Line 176: please explain better the procedure

Changed to: All incomplete recording days, defined as a day with less than the expected 72 one-minute recordings per 24-hour period, were removed prior to analysis (Now Line 214).

Line 178: Next, stereo tracks were converted to mono by selecting the track with greater high frequency sensitivity, as rain and other environmental factors can affect microphone sensitivity across mid- to high-frequencies. I don't understand the relationship between the mono conversion and the microphone sensitivity, please rephrase and clarify.

Edited to: Next, stereo tracks were converted to mono. In some cases, the sensitivity of one of the two microphones degraded over the course of deployment, possibly due to rain or other environmental factors. In these cases, we selected the channel of the intact microphone. If there were no microphone performance issues, the channel was randomly selected (Now Line 217).

Line 219: I suggest to anticipate the point 2.3 at 2.2

Switched .2.2 and 2.3

Line 374: The discussion is very long and some narrative results outside the aim of the Journal that should focus on ecological indicators and less on details about the physiology/biology of species. I suggest to reduce the discussion.

Reduced the discussion, honed in narrative tailored more to aims of journal, and removed extraneous narrative outside natural history details relevant to results (Lines 382-571)

Line 414 random forest not Randon Forest?

Edited

Line 437 WHCR use the acronym close to Whooping Crane for the first time

Edited

Line 493, 573: P. maculata in Italics

Edited

Graphical Abstract(for review)

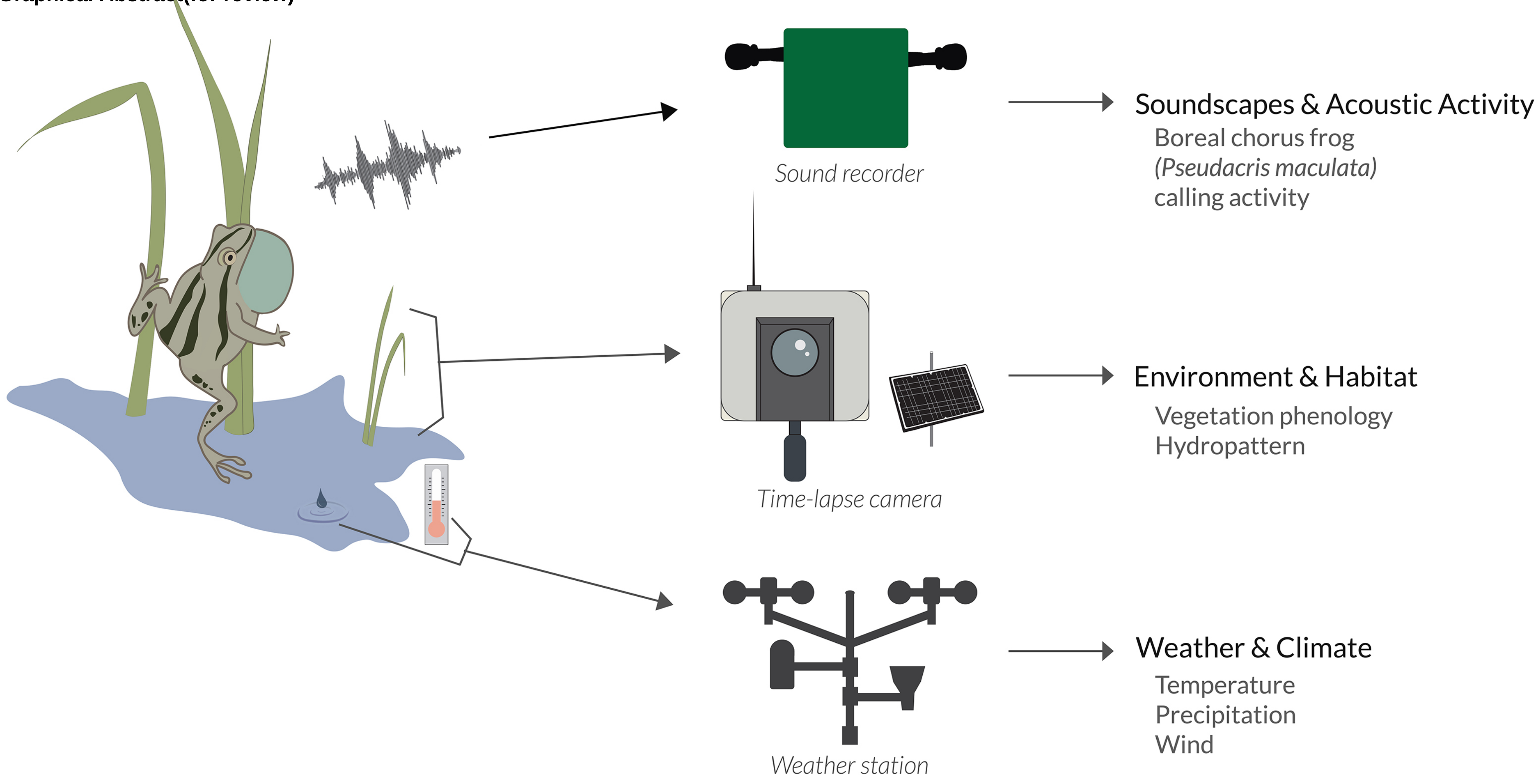

*Highlights (for review)

Highlights

- Multimodal monitoring integrates sound, imagery, sensors to assess variation
- Sound recordings were analyzed for spring boreal chorus frog call activity
- Vegetation phenology, wet meadow hydrology obtained through image analysis
- Hydropattern, vegetation, weekly precipitation predict calling activity
- Multimodal approaches capture patterns in vocal-taxa, habitat, environment

*Manuscript
Click here to view linked References

Assessing ecological and environmental influences on boreal chorus frog (*Pseudacris maculata*) spring calling phenology using multimodal passive monitoring technologies

Emma M. Brinley Buckley[1*], Benjamin L. Gottesman[2], Andrew J. Caven[3], Mary J. Harner[1,4], Bryan C. Pijanowski[2]

[*]Corresponding Author, brinleybuckleyem@unk.edu
[1]Department of Communication, University of Nebraska at Kearney, Kearney, NE 68849
[2]Center for Global Soundscapes and Department of Forestry & Natural Resources, Purdue University, West Lafayette, IN 47907
[3]Platte River Whooping Crane Maintenance Trust, Wood River, Nebraska 68883
[4]Department of Biology, University of Nebraska at Kearney, Kearney, NE 68849

Abstract

Although anuran reproductive behavior follows a phenological pattern, environmental factors may influence the timing of anuran activity. To characterize the spring calling phenology of *Pseudacris maculata* (boreal chorus frog) we installed acoustic recorders during three spring breeding seasons (2015-2017) within wet meadow and forested slough habitats in central Nebraska near the Platte River. The recorders were paired with time-lapse camera systems that were used to measure vegetation change and wet meadow inundation. We examined the relationship between environmental variables, including temperature, precipitation, and hydropattern, and *P. maculata* calling activity using random forest models. Calling activity exhibited diel and seasonal variation with differences observed between habitats. Hourly call activity was highest between 20:00 and 01:00, with diurnal and sometimes near-continuous calling observed at the wet meadow but not at the forested slough. Random forest models explained 73% of calling activity variance at the wet meadow and 45% at the forested slough, suggesting additional factors, such as predation, may be influential at the forested slough, as it exhibited near permanent hydrology. *P. maculata* was predicted to increase chorusing at the 70th day of the year at the wet meadow but not until the 100th at the forested slough. When average air temperatures exceeded 5°C, calling activity was predicted to increase at both wetlands, and calling declined below this temperature. Hydropattern and weekly precipitation accumulation

were important predictors of *P. maculata* calling activity. Our findings highlight the importance of climatic factors such as temperature and precipitation regimes, in addition to habitat conditions such as water-availability, on *P. maculata* calling activity. Furthermore, this study demonstrates the utility of multimodal passive monitoring technologies, including both visual and auditory tools, for understanding the environmental factors that influence biological activity. Multimodal approaches can be especially useful for documenting and measuring vocal-taxa diversity, activity, and drivers of change.

Keywords: *Pseudacris maculata*, autonomous acoustic recording, wetlands, Central Platte River, multimodal monitoring, image analysis, wet meadows, sloughs

1. Introduction

The relationship between animals and their environment is synchronously intertwined, as biotic and abiotic conditions influence the timing of important animal behaviors (Johansson et al. 2015). In temperate regions, amphibians such as anurans have adapted life history strategies aligned with local climatic and physical conditions (Lofts 1974, Amburgey et al. 2012). Over the last four decades, anuran populations have declined globally due to compounding factors including changes in land and water use, habitat loss, pathogens, and a changing climate (Sodhi et al. 2008, Grant et al. 2016, Green et al. 2020). In the Great Plains, amphibians are declining by an estimated >3% annually, however, information on anuran phenology and environmental drivers of anuran behavior in the region is sparse (Mushet et al. 2012, Grant et al. 2016). Often anuran research, including trapping, manual listening, and visual encounter surveys, is conducted for short periods at infrequent intervals (Bridges and Dorcas 2000). For elusive yet vocal

animals, such as small and mostly nocturnal anurans, auditory surveys have proven beneficial for monitoring, for example the North American Amphibian Monitoring Program (Weir and Mossman 2005). As their main form of communication, anurans vocalize predominately to attract mates and alert rival males to their presence (Bee et al. 2010). Manual listening surveys of anuran calling frequency, intensity, and number (usually in the form of scaled indices) can be used to model species behavioral patterns, spatial distribution, temporal occurrence, and responses to environmental change (Stevens et al. 2002, Nelson et al. 2004, Both et al. 2008). Although these methods can provide important information about long term trends and change, the data is often temporally limited, which can be problematic considering the high variability in anuran calling activity (Bridges and Dorcas 2000, Todd et al. 2003). Passive acoustic recording, which is increasingly used to monitor and assess changes in soundscapes (Pijanowski et al. 2011), ecosystem state (Sueur and Farina 2015), and vocalizing taxa including bird communities (Gasc et al. 2017, Abrahams and Geary 2020) and bat activity (Whitby et al. 2014, Reichert, et al. 2018), may offer an alternative with higher temporal resolution at hourly or daily intervals (Digby et al. 2013, Sugai et al. 2019). Moreover, this technology remains comparatively underutilized for anurans considering its potential benefits (Bee et al. 2010, Madalozzo et al. 2017, Brodie et al. 2020).

Anuran calling activity exhibits a phenological pattern, occurring during the breeding season but influenced by biotic and abiotic factors, including habitat, vegetation, hydrology, food availability, and climatic factors (Oseen and Wasserug 2002, Klaus and Lougheed 2013, Ospina et al. 2013). Changes in climatic and habitat conditions, such as an altered hydrologic regime or vegetation phenophase, can result in phenological shifts of behavior, such as reproduction , leading to potential resource mismatches (Klaus and Lougheed 2013). Studies have consistently

found that temperature influences the lifecycle and activity of ectothermic anurans, however even similar species have shown differential responses to temperature regimes (Oseen and Wassersug 2002, Hillman et al. 2009, Steelman and Dorcas 2010). Many anuran species are dependent upon aquatic-terrestrial ecotones, utilizing terrestrial habitat during their adult phase and aquatic habitat during reproduction and metamorphose, and therefore, water availability generally plays an important role in reproductive behavior (Ryan and Winne 2001, Amburgey et al. 2012). However, the magnitude and mechanism of influence, such as precipitation or hydropattern (the hydroperiod, hydrologic fluctuation, timing, and flow regime of a system; Mathews et al. 2010, Amburgey et al. 2014), likely differs between ephemeral and permanent or semi-permanent wetlands, as well as geographic regions, and by species (Werner et al. 2009, Ospina et al. 2013). In addition to the hydropattern of a system, habitat conditions such as vegetation structure may influence calling, as many anurans, including *Pseudacris maculate* (boreal chorus frog), depend on plants for protection from predators, cling to vegetation during chorusing, as well as lay egg masses beneath vegetation (Conant and Collins 1991, Andersen et al. 1999, Grant et al. 2019).

P. maculata, a small (~3.3 cm) trilling chorus frog, is found in emergent wetlands and grasslands across central parts of the United States and Canada, with statewide distribution in Nebraska (Fogell 2010). Populations of *P. maculata* are broadly considered stable, however, declines in *Pseudacris* species (*sensu lato*, trilling chorus frogs) have been observed in several regions, including the Great Plains, Midwest, eastern Ontario, western Quebec, as well as possible extirpation from New York and Vermont (Knutson et al. 2000, Seburn et al. 2014). Studies show that *P. maculata* are exposed to a myriad of threats, including human influences, disease, toxins, and climatic factors (Grant et al. 2016), and populations are impacted by drought

and flooding (Werner et al. 2009, Grant et al. 2019). In the Great Plains, *P. maculata* chorusing is often one of the earliest vocalizations heard and an iconic sign of spring, with breeding taking place as early as March and continuing as late as May with occasional breeding activity into the summer following large precipitation events (Jones et al. 1981, Fogell 2010). As *P. maculata* exhibits an early spring phenological pattern, likely driven by weather and habitat conditions, pairing auditory and visual autonomous technologies can provide documentation of local biotic and environmental changes at various scales, which may help to disentangle complex ecological interactions (Buxton et al. 2018).

In this study, we aimed to assess how *P. maculata* calling activity related to local environmental conditions in central Nebraska during three spring breeding seasons (2015-2017) using multimodal passive monitoring technologies. We used aural surveys, acoustic metrics, and automated classification of sound recordings to examine hourly and daily call activity patterns and characterize variability in *P. maculata* spring calling phenology. Image analysis was used to quantify changes in hydroperiod and vegetation. We assessed the importance of hydropattern (inundation and river streamflow), vegetation, temperature, wind speed, precipitation, and snowfall on *P. maculata* activity using random forest models. We examined the call activity of *P. maculata* within wet meadow habitat compared to a semi-permanent forested wetland. Combining acoustic and visual monitoring technologies, our research attempted to gain insight into the phenological patterns of *P. maculata* spring calling activity and to identify important environmental factors influencing activity.

2. Methods

2.1. Study Area

The study was conducted in the Central Platte River Valley (CPRV) of Nebraska, an important avian stopover location within the Central Flyway migration corridor (Vrtiska and Sullivan 2009, Pearse et al. 2011, Caven et al. 2019a). The region is used by endangered whooping cranes, hundreds of thousands of sandhill cranes, and millions of other waterbirds as a stopover to rest and refuel by foraging on vegetation, fish, insects, and anurans (Lingle et al. 1991, LaGrange 2005, Vrtiska and Sullivan 2009, Geluso et al. 2013, Caven et al. 2019b, Caven et al. 2020). The Platte River extends the reach of tallgrass prairie habitat west into the mixed-grass ecoregion in central Nebraska as a result of subirrigation, and relatively minor elevational changes within this riverine ecosystem provide for a dynamic set of wetland habitats with varying hydroperiods (Currier 1995, Whiles and Goldowitz 2005, Kaul et al. 2011, Meyer and Whiles 2010). Wet meadows represent more ephemeral wetlands with shorter hydroperiods that usually experience inundation through the early spring, while shallow and deep marsh habitats experience increasing hydroperiods lasting into the summer or even throughout the growing season in some years (Kantrud et al. 1989, Currier 1995, Whiles and Goldowitz 2005, Tiner 2016). These wetlands are connected to the hydrology of the Platte River, which experiences high flows in the spring from snowmelt with declining streamflow into the summer (Williams 1978, Eschner et al. 1983, Currier and Henszey 1996). Historically, the Platte River was a wide braided river that sustained a large number of riparian wetlands within a grassland mosaic (Smith 1971, Williams 1978, Currier and Henszey 1996). However, over the last century, extensive land and water use changes have altered the landscape, negatively impacting river hydrogeomorphology, wetland hydrologic connectivity, and wildlife (Sidle et al. 1989, Eschner et al. 1983, Horn et al. 2012, Alexander et al. 2020). Conversion of grassland and wetland habitats to agriculture and diversions for irrigation has removed or retimed the majority of

streamflow in the Platte River and erased over 70% of wet meadows and other wetlands from the CPRV (Williams 1978, Eschner et al. 1983, Sidle et al. 1989, Currier and Henszey 1996). Understanding how hydrologic and environmental fluctuations influence boreal chorus frog activity in the remaining high-quality wetlands should further inform habitat conservation and restoration efforts focused on preserving this species.

2.2. Environmental Variables

Weather data was acquired from the National Oceanic and Atmospheric Administration, National Centers for Environmental Information, Global Historical Climatology Network–Daily database (Menne et al. 2012). We obtained data from Kearney, NE (station USC00254335) for the forested slough and Grand Island, NE (station USW00014935) for the wet meadow. Daily variables included average wind speed, total precipitation, mean, maximum, and minimum air temperature, snow accumulation and snow depth. Average wind speed was only available at the Grand Island weather station and was therefore used for both study sites. Hydropattern relating to the forested slough was obtained from a USGS stream gage for the Platte River at Kearney, Nebraska (USGS 06770200).

We derived vegetation phenology and water inundation data from analysis of time-lapse images from Mormon Island and Rowe Sanctuary Tower cameras of the Platte Basin Timelapse project, a long-term monitoring and science communication project documenting the Platte Basin (www.plattebasintimelapse.com). The time-lapse camera systems, consisting of a Nikon D300 DSLR with a 12.3 megapixel crop sensor and Nikkor 18-70mm 1:3.5-4.5 lens set at F/8 and powered by a solar panel, took one image every hour of sunlight. Images were processed by selecting daily images taken between 10:00 and 14:00 to standardize for sun exposure and shadows. We then visually inspected the imagery collections, and images with obstructed views or inconsistencies, such as fog or spider webs, were removed from the analysis. Based on the

digital number (DN) within the red-green-blue (RGB) colorspace of a pixel, we calculated green chromatic coordinates (GCC), a greenness index of vegetation phenology (Sonnentag 2012). At each site, we selected a region of interest (ROI) that encompassed vegetation and excluded sky and other features within the frame of view. GCC was calculated for each site's ROI using the equation GCC = G/[R + G + B] (Richardson 2018).

For the wet meadow at Mormon Island, we assessed water inundation through image-analysis using a macro script adapted from microscopy in the Java-based open-access program FIJI (Schindelin et al. 2012, Brinley Buckley et al. 2017). A ROI, separate from the ROI used to calculate GCC, was selected that excluded sky, horizon, and additional grassland landscape to reduce variability. The RGB colorspace was transformed to hue-saturation-value (HSV) to overcome potential limitations of the RGB color space for classifying water (Pekel et al. 2014). The ROI was converted to binary (0,1) by automated thresholding (threshold = (average background + average objects)/2). The binary masked results were visually inspected and manually compared to the original image for accuracy, and if a mask was visibly inaccurate, the original image was classified by manually tracing water inundation. A proxy for ponding surface water, the resulting water inundation (WI) values were the percent area classified as water within the region of interest. The final dataset was averaged into daily values and rescaled to a 0 to 1 value.

2.3. Acoustic Analyses

We installed two digital autonomous acoustic recorders paired with time-lapse camera systems within the CPRV from 1 March 2015 to 1 June 2017. Recorders were deployed next to the cameras approximately 1.5 m above the ground. The first paired monitoring system was within Mormon Island, a wet meadow habitat between two channels of the Platte River in southcentral Nebraska (40.799, -98.417). The site is generally devoid of trees and dominated by

sedges (*Carex* spp.), common threesquare (*Schoenoplectus pungens*), and slimstem reedgrass (*Calamagrostis stricta*). The site is managed using patch-burn-grazing to simulate natural disturbances (See Fuhlendorf et al. 2009) by the Crane Trust for the benefit of sandhill cranes, whooping cranes, other migratory birds, and overall biological diversity. The second site was a wetland slough, which is a linear swale supporting wetland habitat (Meyer and Whiles 2008), in this case predominantly deep marsh, at Audubon's Rowe Sanctuary in Gibbon, Nebraska (40.667, -98.892). The slough exists within a former channel of the Platte River in a complex of accumulated sediment and deposited organic material, resulting from reduced flows over the last century, and one bank includes an erosion control structure built before 1938 as indicated by aerial photographs. The site is dominated by cattails (*Typha* spp.) and is immediately surrounded by trees including green ash (*Fraxinus pennsylvanica*), plains cottonwood (*Populus deltoides*) and woody shrubs including false indigo bush (*Amorpha fruticosa*). This forested slough site sits 22 m south of the Platte River's main (south) channel and retains standing water nearly year-round, while the wet meadow site rarely contains standing water beyond May (Brinley Buckley et al. *In Review*).

The two autonomous acoustic recorders (Song Meter SM2+; Wildlife Acoustics) recorded for one minute out of every twenty minutes, starting on the hour, for a total of three recordings per hour. The sampling rate was 44.1 kHz, the gain was 36 dB, and the bit depth was 16 bits. Each recorder had two omnidirectional microphones with a flat frequency response from 20 Hz –20 kHz. All incomplete recording days, defined as a day with less than the expected 72 one-minute recordings per 24-hour period, were removed prior to analysis. Recordings were then down-sampled to 8 kHz to accelerate computation time, as *P. Maculata* vocalizations average between 2.7 and 4.2 kHz throughout their range (Bee et al. 2010). Next, stereo tracks were

converted to mono. In some cases, the sensitivity of one of the two microphones degraded over the course of deployment, possibly due to rain or other environmental factors. In these cases, we selected the channel of the intact microphone. If there were no microphone performance issues, the channel was randomly selected. We quantified the acoustic activity of chorus frogs by detecting their calls with spectral cross-correlation. This method measures the pixel-by-pixel similarity between the spectrogram of a template call and the spectrogram of windowed selections of a recording (Hafner and Katz, 2018). This method is particularly effective for species with stereotypical calls, such as the characteristic call of *P. maculata,* a rising trill that sounds like fingers running across a comb. However, its call frequency, structure, and rate vary depending on environmental factors like temperature as well as physical characteristics (Bee et al. 2010). *P. maculata* also form choruses, where the calls of different individuals overlap. To accommodate this variation, we created 12 templates for the analysis consisting of a range of observed vocalizations (e.g. different fundamental frequency, call lengths, chorus densities; Table 1). To help quantify the diversity of call templates, we measured their center frequencies and inter-pulse distances in Raven 1.6.1 using spectrograms that had a window length of 256 and a Hann window (Raven Pro: Interactive Sound Analysis Software, 2019). Center frequency refers to the frequency that divides a template recording into two frequency intervals containing equal energy. Inter-pulse distance refers to the interval between consecutive pulses within a call, from the offset of one pulse to the onset of the next; it was manually measured only for non-chorus templates by taking the mean of three inter-pulse intervals per clip. We used the R package “monitoR” (Hafner and Katz, 2018) to perform the spectral cross-correlation, utilizing a window length of 256 and a temporal overlap of 75%. Correlation peaks above a cutoff threshold of 0.4 were counted as detections. As we utilized 12 call templates, there was potential that

different templates would detect the same chorus frog call, inflating the total count. To avoid duplicating detections, we divided each recording into 240, 0.25-second windows and counted a detection if the 0.25-second window contained one or more detections. To assess the accuracy of detection algorithms, we assessed their true positive rates by generating 1000 random files from February-May and manually identifying if *P. maculata* was detected via listening and visual inspection of spectrograms.

Template	Type	Low frequency (kHz)	High Frequency (kHz)	Center Frequency (kHz)	Duration (s)	Inter-Pulse Distance (s)
**Frog 1**	Chorus	2.5	4.0	3.2	0.30	NA
**Frog 2**	Chorus	2.7	3.6	3.1	0.30	NA
**Frog 3**	Chorus	2.6	4.0	3.3	0.31	NA
**Frog 4**	Chorus	2.6	4.2	3.3	0.30	NA
**Frog 5**	Chorus	2.6	4.0	3.2	0.30	NA
**Frog 6**	Individual	2.8	4.5	3.6	0.30	0.05
**Frog 7**	Individual	3.0	3.9	3.4	0.30	0.06
**Frog 5**	Individual	2.7	3.6	3.1	0.25	0.04
**Frog 9**	Individual	2.8	4.0	3.2	0.30	0.06
**Frog 10**	Individual	2.6	3.6	3.1	0.15	0.01
**Frog 11**	Individual	2.7	3.6	3.1	0.15	0.02
**Frog 12**	Individual	2.9	3.6	3.2	0.16	0.03

Table 1: Bioacoustic information for the 12 *Pseudacris maculata* call templates used in "monitoR", including call type, lower and upper frequency bounds (of the template, not the call itself), center frequency, duration (of the template, not the call itself), and inter-pulse distance. Center frequency and inter-pulse distance were calculated using Raven 1.6.

As detections did not indicate or attempt to approximate a count or true abundance of *P. maculata*, vocalization detections were scaled to create a calling activity index from 0 to 1, where 0 indicates no vocalizations and 1 indicates the highest calling activity of *P. maculata* chorusing during the study. This represents a logical rescaling of count data to a ratio scale, which may help improve interpretation of our abundance index, which is both a reflection of local density as well as activity level (Schneider 2009, Pijanowski et al. 2011).

2.4. Statistical Analysis

All analyses characterizing the hourly and daily call activity of *P. maculata* were conducted within R (R Development Core Team, 2020). To assess diel activity we calculated how many recordings had at least one *P. maculata* vocalization identified out of 72 recording intervals per day, or the calling activity frequency. We set a threshold of 0.8 to represent a high

frequency of calling, indicative of near 24-hour continuous call activity, and calculated monthly averages. We calculated summary statistics for all variables, in addition to calculating Spearman's Rank Correlation Coefficients to assess daily calling activity in association with environmental variables.

We used random forest regression to determine factors of importance for *P. maculata* calling activity using the "randomForest" package (Liaw and Wiener 2002). Random forest (RF) is a machine learning technique based on averaging a large collection of decision trees. Each tree is trained on a random bootstrap sample, while each split within a tree is sampled from a subset of candidate variables, making RF less susceptible to overfitting, more robust to correlations, and reducing variance compared to a single decision tree (Breiman 2001). RF models are non-parametric, non-linear, and can handle numerous continuous and categorical predictor variables. In addition, they can handle complex data with relatively limited observations (Cutler et al. 2012), a common issue with ecological data. RF provides a permutation-based measure of variable importance (VIMP), using mean squared error (MSE) for regression, which denotes the decrease in model accuracy given a variable is removed from the model. We built site-specific RF models with all candidate predictor variables including wind (WND; average wind speed; mph), precipitation (PRCP; daily accumulation; cm), 7-day precipitation (PRCP7; weekly accumulation; cm), 1-day lag precipitation (PRCP1; day prior daily accumulation; cm), daily mean, maximum, and minimum air temperature (TAVG, TMAX, TMIN; °C), day of year (DOY), green chromatic coordinates (GCC), snow (SNW; daily accumulation; cm), snow depth (SNWD; cm), and included year to account for annual variability. Hydropattern was also included in the models, with water inundation (WI) included for the wet meadow and Platte River streamflow (CMS; m^3/s) at the forested wetland. The number of variables selected as

candidates at each split was set to three, and the model was tested and pruned to 500 trees to reduce overfitting. Variables were ranked by variable importance score, and variables with negative MSE scores, indicating they negatively influenced prediction accuracy, were removed from the model. Relationships between environmental variables and *P. maculata* activity were examined using partial dependence plots, which predicts the response of *P. maculata* to a given variable holding all other variables in the model at their mean.

3. Results

From 2015 to 2017 we totaled 472 recording days (227 at wet meadow/Mormon and 245 at forested slough/Rowe) during the spring breeding season (March – June). During the three years of our study, data were missing for 49 days at the wet meadow (18%) and 31 days at the forested slough (11%) due to equipment malfunction or removal of days with incomplete recording intervals. Assessment of detection algorithms resulted in an overall true positive (TP) rate of 97.8% (n=1000). At Mormon Island, the TP rate was 98.4% (n=853), and at the forested slough, the TP rate was 94.6% (n=147). For the sampling duration, mean daily average temperature was approximately 8 °C, ranging from approximately -15 to 24 °C (Table 2). Mean daily precipitation accumulation was approximately 0.2 cm and ranged from 0 to 5.23 cm at Mormon and 0 to 10.16 cm at Rowe, with highest mean and median precipitation in 2016 (Figure 1). Mean streamflow for the Platte River was 85 m^3/s, with the highest mean and median streamflow occurring in 2016 (107 m^3/s; Figure 1). Mean streamflow was highest in May followed by March, with the highest variability in May 2015. Contrastingly, wet meadow inundation had the highest mean in March of 2016 and 2017 and was relatively similar across months in 2015, while inundation had the greatest range in April of all years (Figure 1). The wet

meadow had the highest mean inundation in 2016 (0.51), in addition to the highest peak extent of inundation (Table 2).

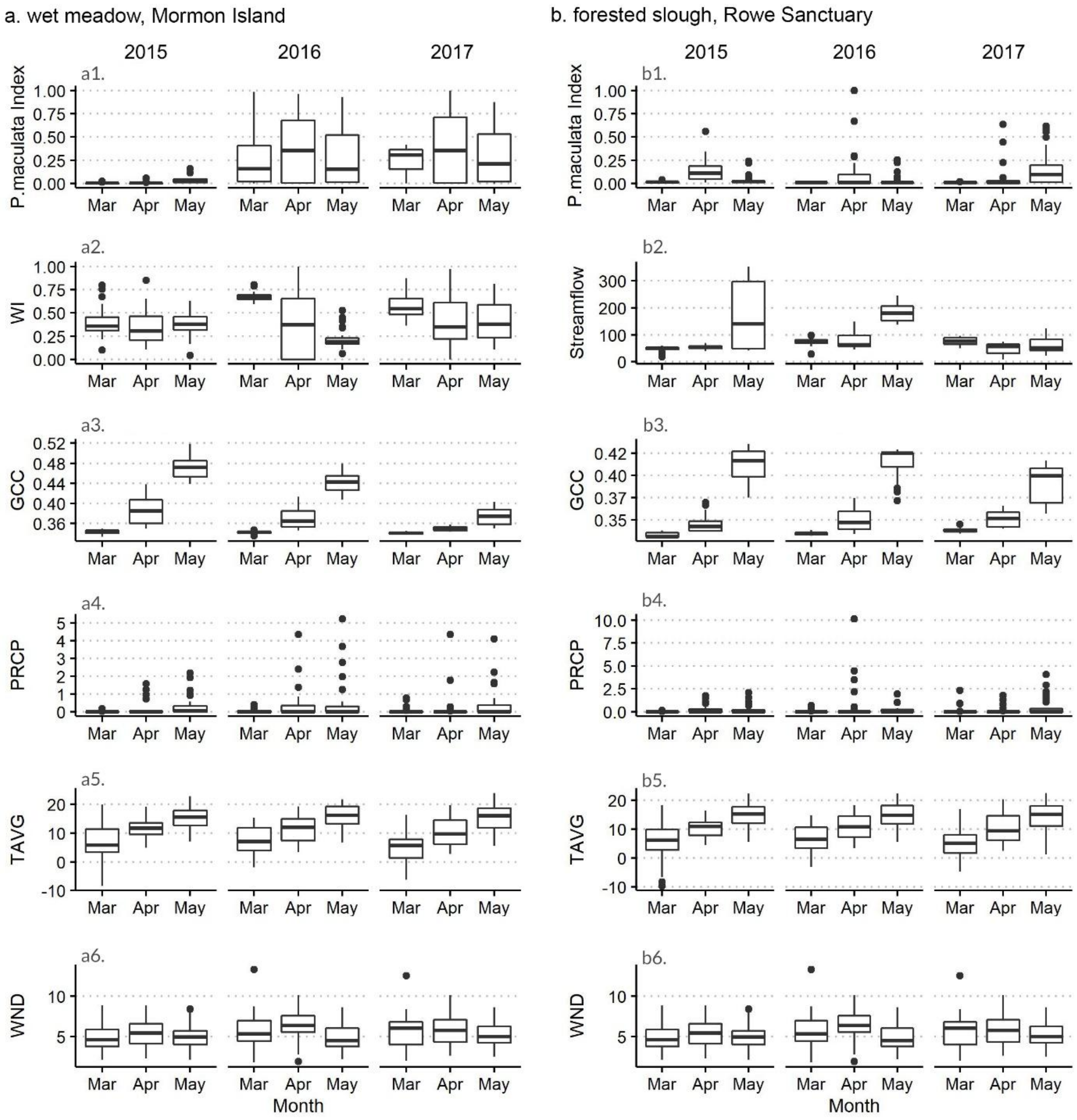

Figure 1. Monthly boxplots at wet meadow habitat (a.) and forested slough (b.) illustrating the distribution, median, and outliers for *Pseudacris maculata* calling activity index [a1, b1], hydropattern (WI; water inundation [a2], CMS; streamflow CMS[b2]), vegetation (GCC; green chromatic coordinates [a3,b3]), precipitation (PRCP [a4,b4]), temperature (TAVG [a5,b5]), and wind speed (WND [a6,b6]).

Wet meadow

		Count	WND	PRCP	TAVG	GCC	WI
2015	min	0	2.1	0	-15.3	0.33	0.04
	max	0.16	11.2	2.18	22.9	0.52	0.85
	mean	0.01	5.16	0.14	7.3	0.38	0.45
	sd	0.03	1.71	0.38	8.98	0.06	0.2
2016	min	0	0	0	-8.7	0.33	0
	max	0.98	15.2	5.23	21.7	0.48	1
	mean	0.29	5.75	0.3	9.19	0.37	0.51
	sd	0.32	2.12	0.89	6.98	0.04	0.29
2017	min	0	2	0	-8.2	0.33	0
	max	1	12.5	4.34	24	0.4	0.97
	mean	0.23	5.47	0.19	8.61	0.35	0.44
	sd	0.31	1.82	0.65	7.31	0.02	0.2
All years	min	0	1.8	0	-15.3	0.33	0
	max	1	15.2	5.23	24	0.52	1
	mean	0.19	5.46	0.21	8.37	0.37	0.47
	sd	0.29	1.9	0.68	7.82	0.04	0.23

Forested slough

		Count	WND	PRCP	TAVG	GCC	CMS
2015	min	0	2.1	0	-15.85	0.33	18.15
	max	0.56	11.2	2.08	22.25	0.44	353.96
	mean	0.08	5.16	0.14	6.87	0.36	85.52
	sd	0.11	1.71	0.37	8.82	0.04	78.85
2016	min	0	1.8	0	-7.75	0.33	28.6
	max	1	15.2	10.16	22.2	0.43	246.64
	mean	0.05	5.75	0.24	8.4	0.36	107.26
	sd	0.14	2.12	1.08	6.68	0.04	50.13
2017	min	0	2	0	-8.85	0.33	8.72
	max	0.63	12.5	4.06	22.5	0.42	124.03
	mean	0.06	5.47	0.21	8.38	0.35	62.67
	sd	0.14	1.82	0.62	7.19	0.02	21.92
All years	min	0	1.8	0	-15.85	0.33	8.72
	max	1	15.2	10.16	22.5	0.44	353.96
	mean	0.06	5.46	0.19	7.88	0.36	85.21
	sd	0.13	1.9	0.75	7.63	0.03	58.18

Table 2. Summary statistics (min = minimum, max = maximum, mean, and sd = standard deviation) for *Pseudacris maculata* calling activity (Count), average wind speed (WND), daily precipitation accumulation (PRCP), average air temperature (TAVG), and green chromatic coordinates (GCC). Hydropattern also was included as water inundation (WI) at the wet meadow (Mormon Island) and streamflow (CMS) at the forested slough (Rowe Sanctuary).

For the duration of the study, *P. maculata* vocalizations on average were highest between 20:00 and 01:00 (8:00pm to 1:00am) at both sites, and activity decreased between 01:00 and 02:00 markedly (Figure 2). Calling activity increased moderately between 08:00 and 15:00 in the wet meadow, however this trend was not observed at the forested wetland. Monthly averaged calling frequency, with a threshold of 0.8, also differed between habitats. At the wet meadow, *P. maculata* called near continuously 18% of days in March, 30% of days in April, and 37% of days in May. Comparatively, at the forested slough, *P. maculata* called continuously 0% of days in March, 9% of days in April, and 6% of days in May (Figure 3). In addition, calling frequency differed by year, with nearly continuous calling occurring at the wet meadow more than 40% of the time in April and May of 2016 and 2017 (Figures 1 and 3).

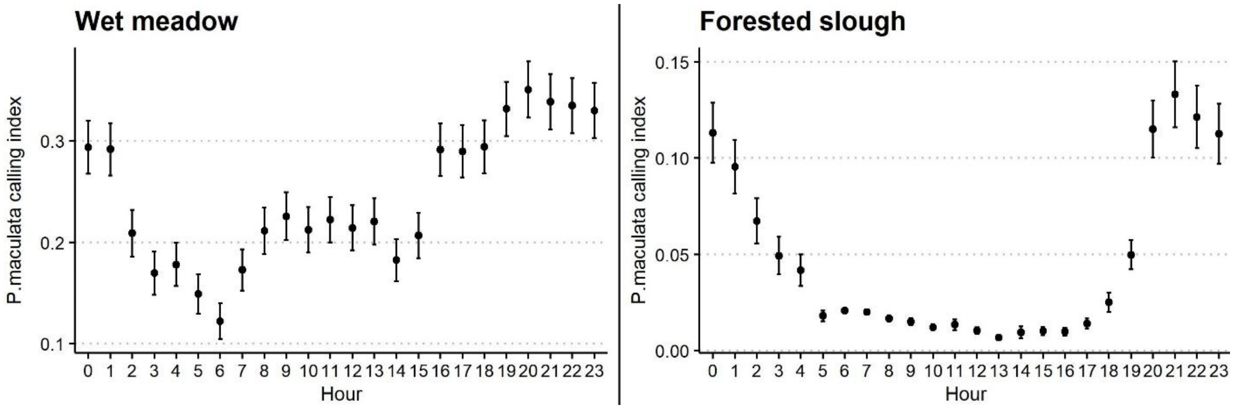

Figure 2. Mean hourly calling activity for Pseudacris maculata during spring (March – June) of 2015 – 2017 within wet meadow habitat on Mormon Island and forested slough habitat at Rowe Sanctuary. Of note, y-axis scale differs for wet meadow (0.1 – 0.3) and forested slough (0 – 0.15).

At both sites, *P. maculata* calling activity was highest during April of all years, with the exception of the forested slough in 2017, where vocalizations were greater in May (Figure 3). Generally, calling activity was sustained from March to May, with vocalizations beginning on average the first week of March (mean DOY 60), peaking from early to mid-April depending on

site (mean DOY 92 at Mormon, mean DOY 111 at Rowe), with a second peak occurring the first week of May (mean DOY 124).

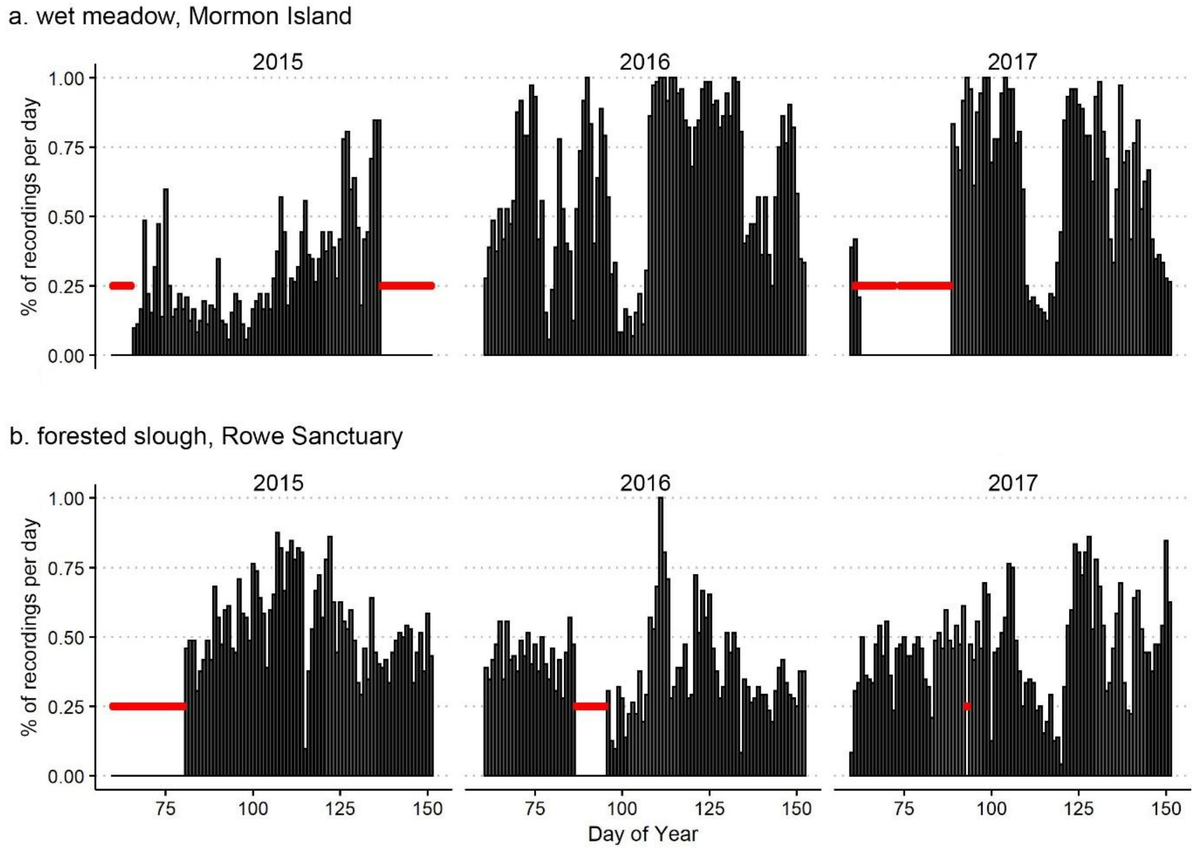

Figure 3. Percent of recordings with *Pseudacris maculata* detections present per day at a wet meadow (a.) and forested slough (b.) in the Central Platte River Valley of Nebraska. Red lines indicate missing data.

Snow and snow depth negatively contributed to the predictive accuracy of the random forest models at both sites and therefore were removed from the final models. Once the models were adjusted, the RF model explained 73% of the variance in *P. maculata* calling activity at the wet meadow (MSE = 0.02), while for the forested slough, the RF model explained 45.3% (MSE = 0.01) (Table 3). Year, day of year, hydropattern, green chromatic coordinates, average temperature, and 7-day total precipitation were the highest ranked variables of importance at both sites, indicating removal of these variables from the models would decrease predictive accuracy substantially. *P. maculata* calling activity was predicted to begin increasing at DOY 60

and then substantially at DOY 70 at the wet meadow and to increase more gradually at DOY 60 followed by a substantial increase at DOY 90 at the forested slough (Figure 4). Similarly, at both sites activity was predicted to begin increasing at temperatures above 5°C, and in response to GCC, peak at 0.35. Activity increased at seven-day precipitation accumulation levels of approximately 0.25 cm at the wet meadow and at approximately 3 cm at the forested slough. Hydropattern was an important variable at both sites, with *P. maculata* activity predicted to increase with inundation levels above 0.2 at the wet meadow and predicted activity peaking at streamflow of approximately 50 m^3/s, declining with increases thereafter. There was also an observed difference in activity based on year, with activity highest in 2016 and 2017 at the wet meadow, and 2015 and 2017 at the forested slough.

a. Wet meadow		b. Forested wetland	
Mean of squared residuals: 0.02		*Mean of squared residual: 0.01*	
Variance explained: 72.7%		*Variance explained: 45.3%*	
Variable	% Inc. MSE	Variable	% Inc. MSE
Year	36.5	PRCP7	20.13
DOY	31.9	GCC	13.42
WI	28.5	DOY	12.85
GCC	22.0	CMS	10.56
PRCP7	18.9	Year	8.08
TAVG	17.9	TAVG	7.4
TMIN	15.8	PRCP1	5.08
TMAX	13.6	TMIN	4.46
PRCP1	5.2	TMAX	4.42
PRCP	4.0	WND	4.07
WND	3.1	PRCP	2.64

Table 3. Results of random forest models predicting *Pseudacris maculata* calling activity at a wet meadow and a forested slough. Variable importance scores ranked from most important (greatest percent increase in mean squared error) to least for all eleven predictor variables. Predictor variables including wind (WND; average wind speed; mph), precipitation (PRCP; daily accumulation; cm), 7-day precipitation (PRCP7; weekly accumulation; cm), 1-day lag precipitation (PRCP1; day prior daily accumulation; cm), daily mean, maximum, and minimum air temperature (TAVG, TMAX, TMIN; °C), day of year (DOY), green chromatic coordinates (GCC), snow (SNW; daily accumulation; cm), snow depth (SNWD; cm), and year (Year).

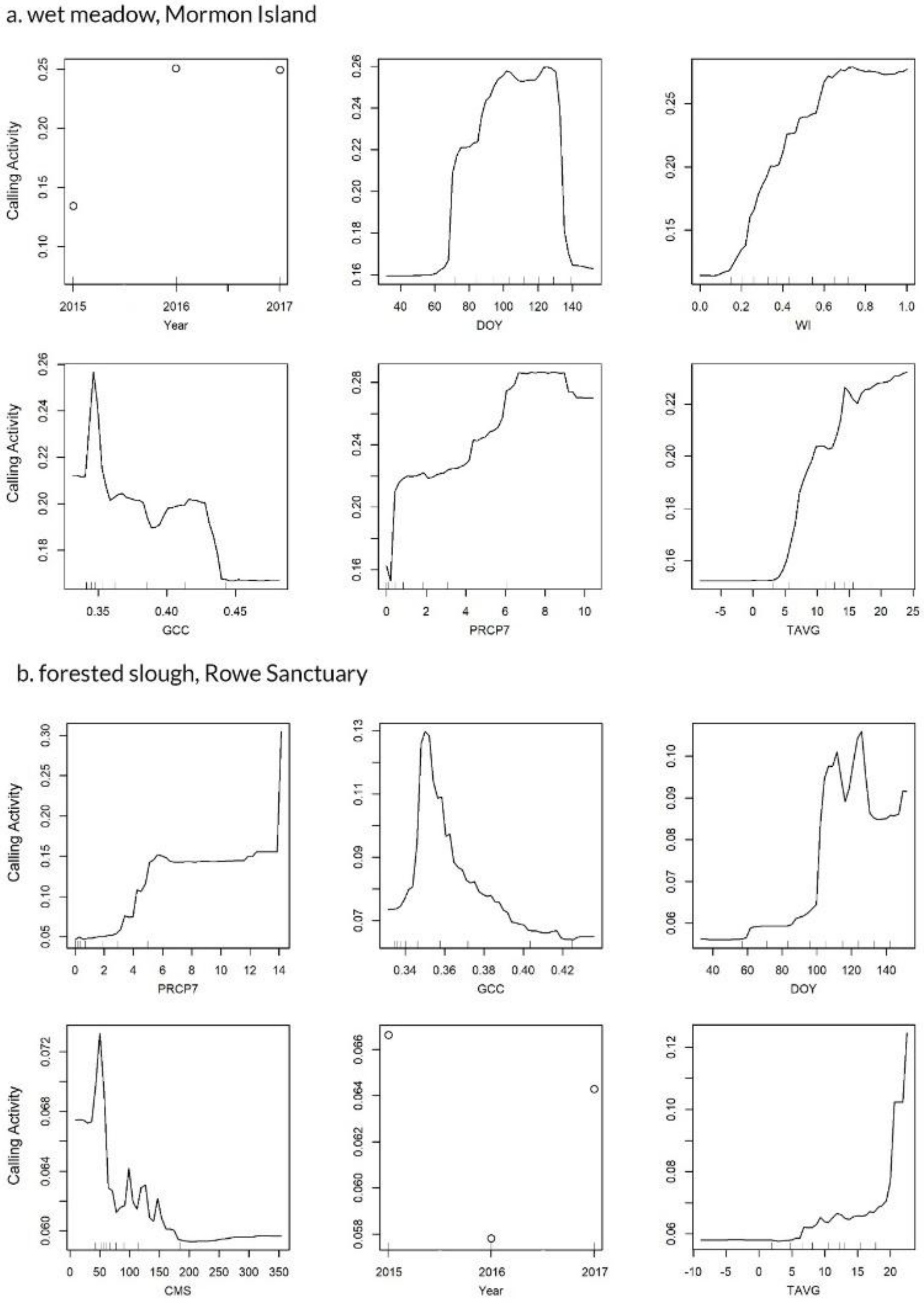

Figure 4. Partial dependence plots showing predicted response of *Pseudacris maculata* to the six variables with the highest variable importance scores from random forest models at a wet meadow (Mormon) and forested slough (Rowe) in central Nebraska.

4. Discussion

In the Great Plains, the life cycle and behavioral activity of *Pseudacris maculata* is closely tied to the grasslands and interspersed wetlands. However, these habitats face increasing pressure from human development, agriculture, water-use changes, and a globally changing climate (Sidle 1989, LaGrange 2005, Matthews et al. 2013). Multimodal monitoring—the pairing of autonomous sound recorders, time-lapse cameras, and additional sensors—collects data streams capable of reflecting changes in environmental and habitat conditions, as well as vocal-taxa communities. Using multimodal monitoring, we identified the spring calling phenology of *P. maculata* in central Nebraska and found that vocalization activity was highly influenced by weather and habitat conditions.

P. maculata vocalization activity occurs predominately in the spring across the species' wide geographic extent, with precise timing of breeding activity dependent on latitude and elevation in addition to environmental conditions (Dodd 2013). Near the northern extent of *P.maculata*'s range in Quebec, Canada, and at higher elevations such as the Rocky Mountains, calling often begins in May and is associated with snowpack melt (Ouellet et al. 2009, Corn et al. 2000, Muths et al. 2016). In the southern extent of their range in New Mexico, breeding occurs from February to August (Degenhardt et al. 2005), and similar *Pseudacris* spp. have been reported to call as early as January and into March in Texas (Saenz et al. 2006). Prior studies utilizing trapping techniques in the Central Platte River Valley of Nebraska found *P. maculata* activity was highest in mid-April (Jones et al. 1981, Goldowitz and Whiles 1999), with calling observed as early as 18 March (Geluso and Harner 2013a). Although beneficial for specific questions, manual survey methods can have limitation for understanding phenological timing and associated drivers, and many surveys often did not begin until April (Jones et al. 1981, Goldowitz and Whiles 1999, Geluso and Harner 2013a). Furthermore, trapping surveys are likely

more reflective of movement than calling though both behaviors may be associated with reproduction (Spencer 1964, Kramer 1973). We observed *P. maculata* vocalization beginning in early March, often continuing into late May, and peaking in April. Differential activity patterns were observed between the wet meadow and forested slough, with *P. maculata* chorusing increasing at approximately day of year 70 (10 March) at the wet meadow and at day of year 100 (9 April) at the forested slough. This spring phenological pattern of *P. maculata* overlaps with the migratory timing of endangered whooping cranes in the CPRV, which on average occurs from late March through mid-April, with a mean initial Julian day of 92 (+/- 1; 2 April; Silcock and Jorgenson 2020). As whooping cranes stop to refuel and forage in sloughs and wet meadows along the Platte River, the overlapping phenology in conjunction with prior literature (Archibald and Meine, 1996, Bergeson et al. 2001, Geluso and Harner 2013b, Divets 2016) suggests that *P. maculata* and similar anuran species may be an available food resource for whooping cranes during their migration (Jones et al. 1981, Bergeson et al. 2001, Johnsgard 1983, Geluso et al. 2013). However, more research is needed, as little is known regarding whooping crane diet throughout most of the Great Plains (Caven et al. 2019b).

Acoustic metrics indicated *P. maculata* vocalizations at the wet meadow were much lower in 2015 than in the following two years. Upon examining spectrograms and listening to audio recordings, the chorusing of *P. maculata* was faint and sounded distant during spring of 2015, especially compared to 2016 and 2017, while other sounds, such as red-winged blackbirds (*Agelaius phoeniceus;* call frequencies of 1 – 5 kHz) and sandhill cranes (*Antigone canadensis;* call frequencies of 0.5 – 8 kHz), had similar sounding proximity and amplitude between years. Therefore, the observed decline seems explicit to *P. maculata*. Specific drivers of this decline are uncertain, albeit, we suggest potential influences were related to events that occurred prior to

spring 2015. In spring of 2013 and 2014, controlled burns were set to over 375 and 360 acres on Mormon Island, respectively, which contained much of the locally available wet meadow habitat. Although literature is sparse regarding responses of anurans to prescribed fire (Wilgers 2006, Harner and Geluso 2011), the burns may have had direct impacts on *P. maculata*, including mortality of adults, larva, or egg masses (Pilliod et al. 2003, Bowles and Jones 2013), or indirect impacts through alteration of habitat components, such as a rapid increase in water temperature, altered hydroperiod, changes in water chemistry, interstitial-filling of ash and sediments, or changes in vegetation density (Brown et al. 1990, Pilliod et al. 2003, Caldwell et al. 2013). As the area of wet meadow burned contains a high density of ephemeral and intermittent wetlands, and given that dispersal distances of *P. maculata* are limited and usually under 250 m (Spencer 1964, Werner et al. 2009, Scherer et al. 2012), it is likely that a high density of *P. maculata* were present in this area at the times of the fires. Prior studies report ambiguous findings, including declines in populations following fire (Driscoll and Roberts 1997), observations of anurans seeking refuge and fleeing from the sound of fire (Grafe et al. 2002), and species-specific responses to various burn regimes (Wilgers 2006). However, prescribed fire may have long-term benefits, as it increases habitat heterogeneity, nutrient availability, reduces woody encroachment, and maintains vegetation species richness over time (Pilliod et al. 2003, Bowles and Jones 2013).

Another possible explanation for the decline in activity was intensive grazing (> 300 cow-calf pairs) from mid-April 2014 to early October of the wet meadow, potentially influencing *P. maculata* activity indirectly through defoliation as *Pseudacris* species generally prefer habitats with significant vegetative cover (>75%) of at least moderate height (> 30 cm) (Anderson et al. 1999, Mannan 2008). Alternatively, *P. maculata* activity could have been reduced in 2015 as a

result of chytrid fungus (*Batrachochytrium dendrobatidis*). Harner et al. (2011, 2013) first documented the fungal disease in the CPRV at our study site in 2010 in American bullfrogs, then later in Woodhouse's toads and plains leopard frogs. Although chytrid fungus was not documented in boreal chorus frogs in the CPRV at the time, high rates (> 80%) of *P. maculata* mortality resulting from Chytridiomycosis have been documented elsewhere throughout its range (Hyman 2012). Mortality generally occurs 20 to 30 days after *B. dendrobatidis* exposure and higher water temperatures decrease infection and mortality rates (Hyman 2012, Amburgey 2014). As *P. maculata* reproduction occurs soon after spring emergence, many individuals likely may have had time to lay eggs ahead of potential adult mortality from Chytridiomycosis, and warming waters could have minimized potential impacts on larvae development (Hyman 2012). Therefore, as *P. maculata* takes 1-2 years to reach sexual maturity, high localized mortality in 2014 could have resulted in low localized calling during the following spring in 2015 (Hyman 2012, Amburgey 2014). Our data provides some support for this theory as there was a slight uptick in calling during May compared to March and April of 2015, aligned with the time the first tadpoles hatched in 2014 would reach sexual maturity and potentially become reproductively active (Amburgey 2014). While the cause is uncertain, the observed decrease in *P. maculata* vocalization emphasizes the importance of monitoring and the use of quantifiable measures, such as acoustic metrics, to identify disturbances which may be indicative of longer-term changes, management actions, or an abrupt shift in ecological systems.

Although we are unsure of the specific decrease in *P. maculata* calling observed in 2015, change in vegetation structure from fire or grazing may have influenced the species behavior. *P. maculata* use vegetation for various reasons; they will deposit egg masses beneath partially submerged or emergent vegetation, utilize branching vegetation for breeding displays, and

vegetation may provide structural cover and protection from predators (Anderson et al. 1999, Mannan 2008, Ballinger et al. 2010). *P. maculata* response to green chromatic coordinates, a vegetation index indicative of phenology, was predicted to peak at 0.35, which generally aligns with when vegetation begins to green-up, before declining steadily to minimal calling activity around 0.41, at which time vegetation would likely be dense and in full bloom, offering cover and protection for *P. maculata* post-breeding and natal dispersal into terrestrial environments (Wells 2010, Malzahn et al. *In Review*). Although random forest utilizes bagging and thus is known to be more robust in handling complex and interacting data (Breiman 2001), some studies suggest that there is potential for bias in the measures of importance for highly correlated variables (Strobl et al. 2008). Given the high correlation of vegetation and Julian day ($r = 0.8$), we recognize the limitations of this approach and acknowledge the possibility for inflating the importance of these variables. However, considering the iterations of randomness introduced within random forest models, examination of the partial dependence plots, and the *a priori* importance of both variables, we chose to include both vegetation and Julian day. As a proxy for seasonality, both vegetation and Julian day may offer useful information into the phenological pattern of *P. maculata* activity. Individually, they may provide different insight into potential long-term decoupling from climatic shifts, such as earlier breeding activity (Julian day) or potential resource mismatches (vegetation) (Klaus and Lougheed 2013, Visser and Gienapp 2019).

While there is a geographic difference in the timing of call activity based on latitude and elevation, *P. maculata* and similar *Pseudacris* species have been found to occupy a comparable temperature regime across their distribution. Our findings indicate *P. maculata* activity increased when average air temperature exceeded 5°C, an observation synonymous with prior studies

across the geographic range of *P. maculata*. Oullete et al. (2009) found *P. maculata* vocalizations began when temperatures were greater than 5°C in the Rocky Mountains, while Saenz et al. (2006) found *Pseudacris* species in Texas called at temperatures ranging from 5° C to 22°C (41°F to 72°F). Clarke 1958 reported *P. maculata* vocalized between air temperature ranges of approximately 4.5°C to 32°C (40°F to 90°F). In addition, we found a decline in calling activity when temperatures dropped below 5°C, such as in late March of 2016 and mid-April of 2017. Relative to other anuran species, *P. maculata* has been shown to breed earlier and in colder temperatures (Howard 1980). *Lithobates pipiens* (northern leopard frogs) were observed initiating calling 8-11 days after ice melt on an ephemeral breeding pond (Sommers et al. 2018). From the time-lapse images, we approximated an ice-off date of the wet meadow during our study and found that *P. maculata* on average started calling approximately 5 days after ice-melt. Similar to the snow-melt driven ponds in higher elevations (Corn 2003), snow and ice melt resulting from warming seasonal temperatures may act as an environmental cue (Corn and Muths 2002).

As *P. maculata* relies on ponded water for critical stages in their life cycle, including breeding, egg laying, and metamorphose, hydropattern is likely a key determinant of activity patterns (Saenz et al. 2006, Ballinger et al. 2010). Prior research shows *P. maculata* is adapted to local hydroperiod and lacks plasticity, suggesting their ability to respond to changes in hydropattern may be limited (Amburgey et al. 2012). Within the Central Platte River Valley, most wetlands are connected to the hydrology of the Platte River (Henszey and Wesche 1993), however, water and land use changes have greatly altered the remaining wetlands and their hydropattern (Sidle et al. 1989). Our results indicate hydropattern was an important variable at both the wet meadow and forested slough, with slight differences in the response of *P. maculata*

calling likely a factor of site-specific hydrological conditions. In mid-April 2016 as well as the end of April and beginning of May 2017, *P. maculata* calling decreased precipitously at both sites, concurrent with a decline in wet meadow inundation and decrease in Platte River streamflow, suggesting the decrease in *P. maculata* vocalization was likely attributed to water availability. Calling activity increased following much higher weekly rain totals at the forested slough (3.0-6.0 cm; Figure 4) than at the wet meadow (0.2-0.5 cm; Figure 4), where these large rain events most commonly occurred at the site in April when calling activity peaked (Figures 2, 3). It is possible that breeding activity also occurred in temporary pools outside of the forested slough following significant precipitation accumulation, explained by the high importance rank of 7-day precipitation at the forested slough but less important at the wet meadow. However, seven-day rainfall accumulation was an important predictor of *P. maculata* calling activity at both sites, indicating precipitation regimes sufficient to inundate temporary and intermittent wetlands likely act as an environmental cue for opportunistic *P. maculata* breeding activity (Saenz et al. 2006, Ballinger et al. 2010).

Hydropattern was an important variable at both sites, albeit, site specific differences in hydrology and resulting characteristics potentially influenced *P. maculata* calling at the wet meadow compared to the forested slough. Although calling activity generally peaked in April at both sites, peak activity was broadly more stochastic and frequent at the wet meadow, often with near-consistent vocalizations from mid-March declining into April followed by another peak into May. As groundwater, a major driver of wetland inundation (Wesche et al. 1994, Davis et al. 2006), is generally highest in March, high levels may have provided suitable inundation for breeding at the wet meadow site, but generally too much water for breeding at the forested slough, which consisted of topographically deeper marsh habitat (Kantrud et al. 1989, Whiles

and Goldowitz 1998, Goldowitz and Whiles 1999, Dodd et al. 2013). Prior research suggests *P. maculata* prefers intermittent wetlands, likely as an avoidance of potential desiccation from quick-drying shallow wetlands and to avoid predation from fish and other frog species within more permanent bodies of water (Kolozsvary and Swihart 1999, Dodd 2013). In the CPRV, *P. maculata* was found to be most abundant in wetlands with water present 60-90% of the time (Goldowitz and Whiles 1999), while they were not observed in perennial waters (Geluso and Harner 2013a). *P. maculata* calling activity was predicted to increase when water inundation was above 0.2 at the wet meadow, which remained inundated for most of the spring breeding season. At the forested slough, *P. maculata* activity was predicted to increase when streamflow reached 50 m^3/sec (~1766 ft^3/sec; Figure 4), and when streamflow exceeded this, *P. maculata* activity was predicted to decline. Our results suggest at the forested slough, streamflow above 50 m^3/sec may increase water depths in the wetland dominated by shallow and deep marsh habitats (Kantrud et al. 1989) above preferred levels for boreal chorus frogs.

The forested slough has a more permanent hydroperiod and often year-round water availability with close proximity to the Platte River, creating suitable habitat for *Lithobates catesbeianus* (American bullfrog) and posing a potential predation risk to native species through temporary colonization of fish species (Skelly 1996, Goldowitz and Whiles 1999, Knapp and Matthews 2000). Comparatively, the wet meadow exhibits more intermittent hydrology not conducive to bullfrogs. Bullfrog vocalizations were heard semi-regularly in audio recordings at the forested slough, potentially influencing *P. maculata* activity through resource competition, predation, and disease transmission such as the fungal pathogen *B. dendrobatidis* (Pearl et al. 2004, Harner et al. 2013). Introductions of *L. catesbeianus* to aquatic systems have shown that native species of frogs will change their calling behavior in response to overlapping vocalization

activity (Medeiros et al. 2016). At the forested slough, *P. maculata* vocalizations were relatively inactive during the day compared to the wet meadow, where we observed more consistent calling activity within a 24-hour period (Figure 2). Conversely, woodlands are not preferred habitat for *P. maculata*, which may provide further explanation for lower calling activity at the forested slough (Ballinger et al. 2010, Malzahn *In Review*).

5. Conclusions

Our findings indicate the spring calling activity of *P. maculata* follows a phenological pattern, initiating on average around day of year 70 and peaking from approximately day of year 100 to 120 in south-central Nebraska, USA. Furthermore, vocalization activity is associated with environmental variables and habitat condition, with predicted calling activity responding to temperatures above 5°C, weekly accumulated precipitation, and hydropattern. We detected greater vocalizations and near continuous diel calling activity in an intermittent wet meadow habitat compared to a forested slough with semi-permanent hydrology. These findings emphasize the importance of hydroperiod for the lifecycle of *P. maculata*. The small ephemeral wetlands preferred by *P. maculata* may be vulnerable to changes in land and water-use as cropland expands, water demands increase, and precipitation regimes change (Amburgey et al. 2012). As they rely on prairie-wetland ecotones, *P. maculata* may be a useful bioindicator reflecting environmental conditions across both grassland and aquatic habitats (Knutson et al. 2000). Autonomous sound recorders and acoustic metrics are particularly useful for assessing anurans, as they are often elusive, nocturnal, small, yet vocal (Bee et al. 2010, Ulloa et al. 2019, Sugai et al. 2019). Concurrently, time-lapse cameras offer an approach to examine visual changes in habitat and landscape (Sonnentag 2012, Brinley Buckley et al. 2017). Many challenges still exist

with implementing bioacoustics and multimodal monitoring, including processing time, learning curves, accuracy of classification, validation, loss of data due to passive technology malfunctions, integrating datasets, and handling, manipulating, and storing large amounts of data (Digby et al. 2013, Sugai et al. 2019). However, many of these limitations can be addressed with additional approaches such as manual listening and validation techniques. Additionally, multimodal monitoring can offer advantages for studying changing natural systems, including the ability to control frequency intervals and time duration, allowing for high frequency passive data collection over a long duration of time, the ability to have spatial replicates and collect data in remote or difficult to reach areas, minimal up-front human resources, and less time allocation than manual surveys or trapping. Declines in anuran populations have emphasized the need for increased monitoring to establish species-specific and geographically-relevant baseline references to characterize anuran phenology and activity patterns. More research is necessary to establish sound management and conservation practices, such as the effects of restoration activities that create permanent waterbodies in landscapes historically dominated by intermittent wetlands and anuran response to fire regimes and grazing. Integration of auditory and visual technologies into multimodal monitoring is a promising approach for understand species-specific responses to changing climatic and environmental conditions, altered habitat, and management practices.

6. Acknowledgements

This study was funded in part by the University of Nebraska Collaboration Initiative, the University of Nebraska at Kearney, the National Science Foundation under Grant No. DRL 1323615, and the United States Department of Agriculture under accession number 1016730. We thank Platte Basin Timelapse (PBT) for the use of imagery and The Crane Trust and Audubon's

Rowe Sanctuary for land access. We specifically acknowledge Michael Forsberg and Michael Farrell for their contributions to PBT, as well as Simon Tye for assisting with maintaining sound recorders and Carol Bedoya for assistance with sound analysis.

7. Literature Cited

Abrahams, C. and Geary, M., 2020. Combining bioacoustics and occupancy modelling for improved monitoring of rare breeding bird populations. Ecological Indicators, 112, p.106131.

Alexander, J.S., McElroy, B., Huzurbazar, S., Elliott, C., and Murr, M.L. 2020. Deposition potential and flow- response dynamics of emergent sandbars in a braided river. Water Resources Research, 56(1), e2018WR024107.

Amburgey, S., Funk, W.C., Murphy, M. and Muths, E., 2012. Effects of hydroperiod duration on survival, developmental rate, and size at metamorphosis in boreal chorus frog tadpoles (*Pseudacris maculata*). Herpetologica, 68(4), pp.456-467.

Amburgey, S. 2014. Species account for Boreal chorus frog (*Pseudacris maculata*). Colorado Partners in Amphibian and Reptile Conservation. http://www.coparc.org/colorado-amphibians---boreal-chorus-frog.html

Anderson, A.M., Haukos, D.A. and Anderson, J.T., 1999. Habitat use by anurans emerging and breeding in playa wetlands. Wildlife Society Bulletin, pp.759-769.

Archibald, G. W. and C. D. Meine. 1996. Family Gruidae (Cranes), p. 60-89. In.], del Hoyo, A. Elliott, J. Sargatal (eds.). Handbook of the birds of the world, Vol. 3: Hoatzin to Auks. Lynx Barcelona, Spain.

Bee, M.A., Cook, J.M., Love, E.K., O'Bryan, L.R., Pettitt, B.A., Schrode, K. and Vélez, A., 2010. Assessing acoustic signal variability and the potential for sexual selection and social recognition in boreal chorus frogs (*Pseudacris maculata*). Ethology, 116(6), pp.564-576.

Bergeson, D.G., Bradley, M. and Holroyd, G.L., 2001. Food items and feeding rates for wild whooping crane colts in Wood Buffalo National Park. North American Crane Workshop Proceedings. 46.

Both, C., Kaefer, I.L., Santos, T.G., and Cechin, S.T. 2008. An austral anuran assemblage in the Neotropics: seasonal occurrence correlated with photoperiod. Journal of Natural History, 42(3-4), pp 205-222.

Bowles, M.L., and Jones, M.D. 2013. Repeated burning of eastern tallgrass prairie increases richness and diversity, stabilizing late successional vegetation. Ecological Applications, 23(2), pp. 464-478.

Breiman, L., 2001. Random forests. Machine learning, 45(1), pp.5-32.

Bridges, A.S., and Dorcas, M.E. 2000. Temporal variation in anuran calling behavior: implications for surveys and monitoring programs. Copeia, 2000(2), pp 587-592.

Brinley Buckley, E.M, Allen, C.R., Forsberg, M., Farrell, M. and Caven, A.J., 2017. Capturing change: the duality of time-lapse imagery to acquire data and depict ecological dynamics. Ecology and Society, 22(3).

Brodie, S., Allen-Ankins, S., Towsey, M., Roe, P. and Schwarzkopf, L., 2020. Automated species identification of frog choruses in environmental recordings using acoustic indices. Ecological Indicators, 119, p.106852.

Brown, J.K. 1990. Effects of fire on aquatic systems. In Wild trout IV: proceedings of the symposium. US Government Printing Office, Washington, DC, USA, pp. 106-110.'

Browne, C.L., Paszkowski, C.A., Foote, A.L., Moenting, A., and Boss, S.M. 2009. The relationship of amphibian abundance to habitat features across spatial scales in the Boreal Plains. Ecoscience, 16(2), pp. 209-223.

Buxton, R.T., Lendrum, P.E., Crooks, K.R. and Wittemyer, G., 2018. Pairing camera traps and acoustic recorders to monitor the ecological impact of human disturbance. Global Ecology and Conservation, 16, p.e00493.

Caldwell, C.A., Jacobi, G.Z., Anderson, M.C., Parmenter, R.R., McGann, J., Gould, W.R., DuBey, R. and Jacobi, M.D. 2013. Prescribed-fire effects on an aquatic community of a southwest montane grassland system. North American Journal of Fisheries Management, 33(5), pp.1049-1062.

Caven, A.J., Buckley, E.M.B., King, K.C., Wiese, J.D., Baasch, D.M., Wright, G.D., Harner, M.J., Pearse, A.T., Rabbe, M., Varner, D.M. and Krohn, B. 2019a. Temporospatial shifts in Sandhill Crane staging in the Central Platte River Valley in response to climatic variation and habitat change. Monographs of the Western North American Naturalist, 11(1), pp.33-76.

Caven, A. J., Malzahn, J., Koupal, K. D., Buckley, E. M. B., Wiese, J. D., Rasmussen, R., and Steenson, C. 2019b. Adult Whooping Crane (*Grus americana*) consumption of juvenile channel catfish (*Ictalurus punctatus*) during the avian spring migration in the Central Platte River Valley, Nebraska, USA. Monographs of the Western North American Naturalist, 11(1), 14-23.

Caven, A. J., Varner, D. M., and Drahota, J. 2020. Sandhill Crane abundance in Nebraska during spring migration: making sense of multiple data points. Transactions of the Nebraska Academy of Sciences 40, pp 6–18

Center for Conservation Bioacoustics. 2019. Raven Pro: Interactive Sound Analysis Software (Version 1.6.1) [Computer software]. Ithaca, NY: The Cornell Lab of Ornithology. Available from http://ravensoundsoftware.com/.

Clarke, R.F., 1958. Ecological study of reptiles and amphibians in Osage County, Kansas. Emporia State Research Studies 7(1)

Conant, R. and J.T. Collins. 1991. A field guide to reptiles and amphibians: eastern and central North America. Houghton Mifflin Company. Boston, Massachusetts, USA.

Corn, P.S., Muths, E. and Iko, W.M., 2000. A comparison in Colorado of three methods to monitor breeding amphibians. Northwestern Naturalist, pp.22-30.

Corn, P. S., and Muths, E. 2002. Variable breeding phenology affects the exposure of amphibian embryos to ultraviolet radiation. Ecology, 83(11), pp. 2958-2963.

Corn, P.S., 2003. Amphibian breeding and climate change: importance of snow in the mountains. Conservation Biology, 17(2), pp.622-625.

Currier, P. J., and R. J. Henszey. 1996. Platte River Wet Meadows: A Primer on their Flora, Fauna, Hydrology, Management, and Restoration. Technical Report, US Fish and Wildlife Service (Project No. 14- 16- 0006- 90-917), Grand Island, NE.

Cutler, A., Cutler, D.R. and Stevens, J.R., 2012. Random forests. In Ensemble machine learning (pp. 157-175). Springer, Boston, MA.

Davis, C.A., J.E. Austin, and D.A. Buhl. 2006. Factors influencing soil invertebrate communities in riparian grasslands of the Central Platte River floodplain. Wetlands 26(2):438-454.

Degenhardt, W.G., Painter, C.W. and Price, A.H., 2005. Amphibians and reptiles of New Mexico. UNM Press.

Digby A, Towsey M, Bell BD, Teal PD. 2013. A practical comparison of manual and autonomous methods for acoustic monitoring. Methods in Ecology and Evolution 4: 675–683.

Dinets, V., 2016. Predation on amphibians and reptiles by reintroduced Whooping Cranes (*Grus americana*) in Louisiana. The American Midland Naturalist, 175(1), pp.134-137.

Dodd, C.K. 2013. Frogs of the United States and Canada. Johns Hopkins University Press, Baltimore, MD, USA. Vo1. 1 pp 371-384.

Driscoll, D.A. and Roberts, J.D., 1997. Impact of fuel- reduction burning on the frog *Geocrinia lutea* in southwest Western Australia. Australian Journal of Ecology, 22(3), pp.334-339.

Eschner, T.R., R.F. Hadley, and K.D. Crowley. 1983. Hydrologic and geomorphic studies of the Platte River Basin. U.S. Geological Survey Professional Paper 1277-A, United States Government Printing Office, Washington, DC. 297 ppFarina A, James P, Bobryk C, Pieretti N, Lattanzi E, McWilliam J. 2014. Low cost (audio) recording (LCR) for advancing soundscape ecology towards the conservation of sonic complexity and biodiversity in natural and urban landscapes. Urban Ecosystems 17: 923–944.

Fuhlendorf, S. D., Engle, D. M., Kerby, J. A. Y., and Hamilton, R. 2009. Pyric herbivory: rewilding landscapes through the recoupling of fire and grazing. Conservation Biology, 23(3), pp 588-598.

Fogell, D.D., 2010. Field guide to the amphibians and reptiles of Nebraska. University of Nebraska-Lincoln.

Gasc A, Francomano D, Dunning JB, Pijanowski BC. 2017. Future directions for soundscape ecology: The importance of ornithological contributions. Auk 134: 215–228

Geluso, K., Krohn, B.T., Harner, M.J. and Assenmacher, M.J., 2013. Whooping cranes consume plains leopard frogs at migratory stopover sites in Nebraska. Prairie Naturalist, 45, pp.91-93.

Geluso, K. and Harner, M.J., 2013a. Reexamination of Herpetofauna on Mormon Island, Hall County, Nebraska, with Notes on Natural History. Transactions of the Nebraska Academy of Sciences & Affiliated Societies, 33.

Geluso, K., and M.J. Harner. 2013b. Nerodia clarkii clarkii (Gulf Saltmarsh Watersnake): Predation. Herpetological Review 44:156-157.

Goldowitz, B.S. and Whiles, M.R., 1999. Investigations of Fish, Amphibians and Aquatic Invertebrate Species Within the Middle Platte River System. Final Report, Platte Watershed Program Cooperative Agreement X99708101. USEPA region VII, Kansas City, Missouri, USA.

Grafe, T.U., Doebler, S. and Linsenmair, K.E., 2002. Frogs flee from the sound of fire. Proceedings of the Royal Society of London. Series B: Biological Sciences, 269(1495), pp.999-1003.

Grant, E.H.C., Miller, D.A., Schmidt, B.R., Adams, M.J., Amburgey, S.M., Chambert, T., Cruickshank, S.S., Fisher, R.N., Green, D.M., Hossack, B.R. and Johnson, P.T., 2016. Quantitative evidence for the effects of multiple drivers on continental-scale amphibian declines. Scientific reports, 6(1), pp.1-9.

Grant, T.J., Otis, D.L. and Koford, R.R., 2015. Short- term anuran community dynamics in the Missouri River floodplain following an historic flood. Ecosphere, 6(10), pp.1-16.

Hafner, S, and Katz, J. 2018. monitoR: Acoustic template detection in R. R package version 1.0.7. < http://www.uvm.edu/rsenr/vtcfwru/R/?Page=monitoR/monitoR.htm >

Harner, M.J. and K. Geluso. 2011. Response of herpetofauna to grazing and fire in wet, tallgrass prairies along the Platte River. Nebraska State Wildlife Grant Final Report submitted to the Nebraska Game and Parks Commission, Lincoln, Nebraska, USA. 58 p.

Harner, M.J., Merlino, J.N. and Wright, G.D., 2013. Amphibian chytrid fungus in Woodhouse's toads, plains leopard frogs, and American bullfrogs along the Platte River, Nebraska, USA. Herpetological Review, 44(3), pp.459-461.

Henszey, R.J. and Wesche, T.A., 1993. Hydrologic Components Influencing the Condition of Wet Meadows Along the Central Platte River, Nebraska. Rep. Neb. Game Park. Comm.

Hillman, S.S., Withers PC, Drewes RC, Hillyard SD. 2009. Ecological and environmental physiology of amphibians. New York, NY: Oxford University Press.

Horn, J.D., Joeckel, R.M., and Fielding, C.R. 2012. Progressive abandonment and planform changes of the central Platte River in Nebraska, central USA, over historical timeframes. Geomorphology, 139, pp. 372-383.

Howard, R.D., 1980. Mating behavior and mating success in wood frogs, *Rana sylvatica*. Anim Behav 28:705–716

Hyman, O. 2012. Ecology of Chytridiomycosis in Boreal chorus frogs (*Pseudacris maculata*). Dissertation, Arizona State University, Tempe, AZ, USA.

Johansson, J., Kristensen, N.P., Nilsson, J.Å. and Jonzén, N. 2015. The eco- evolutionary consequences of interspecific phenological asynchrony–a theoretical perspective. Oikos, 124:102-112.

Johnsgard, P. A. 1983. Cranes of the world. Indiana University Press, Bloomington, Indiana

Jones, S.M., Ballinger, R.E. and Nietfeldt, J.W., 1981. Herpetofauna of Mormon Island Preserve, Hall County, Nebraska. The Prairie Naturalist, 13(2), pp.33-41.

Kantrud, H.A., Krapu, G.L. and Swanson, G.A., 1989. Prairie basin wetlands of the Dakotas: a community profile (Vol. 85, No. 7). US Department of the Interior, Fish and Wildlife Service, Research and Development.

Knapp, R.A., and K.R. Matthews. 2000. Non-native fish introductions and the decline of the mountain yellowlegged frog from within protected areas. Conservation Biology 14: 428–438.

Kaul, R.B., Sutherland, D., Rolfsmeier, S. 2011. The flora of Nebraska. School of Natural Resources, University of Nebraska-Lincoln, NE, USA.

Klaus, S.P. and Lougheed, S.C., 2013. Changes in breeding phenology of eastern Ontario frogs over four decades. Ecology and evolution, 3(4), pp.835-845.

Knutson, M.G., Sauer, J.R., Olsen, D.A., Mossman, M.J., Hemesath, L.M. and Lannoo, M.J., 2000. Landscape associations of frog and toad species in Iowa and Wisconsin, USA. Journal of the Iowa Academy of Science: JIAS, 107(3-4), pp.134-145.

Kolozsvary, M.B. and Swihart, R.K., 1999. Habitat fragmentation and the distribution of amphibians: patch and landscape correlates in farmland. Canadian Journal of Zoology, 77(8), pp.1288-1299.

Kramer, D. C., 1973. Movements of western chorus frogs *Pseudacris triseriata triseriata* tagged with Co 60. Journal of Herpetology, 7, pp. 231-235.

LaGrange, T.G. 2005. A Guide to Nebraska's Wetlands and their Conservation Needs. Nebraska Game and Parks Commission, Lincoln, USA.

Liaw, A. and Wiener, M., 2002. Classification and regression by randomForest. R news, 2(3), pp.18-22.

Lingle, G.R., G.A. Wingfield, and J.W. Ziewitz. 1991. The migration ecology of Whooping Cranes in Nebraska, USA. Pages 395–401 in J. Harris, editor, Proceedings of the 1987 International Crane Workshop. International Crane Foundation, Baraboo, WI.

Lofts, B. 1974. Reproduction. In: Lofts B (ed) Physiology of the amphibia, vol II. Academic Press, New York, pp 107–218

Machado, R.B., Aguiar, L., and Jones, G. 2017. Do acoustic indices reflect the characteristics of bird communities in the savannas of Central Brazil?. Landscape and Urban Planning, 162,pp 36-43.

Madalozzo, B., Santos, T.G., Santos, M.B., Both, C., and Cechin, S. 2017. Biodiversity assessment: selecting sampling techniques to access anuran diversity in grassland ecosystems. Wildlife Research, 44(1), pp 78-91.

Mannan, R.N., 2008. An assessment of survey methodology, calling activity, and habitat associations of Wood Frogs (*Rana sylvatica*) and Boreal chorus frogs (*Pseudacris maculata*) in a tundra biome. Doctoral dissertation, Texas Tech University, Lubbock, Texas, USA.

Malzahn, J., A.J. Caven, S. Warren, B. Ostrom, and D. Ferraro. *In Review*. Habitat associations of various herpetofauna in the Central Platte River Valley, Nebraska, with notes on morphometric characteristics. Platte River Whooping Crane Maintenance Trust, Inc., Wood River, NE, USA, pp. 1-40.

Matthews, J.H., Funk, W.C. and Ghalambor, C.K., 2013. Demographic approaches to assessing climate change impact: an application to pond-breeding frogs and shifting hydropatterns. Wildlife conservation in a changing climate, pp.58-85.

Menne, M.J., I. Durre, B. Korzeniewski, S. McNeal, K. Thomas, X. Yin, S. Anthony, R. Ray, R.S. Vose, B.E.Gleason, and T.G. Houston, 2012. Global Historical Climatology Network - Daily (GHCN-Daily), Version 3.26 NOAA National Centers for Environmental Information http://doi.org/10.7289/V5D21VHZ [Accessed 18 April 2019].

Meyer, C.K., and M.R. Whiles. 2008. Macroinvertebrate communities in restored and natural Platte River slough wetlands. Journal of the North American Benthological Society 27:626-639.

Mushet, D.M, Euliss Jr, N.H, and Stockwell, C.A. 2012. Mapping Anuran Habitat Suitability to Estimate Effects of Grassland and Wetland Conservation Programs. Copeia, 2012(2), pp. 321-330.

Muths, E., Scherer, R.D., Amburgey, S.M., Matthews, T., Spencer, A.W. and Corn, P.S., 2016. First estimates of the probability of survival in a small-bodied, high-elevation frog (Boreal chorus frog, *Pseudacris maculata*), or how historical data can be useful. Canadian Journal of Zoology, 94(9), pp.599-606.

Nelson, G.L., and Graves, B.M. 2004. Anuran population monitoring: comparison of the North American Amphibian Monitoring Program's calling index with mark-recapture estimates for *Rana clamitans*. Journal of Herpetology, 38(3), pp. 355-359.

Oseen, K.L. and Wassersug, R.J., 2002. Environmental factors influencing calling in sympatric anurans. Oecologia, 133(4), pp.616-625.

Ospina, O.E., Villanueva-Rivera, L.J., Corrada-Bravo, C. and Aide, T.M., 2013. Variable response of anuran calling activity to daily precipitation and temperature: implications for climate change. Ecosphere, 4(4), pp.1-12.

Ouellet , M., Fortin, C. and Grimard, M.J., 2009. Distribution and habitat use of the Boreal chorus frog (Pseudacris maculata) at its extreme northeastern range limit. Herpetological Conservation and Biology, 4(2), pp.277-284.

Pearl, C.A., Adams, M.J., Bury, R.B. and McCreary, B., 2004. Asymmetrical effects of introduced bullfrogs (*Rana catesbeiana*) on native ranid frogs in Oregon. Copeia, 2004(1), pp.11-20.

Pearse, A. T., Krapu, G. L., Cox, R. R., and Davis, B. E. 2011. Spring-migration ecology of Northern Pintails in south-central Nebraska. Waterbirds, 34(1), pp 10-18.

Pekel, J.F., Vancutsem, C., Bastin, L., Clerici, M., Vanbogaert, E., Bartholomé, E. and Defourny, P., 2014. A near real-time water surface detection method based on HSV transformation of MODIS multi-spectral time series data. Remote sensing of environment, 140, pp.704-716.

Pijanowski, B.C., Villanueva-Rivera, L.J., Dumyahn, S.L., Farina, A., Krause, B.L., Napoletano, B.M., Gage, S.H. and Pieretti, N., 2011. Soundscape ecology: the science of sound in the landscape. BioScience, 61(3), pp.203-216.

Pilliod, D.S., Bury, R.B., Hyde, E.J., Pearl, C.A. and Corn, P.S., 2003. Fire and amphibians in North America. Forest ecology and management, 178(1-2), pp.163-181.

R Core Team. 2020. R: A language and environment for statistical computing. R Foundation for Statistical Computing, Vienna, Austria. URL https://www.R-project.org/.

Reichert, B., and Lausen, C., Loeb, S., Weller, T., Allen, R., Britzke, E., Hohoff, T., Siemers, J., Burkholder, B., Herzog, C., and Verant, M., 2018, A guide to processing bat acoustic data for the North American Bat Monitoring Program (NABat): U.S. Geological Survey Open-File Report 2018–1068, 33 p., https://doi.org/10.3133/ofr20181068.

Richardson, A.D., Hufkens, K., Milliman, T., Aubrecht, D.M., Chen, M., Gray, J.M., Johnston, M.R., Keenan, T.F., Klosterman, S.T., Kosmala, M. and Melaas, E.K., 2018. Tracking vegetation phenology across diverse North American biomes using PhenoCam imagery. Scientific data, 5, p.180028.

Ryan, T.J. and Winne, C.T., 2001. Effects of hydroperiod on metamorphosis in *Rana sphenocephala*. The American Midland Naturalist, 145(1), pp.46-53.

Saenz, D., Fitzgerald, L.A., Baum, K.A. and Conner, R.N., 2006. Abiotic correlates of anuran calling phenology: the importance of rain, temperature, and season. Herpetological Monographs, 20(1), pp.64-82.

Scherer, R.D., Muths, E. and Noon, B.R., 2012. The importance of local and landscape-scale processes to the occupancy of wetlands by pond-breeding amphibians. Population Ecology, 54(4), pp.487-498.

Schindelin, J., Arganda-Carreras, I., Frise, E., Kaynig, V., Longair, M., Pietzsch, T., Preibisch, S., Rueden, C., Saalfeld, S., Schmid, B. and Tinevez, J.Y., 2012. Fiji: an open-source platform for biological-image analysis. Nature methods, 9(7), pp.676-682.

Schneider, D.C. 2009. Quantitative ecology: measurement, models and scaling. Academic press, Elsevier, London, UK.

Seburn, D.C., Gunson, K. and Schueler, F.W., 2014. Apparent widespread decline of the Boreal chorus frog (*Pseudacris maculata*) in eastern Ottawa. The Canadian Field-Naturalist, 128(2), pp.151-157.

Sidle, J.G., E.D. Miller, and P.J. Currier. 1989. Changing habitats in the Platte River valley of Nebraska. Prairie Naturalist 21:91–104.

Silcock, W.R., and J.G. Jorgensen. 2020. Whooping Crane (*Grus americana*), Version 1.0. In Birds of Nebraska — Online**.** www.BirdsofNebraska.org

Skelly, D.K. 1996. Pond drying, predators, and the distribution of *Pseudacris* tadpoles. Copeia 1996(3), pp. 599–605.

Smith, N.D. 1971. Transverse bars and braiding in the lower Platte River, Nebraska. Geological Society of America Bulletin, 82(12), pp.3407-3420.

Sodhi, N.S., Bickford, D., Diesmos, A.C., Lee, T.M., Koh, L.P., Brook, B.W., Sekercioglu, C.H. and Bradshaw, C.J., 2008. Measuring the meltdown: drivers of global amphibian extinction and decline. PloS one, 3(2), p.e1636.

Sommers, M.D., Randall, L.A. and Barclay, R.M.R., 2018. Effects of environmental variables on the calling behaviour of Northern Leopard Frogs (*Lithobates pipiens*) in Alberta, Canada. Canadian Journal of Zoology, 96(2), pp.163-169.

Sonnentag, O., Hufkens, K., Teshera-Sterne, C., Young, A.M., Friedl, M., Braswell, B.H., Milliman, T., O'Keefe, J. and Richardson, A.D., 2012. Digital repeat photography for phenological research in forest ecosystems. Agricultural and Forest Meteorology, 152, pp.159-177.

Spencer A.W. 1964. The Relationship of Dispersal and Migration to Gene Flow in the Boreal chorus frog. Ph.D. dissertation, Colorado State University, Fort Collins, CO, USA.

Steelman, C.K. and Dorcas, M.E., 2010. Anuran calling survey optimization: developing and testing predictive models of anuran calling activity. Journal of Herpetology, 44(1), pp.61-68.

Stevens, C.E., Diamond, A.W., & Gabor, T.S. 2002. Anuran call surveys on small wetlands in Prince Edward Island, Canada restored by dredging of sediments. Wetlands, 22(1), pp. 90-99.

Strobl, C., Boulesteix, A.L., Kneib, T., Augustin, T. and Zeileis, A., 2008. Conditional variable importance for random forests. BMC bioinformatics, 9(1), p.307.

Sueur, J. and Farina, A., 2015. Ecoacoustics: the ecological investigation and interpretation of environmental sound. Biosemiotics, 8(3), pp.493-502.

Sugai, L.S.M., Silva, T.S.F., Ribeiro Jr, J.W. and Llusia, D., 2019. Terrestrial passive acoustic monitoring: review and perspectives. BioScience, 69(1), pp.15-25.

Todd, M.J., Cocklin, R.R., and Dorcas, M.E. 2003. Temporal and spatial variation in anuran calling activity in the western Piedmont of North Carolina. Journal of the North Carolina Academy of Science, 119(3), pp 103-110.

Visser, M.E. and Gienapp, P., 2019. Evolutionary and demographic consequences of phenological mismatches. Nature Ecology & Evolution, 3(6), pp.879-885.

Vrtiska, M. P., and Sullivan, S. 2009. Abundance and distribution of lesser snow and Ross's geese in the Rainwater Basin and central Platte River Valley of Nebraska. Great Plains Research, 147-155.

Weir, L.A. and Mossman, M.J., 2005. North American Amphibian Monitoring Program (NAAMP). In M. J. Lannoo (ed.), Amphibian Declines: Conservation Status of United States Species, pp. 307-313. University of California Press, Berkeley, California, USA

Werner, E.E., Relyea, R.A., Yurewicz, K.L., Skelly, D.K. and Davis, C.J., 2009. Comparative landscape dynamics of two anuran species: climate‐ driven interaction of local and regional processes. Ecological Monographs, 79(3), pp.503-521.

Wesche, T.A., O.D. Skinner, and R.J. Henszey. 1994. Platte River wetland hydrology study. Final report prepared for U.S. Bureau of Reclamation by Wyoming Water Resources Center, University of Wyoming, Laramie, Wyoming, 165 p.

Westgate, M.J., MacGregor, C., Scheele, B.C., Driscoll, D.A. and Lindenmayer, D.B., 2018. Effects of time since fire on frog occurrence are altered by isolation, vegetation and fire frequency gradients. Diversity and Distributions, 24(1), pp.82-91.

Whiles, M.R., and B.S. Goldowitz. 1998. Biological responses to hydrologic fluctuation in wetland sloughs of the Central Platte River. Proceedings of the Platte River Basin Ecosystem Symposium 9:3-18.

Whiles, M.R., and B.S. Goldowitz. 2005. Macroinvertebrate communities in Central Platte River wetlands: patterns across a hydrologic gradient. Wetlands 25:462-472.

Whitby, M.D., Carter, T.C., Britzke, E.R. and Bergeson, S.M., 2014. Evaluation of mobile acoustic techniques for bat population monitoring. Acta Chiropterologica, 16(1), pp.223-230.

Wilgers, D.J. and Horne, E.A., 2006. Effects of different burn regimes on tallgrass prairie herpetofaunal species diversity and community composition in the Flint Hills, Kansas. Journal of herpetology, 40(1), pp.73-84.

Williams, G. P. 1978. The Case of the Shrinking Channels: The North Platte and Platte Rivers in Nebraska. Circulars of the United States Geological Survey. No. 781

*Manuscript (revision changes marked)
Click here to download Manuscript (revision changes marked): BrinleyBuckley_etal_2020_[illegible]

Assessing ecological and environmental influences on ~~Boreal Chorus Frog~~boreal chorus frog (*Pseudacris maculata*) spring calling phenology using multimodal passive monitoring technologies

Emma M. Brinley Buckley[1*], Benjamin L. Gottesman[2], Andrew J. Caven[3], Mary J. Harner[1,4], Bryan C. Pijanowski[2]

[*]Corresponding Author, brinleybuckleyem@unk.edu
[1]Department of Communication, University of Nebraska at Kearney, Kearney, NE 68849
[2]Center for Global Soundscapes and Department of Forestry & Natural Resources, Purdue University, West Lafayette, IN 47907
[3]Platte River Whooping Crane Maintenance Trust, Wood River, Nebraska 68883
[4]Department of Biology, University of Nebraska at Kearney, Kearney, NE 68849

Abstract

Although anuran reproductive behavior follows a phenological pattern, environmental factors may influence the timing of anuran activity. To characterize the spring calling phenology of *Pseudacris maculata* (~~Boreal chorus frog~~boreal chorus frog) we installed ~~passive~~ acoustic recorders during three spring breeding seasons (2015-2017) within wet meadow and forested slough habitats in central Nebraska near the Platte River. The recorders were paired with time-lapse camera systems that were used to measure vegetation change and wet meadow inundation. We examined the relationship between environmental variables, including temperature, precipitation, and hydropattern, and *P. maculata* calling activity using r~~R~~andom f~~F~~orest models. Calling activity exhibited diel and seasonal variation with differences observed between habitats. Hourly call activity was highest between 20:00 and 01:00, with diurnal ~~calling~~ and sometimes near-continuous calling observed at the wet meadow but not at the forested slough. Random forest~~F~~ models explained 73% of calling activity variance at the wet meadow and 45% at the forested slough, suggesting additional factors, such as predation, may be influential at the forested slough, as it exhibited near permanent hydrology. *P. maculata* was predicted to increase chorusing at the 70th day of the year at the wet meadow but not until the 100th at the forested slough. When average air temperatures exceeded 5°C, calling activity was predicted to increase

at both wetlands, and calling declined below this temperature. Hydropattern and weekly precipitation accumulation were important predictors of *P. maculata* calling activity. Our findings highlight the importance of climatic factors such as temperature and precipitation regimes, in addition to habitat conditions such as water-availability, on *P. maculata* calling activity. Furthermore, this study demonstrates the utility of multimodal passive monitoring technologies, including both visual and auditory tools, for understanding the environmental factors that influence biological activity. Multimodal approaches can be especially useful for documenting and measuring vocal-taxa ~~bio~~diversity, activity, and drivers of change.

Keywords: *Pseudacris maculata*, passive acoustic monitoring, wetlands, Central Platte River, random forest model, image analysis, wet meadows, sloughs

1. Introduction

The relationship between animals and their environment is synchronously intertwined, as biotic and abiotic conditions influence the timing of important animal behaviors (Johansson et al. 2015). In temperate regions, amphibians such as anurans have adapted life history strategies aligned with local climatic and physical conditions (Lofts 1974, Amburgey et al. 2012). Over the last four decades, anuran populations have declined globally due to compounding factors including changes in land and water use, habitat loss, pathogens, and a changing climate (Sodhi et al. 2008, Grant et al. 2016, Green et al. 2020). In the Great Plains, amphibians are declining by an estimated >3% annually, however, information on anuran phenology and environmental drivers of anuran behavior in the region is sparse (Mushet et al. 2012, Grant et al. 2016). Often anuran research, including trapping, manual listening, and visual encounter surveys, is conducted

for short periods at infrequent intervals (Bridges and Dorcas 2000). For elusive yet vocal animals, such as small and mostly nocturnal anurans, auditory surveys have proven beneficial for monitoring, for example the North American Amphibian Monitoring Program (Weir and Mossman 2005). As their main form of communication, anurans vocalize predominately to attract mates and alert rival males to their presence (Bee et al. 2010). Manual listening surveys of anuran calling frequency, intensity, and number (usually in the form of scaled indices) can be used to model species behavioral patterns, spatial distribution, temporal occurrence, and responses to environmental change (Stevens et al. 2002, Nelson et al. 2004, Both et al. 2008). Although these methods can provide important information about long term trends and change, the data is often temporally limited, which can be problematic considering the high variability in anuran calling activity (Bridges and Dorcas 2000, Todd et al. 2003). Passive acoustic recording, which is increasingly used to monitor and assess changes in soundscapes (Pijanowski et al. 2011), ecosystem state (Sueur and Farina 2015), and vocalizing taxa including bird communities ~~populations~~ (Gasc et al. 2017, Abrahams and Geary 2020) and bat activity (Whitby et al. 2014, Reichert, et al. 2018), may offer an alternative with higher temporal resolution at hourly or daily intervals (Digby et al. 2013, Sugai et al. 2019). Moreover, this technology remains comparatively underutilized for anurans considering its potential benefits (Bee et al. 2010, Madalozzo et al. 2017, Brodie et al. 2020).

Anuran calling activity exhibits a phenological pattern, occurring during the breeding season but influenced by biotic and abiotic factors, including habitat, vegetation, hydrology, food availability, and climatic factors ~~climatic and habitat conditions~~ (Oseen and Wasserug 2002, Klaus and Lougheed 2013, Ospina et al. 2013). Changes ~~in these factors~~in climatic and habitat conditions, such as an altered hydrologic regime or vegetation phenophase,- can result in

phenological shifts of behavior, such as reproduction ~~ve behavior~~, leading to potential resource mismatches (Klaus and Lougheed 2013). Studies have consistently found that temperature influences the lifecycle and activity of ectothermic anurans, however even similar species have shown differential responses to temperature regimes (Oseen and Wassersug 2002, Hillman et al. 2009, Steelman and Dorcas 2010). Many anuran species are dependent upon aquatic-terrestrial ecotones, utilizing terrestrial habitat during their adult phase and aquatic habitat during reproduction and metamorphose, and therefore, water availability generally plays an important role in reproductive behavior (Ryan and Winne 2001, Amburgey et al. 2012). However, the magnitude and mechanism of influence, such as precipitation or hydropattern (the hydroperiod, hydrologic fluctuation, timing, and flow regime of a system; Mathews et al. 2010, Amburgey et al. 2014), likely differs between ephemeral and permanent or semi-permanent wetlands, as well as geographic regions, and by species (Werner et al. 2009, Ospina et al. 2013). In addition to the hydropattern of a system, habitat conditions such as vegetation structure may influence calling ~~activity~~, as many anurans, including *Pseudacris* ~~*maculata*~~*maculate* (~~, the Boreal~~ boreal c~~C~~horus f~~F~~rog), depend on plants for protection from predators, cling to vegetation during chorusing, as well as lay egg masses beneath vegetation (Conant and Collins 1991, Andersen et al. 1999, Grant et al. 2019).

P. maculata, a small (~3.3 cm) trilling chorus frog, is found in emergent wetlands and grasslands across central parts of the United States and Canada, with statewide distribution in Nebraska (Fogell 2010). Populations of *P. maculata* are broadly considered stable, however, declines in *Pseudacris* species (*sensu lato*, trilling chorus frogs) have been observed in several regions, including the Great Plains, Midwest, eastern Ontario, western Quebec, as well as possible extirpation from New York and Vermont (Knutson et al. 2000, Seburn et al. 2014).

Studies show that *P. maculata* are exposed to a myriad of threats, including human influences, disease, toxins, and climatic factors (Grant et al. 2016), and populations are impacted by drought and flooding (Werner et al. 2009, Grant et al. 2019). In the Great Plains, *P. maculata* chorusing is often one of the earliest vocalizations heard and an iconic sign of spring, with breeding taking place as early as March and continuing as late as May with occasional breeding activity into the summer following large precipitation events (Jones et al. 1981, Fogell 2010). As *P. maculata* exhibits an early spring phenological pattern, likely driven by weather and habitat conditions, pairing auditory and visual autonomous technologies can provide documentation of local biotic and environmental changes at various scales, which may help to disentangle complex ecological interactions (Buxton et al. 2018).

In this study, we aimed to assess how *P. maculata* calling activity related to local environmental conditions in central Nebraska during three spring breeding seasons (2015-2017) using multimodal passive monitoring technologies. We used aural surveys, acoustic metrics, and automated classification of sound recordings~~ers~~ ~~and acoustic analysis~~ to examine hourly and daily call activity patterns and characterize variability in *P. maculata* spring calling phenology. Image analysis was used to quantify changes in hydroperiod and vegetation. We assessed the importance of hydropattern (inundation and river streamflow), vegetation, temperature, wind speed, precipitation, and snowfall on *P. maculata* activity using random forest models. We examined the call activity of *P. maculata* within wet meadow habitat compared to a semi-permanent forested wetland. Combining acoustic and visual monitoring technologies, our research attempted to gain insight into the phenological patterns of *P. maculata* spring calling activity and to identify important environmental factors influencing activity.

2. Methods

2.1. Study Area

The study was conducted in the Central Platte River Valley (CPRV) of Nebraska, an important avian stopover location within the Central Flyway migration corridor (Vrtiska and Sullivan 2009, Pearse et al. 2011, Caven et al. 2019a). The region is used by endangered w~~W~~hooping c~~C~~ranes, hundreds of thousands of s~~S~~andhill cranes, and millions of other waterbirds as a stopover to rest and refuel by foraging on vegetation, fish, insects, and anurans (Lingle et al. 1991, LaGrange 2005, Vrtiska and Sullivan 2009, Geluso et al. 2013, Caven et al. 2019b, Caven et al. 2020). The Platte River extends the reach of tallgrass prairie habitat west into the mixed-grass ecoregion in central Nebraska as a result of subirrigation, and relatively minor elevational changes within this riverine ecosystem provide for a dynamic set of wetland habitats with varying hydroperiods (Currier 1995, Whiles and Goldowitz 2005, Kaul et al. 2011, Meyer and Whiles 2010). Wet meadows represent more ephemeral wetlands with shorter hydroperiods that usually experience inundation through the early spring, while shallow and deep marsh habitats experience increasing hydroperiods lasting into the summer or even throughout the growing season in some years (Kantrud et al. 1989, Currier 1995, Whiles and Goldowitz 2005, Tiner 2016). These wetlands are connected to the hydrology of the Platte River, which experiences high flows in the spring from snowmelt with declining streamflow into the summer (Williams 1978, Eschner et al. 1983, Currier and Henszey 1996). Historically, the Platte River was a wide braided river that sustained a large number of riparian wetlands within a grassland mosaic (Smith 1971, Williams 1978, Currier and Henszey 1996). However, over the last century, extensive land and water use changes have altered the landscape, negatively impacting river hydrogeomorphology, ~~wildlife, and~~ wetland hydrologic connectivity, and wildlife (Sidle et al. 1989, Eschner et al. 1983, Horn et al. 2012, Alexander et al. 2020). Conversion of grassland and

wetland habitats to agriculture and diversions for irrigation has removed or retimed the majority of streamflow in the Platte River and erased over 70% of wet meadows and other wetlands from the CPRV (Williams 1978, Eschner et al. 1983, Sidle et al. 1989, Currier and Henszey 1996). Understanding how hydrologic and environmental fluctuations influence b~~B~~oreal c~~C~~horus f~~F~~rog activity in the remaining high-quality wetlands should further inform habitat conservation and restoration efforts focused on preserving this species.

2.2. Environmental Variables

Weather data was acquired from the National Oceanic and Atmospheric Administration, ~~'s~~ National Centers for Environmental Information, Global Historical Climatology Network–Daily database (Menne et al. 2012). We ~~,~~obtained~~ing~~ data from Kearney, NE (station USC00254335) for the forested slough and Grand Island, NE (station USW00014935) for the wet meadow. Daily variables included average wind speed, total precipitation, mean, maximum, and minimum air temperature, snow accumulation and snow depth. Average wind speed was only available at the Grand Island weather station and was therefore used for both study sites. Hydropattern ~~for~~relating to the forested slough was obtained from a USGS stream gage for the Platte River at Kearney, Nebraska (USGS 06770200).

We derived vegetation phenology and water inundation data from analysis of time-lapse images from Mormon Island and Rowe Sanctuary Tower cameras of the Platte Basin Timelapse project~~.~~, a long-term monitoring and science communication project documenting the Platte Basin (www.plattebasintimelapse.com). The time-lapse camera systems, consisting of a Nikon D300 DSLR with a 12.3 megapixel crop sensor and Nikkor 18-70mm 1:3.5-4.5 lens set at F/8 and powered by a solar panel, took one image every hour of sunlight. Images were processed by selecting daily images taken between 10:00 and 14:00 to standardize for sun exposure and shadows. We then visually inspected the imagery collections, and images with obstructed views

or inconsistencies, such as fog or spider webs, were removed from the analysis. Based on the digital number (DN) within the red-green-blue (RGB) colorspace of a pixel, we calculated green chromatic coordinates (GCC), a greenness index of vegetation phenology (Sonnentag 2012). At each site, we selected a region of interest (ROI) that encompassed vegetation and excluded sky and other features within the frame of view. GCC was calculated for each site's ROI using the equation GCC = G/[R + G + B] (Richardson 2018).

For the wet meadow at Mormon Island, we assessed water inundation through image-analysis using a macro script adapted from microscopy in the Java-based open-access program FIJI (Schindelin et al. 2012, Brinley Buckley et al. 2017). A ROI, separate from the ROI used to calculate GCC, was selected that excluded sky, horizon, and additional grassland landscape to reduce variability. The RGB colorspace was transformed to hue-saturation-value (HSV) to overcome potential limitations of the RGB color space for classifying water (Pekel et al. 2014). The ROI was converted to binary (0,1) by automated thresholding (threshold = (average background + average objects)/2). The binary masked results were visually inspected and manually compared to the original image for accuracy, and if a mask was visibly inaccurate, the original image was classified using heads up digitizing by manually tracing water inundation. A proxy for ponding surface water, the resulting water inundation (WI) values were the percent area classified as water within the region of interest. The final dataset was averaged into daily values and rescaled to a 0 to 1 value.

Formatted: Indent: First line: 0 cm

2.3. Acoustic Analyses

We installed two acoustic monitoring stationsdigital autonomous acoustic recorders paired with time-lapse camera systems within the CPRV from 1 March 2015 to 1 June 2017. The

~~time-lapse cameras were installed prior to the acoustic recorders as part of the Platte Basin Timelapse Project, a long-term monitoring and science communication project documenting the Platte Basin (www.plattebasintimelapse.com).~~ Recorders were deployed next to the cameras approximately 1.5 m above the ground. The first paired monitoring system was within Mormon Island, a wet meadow habitat between two channels of the Platte River in southcentral Nebraska (40.799, -98.417). The site is generally devoid of trees and dominated by sedges (*Carex* spp.), common threesquare (*Schoenoplectus pungens*), and slimstem reedgrass (*Calamagrostis stricta*). The site is managed using patch-burn-grazing to simulate natural disturbances (See Fuhlendorf et al. 2009) by the Crane Trust for the benefit of s~~S~~andhill c~~C~~ranes, w~~W~~hooping c~~C~~ranes, other migratory birds, and ~~broadly endemic~~overall biological diversity. The second site was a wetland slough, which is a linear swale supporting wetland habitat (~~See~~ Meyer and Whiles 2008), in this case predominantly deep marsh, at Audubon's Rowe Sanctuary in Gibbon, Nebraska (40.667, -98.892). The slough exists within a former channel of the Platte River in a complex of accumulated sediment and deposited organic material, resulting from reduced flows over the last century, and one bank includes an erosion control structure built before 1938 as indicated by aerial photographs. The site is dominated by cattails (*Typha* spp.) and is immediately surrounded by trees including green ash (*Fraxinus pennsylvanica*), plains cottonwood (*Populus deltoides*) and woody shrubs including false indigo bush (*Amorpha fruticosa*). This forested slough~~is~~ site sits 22 m south of the Platte River's main (south) channel and retains standing water nearly year-round, while the wet meadow site rarely contains standing water beyond May (Brinley Buckley et al. *In Review*).

The two ~~acoustic sensors~~ autonomous acoustic recorders (Song Meter SM2+; Wildlife Acoustics) recorded for one minute out of every twenty minutes, starting on the hour, for a total

of three recordings per hour. The sampling rate was 44.1 kHz, the gain was 36 dB, and the bit depth was 16 bits. Each recorder had two omnidirectional microphones with a flat frequency response from 20 Hz –20 kHz. All incomplete recording days, defined as a day with less than the expected 72 one-minute recordings per 24-hour period, ~~(<72 recordings)~~ were removed prior to analysis. Recordings were then down-sampled to 8 kHz to accelerate computation time, as *P. Maculata* vocalizations average between 2.7 and 4.2 kHz throughout their range (Bee et al. 2010). Next, stereo tracks were converted to mono. In some cases, the sensitivity of one of the two microphones degraded over the course of deployment, possibly due to rain or other environmental factors. In these cases, we selected the channel of the intact microphone. If there were no microphone performance issues, the channel was randomly selected. ~~by selecting the track with greater high frequency sensitivity, as rain and other environmental factors can affect microphone sensitivity across mid- to high-frequencies (Brinley Buckley, 2018).~~ We quantified the acoustic activity of chorus frogs by detecting their calls with spectral cross-correlation. This method measures the pixel-by-pixel similarity between the spectrogram of a template call and the spectrogram of windowed selections of a recording (Hafner and Katz, 2018). This method is particularly effective for species with stereotypical calls, such as the characteristic call of *P. maculata*, a rising trill that sounds like fingers running across a comb. However, its call frequency, structure, and rate vary depending on environmental factors like temperature as well as physical characteristics (Bee et al. 2010). *P. maculata* also form choruses, where the calls of different individuals overlap. To accommodate this variation, we created 12 templates for the analysis consisting of a range of observed vocalizations (e.g. different fundamental frequency, call lengths, chorus densities; Table 1). To help quantify the diversity of call templates, we measured their center frequencies and inter-pulse distances in Raven 1.6.1 using spectrograms

that had a window length of 256 and a Hann window (Raven Pro: Interactive Sound Analysis Software, 2019). Center frequency refers to the frequency that divides a template recording into two frequency intervals containing equal energy. Inter-pulse distance refers to the interval between consecutive pulses within a call, from the offset of one pulse to the onset of the next; it was manually measured only for non-chorus templates by taking the mean of three inter-pulse intervals per clip. We used the R package “monitoR” (Hafner and Katz, 2018) to perform the spectral cross-correlation, utilizing a window length of 256 and a temporal overlap of 75%. Correlation peaks above a cutoff threshold of 0.4 were counted as detections. As we utilized 12 call templates, there was potential that different templates would detect the same chorus frog call, inflating the total count. To avoid duplicating detections, we divided each recording into 240, 0.25-second windows and counted a detection if the 0.25-second window contained one or more detections. To assess the accuracy of detection algorithms, we assessed their true positive rates by generating 1000 random files from February-May and manually identifying if *P. maculata* was detected via listening and visual inspection of spectrograms.

Template	Type	Low frequency (kHz)	High Frequency (kHz)	Center Frequency (kHz)	Duration (s)	Inter-Pulse Distance (s)
**Frog 1**	Chorus	2.5	4.0	3.2	0.30	NA
**Frog 2**	Chorus	2.7	3.6	3.1	0.30	NA
**Frog 3**	Chorus	2.6	4.0	3.3	0.31	NA
**Frog 4**	Chorus	2.6	4.2	3.3	0.30	NA
**Frog 5**	Chorus	2.6	4.0	3.2	0.30	NA
**Frog 6**	Individual	2.8	4.5	3.6	0.30	0.05
**Frog 7**	Individual	3.0	3.9	3.4	0.30	0.06
**Frog 5**	Individual	2.7	3.6	3.1	0.25	0.04
**Frog 9**	Individual	2.8	4.0	3.2	0.30	0.06
**Frog 10**	Individual	2.6	3.6	3.1	0.15	0.01
**Frog 11**	Individual	2.7	3.6	3.1	0.15	0.02
**Frog 12**	Individual	2.9	3.6	3.2	0.16	0.03

Table 1: Bioacoustic information for the 12 *Pseudacris maculata* call templates used in “monitoR”, including call type, lower and upper frequency bounds (of the template, not the call itself), center frequency, duration (of the template, not the call itself), and inter-pulse distance. Center frequency and inter-pulse distance were calculated using Raven 1.6.

As detections did not indicate or attempt to approximate a count or true abundance of *P. maculata*, vocalization detections were scaled to create a calling activity index from 0 to 1, where 0 indicates no vocalizations and 1 indicates the highest calling activity of *P. maculata* chorusing during the study. This represents a logical rescaling of count data to a ratio scale, which may help improve interpretation of our abundance index, which is both a reflection of local density as well as activity level (Schneider 2009, Pijanowski et al. 2011).

2.4. Environmental Variables

Weather data was acquired from the National Oceanic and Atmospheric Administration's National Centers for Environmental Information Global Historical Climatology Network Daily (Menne et al. 2012) obtaining data from Kearney, NE (station USC00254335) for the forested slough and Grand Island, NE (station USW00014935) for the wet meadow. Daily variables included average wind speed, total precipitation, mean, maximum, and minimum air temperature, snow accumulation and snow depth. Average wind speed was only available at the Grand Island weather station and was therefore used for both study sites. Hydropattern for the forested slough was obtained from a USGS stream gage for the Platte River at Kearney, Nebraska (USGS 06770200).

We derived vegetation phenology and water inundation data from analysis of time-lapse images from Mormon Island and Rowe Sanctuary Tower cameras of the Platte Basin Timelapse project. The time-lapse camera systems, consisting of a Nikon D300 DSLR with a 12.3 megapixel crop sensor and Nikkor 18-70mm 1:3.5-4.5 lens set at F/8 and powered by a solar panel, took one image every hour of sunlight. Images were processed by selecting daily images

~~taken between 10:00 and 14:00 to standardize for sun exposure and shadows. We then visually inspected the imagery collections, and images with obstructed views or inconsistencies, such as fog or spider webs, were removed from the analysis. Based on the digital number (DN) within the red-green-blue (RGB) colorspace of a pixel, we calculated green chromatic coordinates (GCC), a greenness index of vegetation phenology (Sonnentag 2012). At each site, we selected a region of interest (ROI) that encompassed vegetation and excluded sky and other features within the frame of view. GCC was calculated for each site's ROI using the equation GCC = G/[R + G + B] (Richardson 2018).~~

~~For the wet meadow at Mormon Island, we assessed water inundation through image-analysis using a macro script adapted from microscopy in the Java-based open-access program FIJI (Schindelin et al. 2012, Brinley Buckley et al. 2017). A ROI, separate from the ROI used to calculate GCC, was selected that excluded sky, horizon, and additional grassland landscape to reduce variability. The RGB colorspace was transformed to hue-saturation-value (HSV) to overcome potential limitations of the RGB color space for classifying water (Pekel et al. 2014). The ROI was converted to binary (0,1) by automated thresholding (threshold = (average background + average objects)/2). The binary masked results were visually inspected and manually compared to the original image for accuracy, and if a mask was visibly inaccurate, the original image was classified using heads up digitizing by manually tracing water inundation. A proxy for ponding surface water, the resulting water inundation (WI) values were the percent area classified as water within the region of interest. The final dataset was averaged into daily values and rescaled to a 0 to 1 value.~~

Formatted: Indent: First line: 1.27 cm

2.5. Statistical Analysis

All analyses characterizing the hourly and daily call activity of *P. maculata* were conducted within R (R Development Core Team, 2020). To assess diel activity we calculated how many recordings had at least one *P. maculata* vocalization identified out of 72 recording intervals per day, or the calling activity frequency. We set a threshold of 0.8 to represent a high frequency of calling, indicative of near 24-hour continuous call activity, and calculated monthly averages. We calculated summary statistics for all variables, in addition to calculating Spearman's Rank Correlation Coefficients to assess daily calling activity in association with environmental variables.

We used random forest regression to determine factors of importance for *P. maculata* calling activity using the "randomForest" package (Liaw and Wiener 2002). Random forest (RF) is a machine learning technique based on averaging a large collection of decision trees. Each tree is trained on a random bootstrap sample, while each split within a tree is sampled from a subset of candidate variables, making RF less susceptible to overfitting, more robust to correlations, and reducing variance compared to a single decision tree (Breiman 2001). RF models are non-parametric, non-linear, and can handle numerous continuous and categorical predictor variables. In addition, they can handle complex data with relatively limited observations (Cutler et al. 2012), a common issue with ecological data. RF provides a permutation-based measure of variable importance (VIMP), using mean squared error (MSE) for regression, which denotes the decrease in model accuracy given a variable is removed from the model. We built site-specific RF models with all candidate predictor variables including wind (WND; average wind speed; mph), precipitation (PRCP; daily accumulation; cm), 7-day precipitation (PRCP7; weekly accumulation; cm), 1-day lag precipitation (PRCP1; day prior daily accumulation; cm), daily mean, maximum, and minimum air temperature (TAVG, TMAX, TMIN; °C), day of year

(DOY), green chromatic coordinates (GCC), snow (SNW; daily accumulation; cm), snow depth (SNWD; cm), and included year to account for annual variability. Hydropattern was also included in the models, with water inundation (WI) included for the wet meadow and Platte River streamflow (CMS; m^3/s) at the forested wetland. The number of variables selected as candidates at each split was set to three, and the model was tested and pruned to 500 trees to reduce overfitting. Variables were ranked by variable importance score, and variables with negative MSE scores, indicating they negatively influenced prediction accuracy, were removed from the model. Relationships between environmental variables and *P. maculata* activity were examined using partial dependence plots, which predicts the response of *P. maculata* to a given variable holding all other variables in the model at their mean.

3. Results

From 2015 to 2017 we totaled 472 recording days (227 at wet meadow/Mormon and 245 at forested slough/Rowe) during the spring breeding season (March – June). During the three years of our study, data were missing for 49 days at the wet meadow (18%) and 31 days at the forested slough (11%) due to equipment malfunction or removal of days with incomplete recording intervals. Assessment of detection algorithms resulted in an overall true positive (TP) rate of 97.8% (n=1000). At Mormon Island, the TP rate was 98.4% (n=853), and at the forested slough, the TP rate was 94.6% (n=147). For the sampling duration, mean daily average temperature was approximately 8 °C, ranging from approximately -15 to 24 °C (Table 2). Mean daily precipitation accumulation was approximately 0.2 cm and ranged from 0 to 5.23 cm at Mormon and 0 to 10.16 cm at Rowe, with highest mean and median precipitation in 2016 (Figure 1). Mean streamflow for the Platte River was 85 m^3/s, with the highest mean and median streamflow occurring in 2016 (107 m^3/s; Figure 1). Mean streamflow was highest in May

followed by March, with the highest variability in May 2015. Contrastingly, wet meadow inundation had the highest mean in March of 2016 and 2017 and was relatively similar across months in 2015, while inundation had the greatest range in April of all years (Figure 1). The wet meadow had the highest mean inundation in 2016 (0.51), in addition to the highest peak extent of inundation (Table 2).

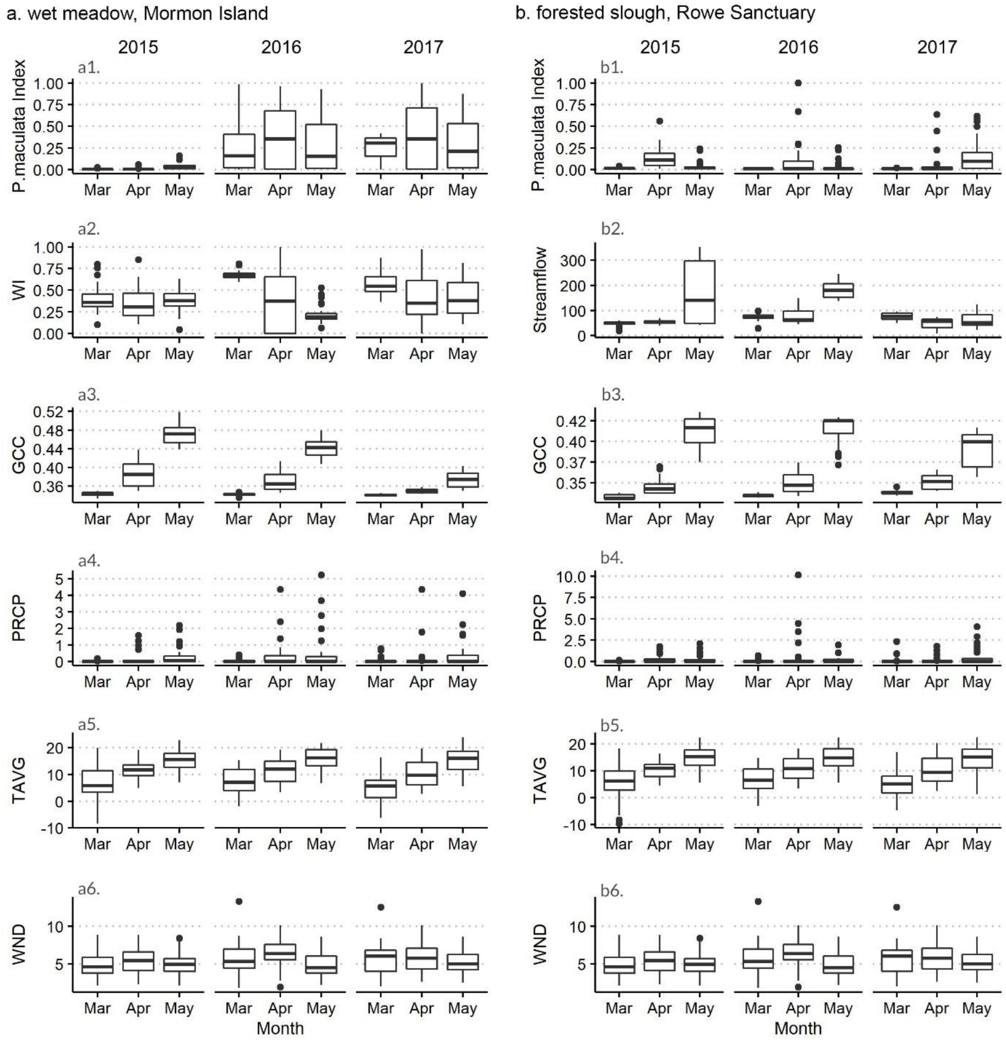

Figure 1. Monthly boxplots at wet meadow habitat (a.) and forested slough (b.) illustrating the distribution, median, and outliers for *Pseudacris maculata* calling activity index [a1, b1], hydropattern (WI; water inundation [a2], CMS; streamflow CMS[b2]), vegetation (GCC; green chromatic coordinates [a3,b3]), precipitation (PRCP [a4,b4]), temperature (TAVG [a5,b5]), and wind speed (WND [a6,b6]).

Wet meadow

		Count	WND	PRCP	TAVG	GCC	WI
2015	min	0	2.1	0	-15.3	0.33	0.04
	max	0.16	11.2	2.18	22.9	0.52	0.85
	mean	0.01	5.16	0.14	7.3	0.38	0.45
	sd	0.03	1.71	0.38	8.98	0.06	0.2
2016	min	0	0	0	-8.7	0.33	0
	max	0.98	15.2	5.23	21.7	0.48	1
	mean	0.29	5.75	0.3	9.19	0.37	0.51
	sd	0.32	2.12	0.89	6.98	0.04	0.29
2017	min	0	2	0	-8.2	0.33	0
	max	1	12.5	4.34	24	0.4	0.97
	mean	0.23	5.47	0.19	8.61	0.35	0.44
	sd	0.31	1.82	0.65	7.31	0.02	0.2
All years	min	0	1.8	0	-15.3	0.33	0
	max	1	15.2	5.23	24	0.52	1
	mean	0.19	5.46	0.21	8.37	0.37	0.47
	sd	0.29	1.9	0.68	7.82	0.04	0.23

Forested slough

		Count	WND	PRCP	TAVG	GCC	CMS
2015	min	0	2.1	0	-15.85	0.33	18.15
	max	0.56	11.2	2.08	22.25	0.44	353.96
	mean	0.08	5.16	0.14	6.87	0.36	85.52
	sd	0.11	1.71	0.37	8.82	0.04	78.85
2016	min	0	1.8	0	-7.75	0.33	28.6
	max	1	15.2	10.16	22.2	0.43	246.64
	mean	0.05	5.75	0.24	8.4	0.36	107.26
	sd	0.14	2.12	1.08	6.68	0.04	50.13
2017	min	0	2	0	-8.85	0.33	8.72
	max	0.63	12.5	4.06	22.5	0.42	124.03
	mean	0.06	5.47	0.21	8.38	0.35	62.67
	sd	0.14	1.82	0.62	7.19	0.02	21.92
All years	min	0	1.8	0	-15.85	0.33	8.72
	max	1	15.2	10.16	22.5	0.44	353.96
	mean	0.06	5.46	0.19	7.88	0.36	85.21
	sd	0.13	1.9	0.75	7.63	0.03	58.18

Table 2. Summary statistics (min = minimum, max = maximum, mean, and sd = standard deviation) for *Pseudacris maculata* calling activity (Count), average wind speed (WND), daily precipitation accumulation (PRCP), average air temperature (TAVG), and green chromatic coordinates (GCC). Hydropattern also was included as water inundation (WI) at the wet meadow (Mormon Island) and streamflow (CMS) at the forested slough (Rowe Sanctuary).

For the duration of the study, *P. maculata* vocalizations on average were highest between 20:00 and 01:00 (8:00pm to 1:00am) at both sites, and activity decreased between 01:00 and 02:00 markedly (Figure 2). Calling activity increased moderately between 08:00 and 15:00 in the wet meadow, however this trend was not observed at the forested wetland. Monthly averaged calling frequency, with a threshold of 0.8, also differed between habitats. At the wet meadow, *P. maculata* called near continuously 18% of days in March, 30% of days in April, and 37% of days in May. Comparatively, at the forested slough, *P. maculata* called continuously 0% of days in March, 9% of days in April, and 6% of days in May (Figure 3). In addition, calling frequency differed by year, with nearly continuous calling occurring at the wet meadow more than 40% of the time in April and May of 2016 and 2017 (Figures 1 and 3).

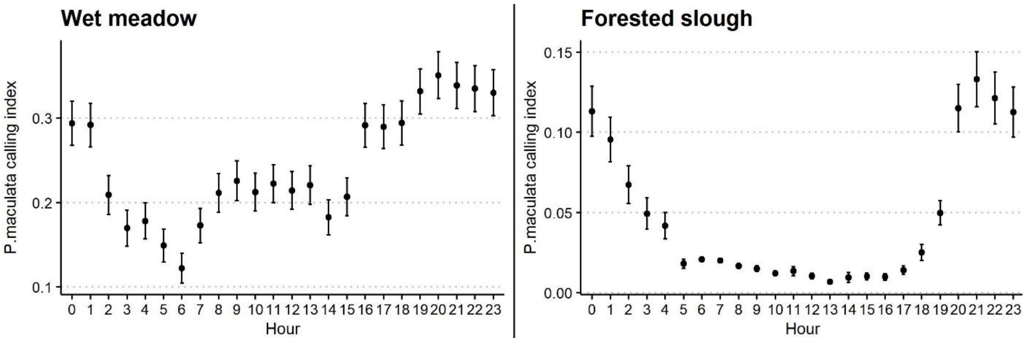

Figure 2. ~~Average~~ Mean h~~H~~ourly calling activity for Pseudacris maculata during spring (March – June) of 2015 – 2017 within wet meadow habitat on Mormon Island and forested slough habitat at Rowe Sanctuary. Of note, y-axis scale differs for wet meadow (0.1 – 0.3) and forested slough (0 – 0.15).

At both sites, *P. maculata* calling activity was highest during April of all years, with the exception of the forested slough in 2017, where vocalizations were greater in May (Figure 3). Generally, calling activity was sustained from March to May, with vocalizations beginning on average the first week of March (mean DOY 60), peaking from early to mid-April depending on site (mean DOY 92 at Mormon, mean DOY 111 at Rowe), with a second peak occurring the first week of May (mean DOY 124).

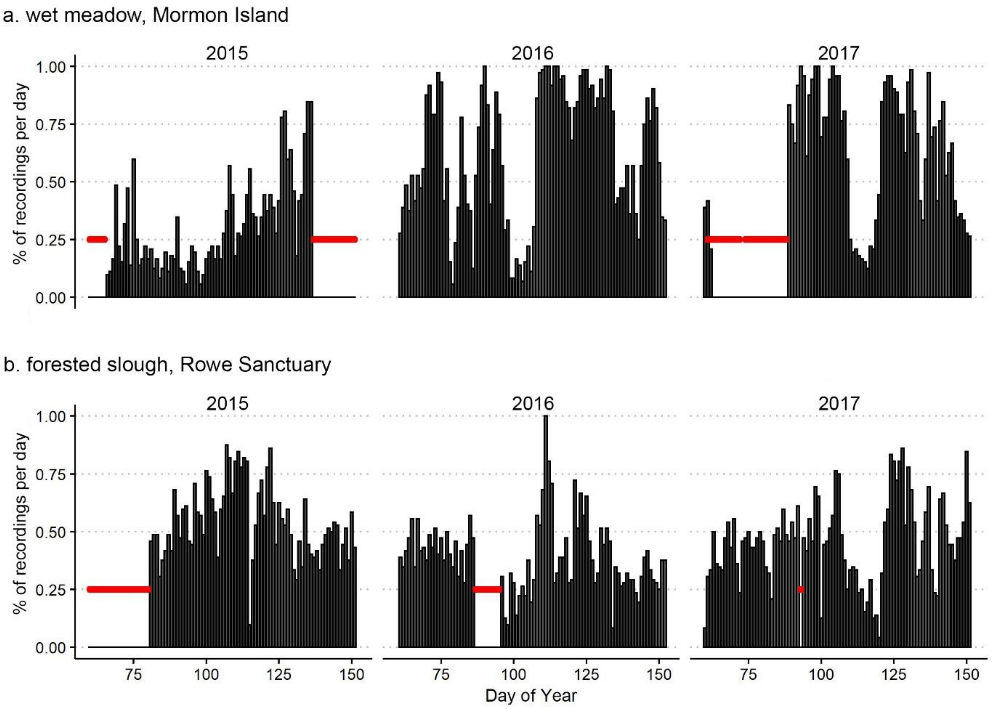

Figure 3. Percent of recordings with *Pseudacris maculata* detections present per day at a wet meadow (a.) and forested slough (b.) in the Central Platte River Valley of Nebraska. Red lines indicate missing data. ~~were missing.~~

Snow and snow depth negatively contributed to the predictive accuracy of the random forest models at both sites and therefore were removed from the final models. Once the models were adjusted, the RF model explained 73% of the variance in *P. maculata* calling activity at the wet meadow (MSE = 0.02), while for the forested slough, the RF model explained 45.3% (MSE

= 0.01) (Table 3). Year, day of year, hydropattern, green chromatic coordinates, average temperature, and 7-day total precipitation were the highest ranked variables of importance at both sites, indicating removal of these variables from the models would decrease predictive accuracy substantially. *P. maculata* calling activity was predicted to begin increasing at DOY 60 and then substantially at DOY 70 at the wet meadow and to increase more gradually at DOY 60 followed by a substantial increase at DOY 90 at the forested slough (Figure 4). Similarly, at both sites activity was predicted to begin increasing at temperatures above 5°C, and in response to GCC, peak at 0.35. Activity increased at seven-day precipitation accumulation levels of approximately 0.25 cm at the wet meadow and at approximately 3 cm at the forested slough. Hydropattern was an important variable at both sites, with *P. maculata* activity predicted to increase with inundation levels above 0.2 at the wet meadow and predicted activity peaking at streamflow of approximately 50 m^3/s, declining with increases thereafter. There was also an observed difference in activity based on year, with activity highest in 2016 and 2017 at the wet meadow, and 2015 and 2017 at the forested slough.

a. Wet meadow
Mean of squared residuals: 0.02
Variance explained: 72.7%

Variable	% Inc. MSE
Year	36.5
DOY	31.9
WI	28.5
GCC	22.0
PRCP7	18.9
TAVG	17.9
TMIN	15.8
TMAX	13.6
PRCP1	5.2
PRCP	4.0
WND	3.1

b. Forested wetland
Mean of squared residual: 0.01
Variance explained: 45.3%

Variable	% Inc. MSE
PRCP7	20.13
GCC	13.42
DOY	12.85
CMS	10.56
Year	8.08
TAVG	7.4
PRCP1	5.08
TMIN	4.46
TMAX	4.42
WND	4.07
PRCP	2.64

Table 3. Results of random forest models predicting *Pseudacris maculata* calling activity at a wet meadow and a forested slough. Variable importance scores ranked from most important (greatest percent increase in mean squared error) to least for all eleven predictor variables. Predictor variables including wind (WND; average wind speed;

mph), precipitation (PRCP; daily accumulation; cm), 7-day precipitation (PRCP7; weekly accumulation; cm), 1-day lag precipitation (PRCP1; day prior daily accumulation; cm), daily mean, maximum, and minimum air temperature (TAVG, TMAX, TMIN; °C), day of year (DOY), green chromatic coordinates (GCC), snow (SNW; daily accumulation; cm), snow depth (SNWD; cm), and year (Year).

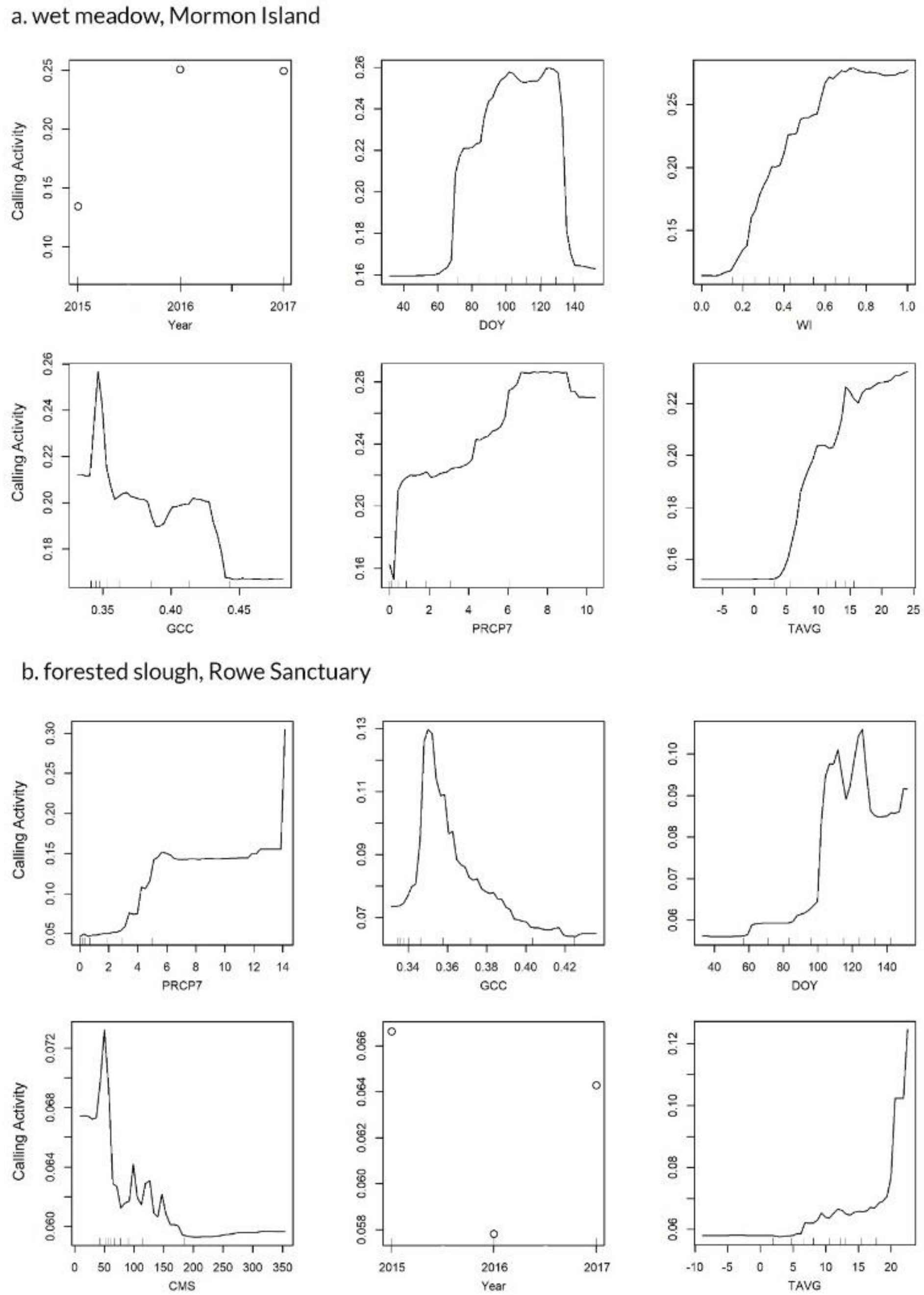

Figure 4. Partial dependence plots showing predicted response of *Pseudacris maculata* to the six variables with the highest variable importance scores from random forest models at a wet meadow (Mormon) and forested slough (Rowe) in central Nebraska.

4. Discussion

~~The~~ In the Great Plains, the life cycle and behavioral activity of *Pseudacris maculata* is closely tied to the grasslands ~~and~~ and interspersed wetlands~~ of the Great Plains~~. However, these habitats ~~are~~ face~~d with~~ increasing pressure from human development, agriculture, water-use changes, and a globally changing climate (Sidle 1989, LaGrange 2005, Matthews et al. 2013). Multimodal monitoring—the ~~,~~pairing of autonomous sound recorders, time-lapse cameras, and additional sensors—~~,~~ collects ~~a fusion of~~ data streams capable of reflecting changes in environmental and habitat conditions, as well as vocal-taxa communities. ~~Through~~Using multimodal monitoring, ~~-Amphibian breeding is often driven by environmental conditions, but little is known about the breeding phenology of *P. maculata* within prairie-wet-meadow ecosystems. Our study~~w~~W~~e ~~assessed~~ identified the spring calling phenology of *P. maculata* in central Nebraska ~~calling~~ and found that vocalization activity ~~in relation to environmental conditions and demonstrated the application of multimodal passive monitoring technologies. Our results indicated calling activity~~ was highly influenced by weather and habitat conditions.

~~*P. maculata* vocalization activity occurs predominately in the spring across their wide geographic extent, with precise timing of breeding activity dependent on latitude and elevation in addition to environmental conditions (Dodd 2013). In Quebec, Canada, near the northern extent of their range, Ouellet et al. (2009) found *P. maculata* initiated calling in late May. In higher elevations, such as the Rocky Mountains, *P. maculata* calling activity begins in May and is associated with snowpack melt (Corn et al. 2000, Muths et al. 2016). In New Mexico, breeding occurs from February to August (Degenhardt et al. 2005), and similar *Pseudacris* spp. have been reported to call as early as January and into March in Texas (Saenz et al. 2006).~~ *P. maculata*

vocalization activity occurs predominately in the spring across the species'~~ir~~ wide geographic extent, with precise timing of breeding activity dependent on latitude and elevation in addition to environmental conditions (Dodd 2013). Near the northern extent of *P.maculata*'s range in Quebec, Canada, and at higher elevations such as the Rocky Mountains, calling often begins in May and is associated with snowpack melt (Ouellet et al. 2009, Corn et al. 2000, Muths et al. 2016). In the southern extent of their range in New Mexico, breeding occurs from February to August (Degenhardt et al. 2005), and similar *Pseudacris* spp. have been reported to call as early as January and into March in Texas (Saenz et al. 2006). Prior studies utilizing trapping techniques in the Central Platte River Valley of Nebraska ~~have utilized~~~~utilizing~~~~and~~ found *P. maculata* ~~calling~~ activity was highest in mid-April (Jones et al. 1981, Goldowitz and Whiles 1999), with calling observed as early as 18 March (Geluso and Harner 2013a). Although beneficial for specific questions, manual survey methods can have limitation for understanding phenological timing and associated drivers, and many surveys often did not begin until April (Jones et al. 1981, Goldowitz and Whiles 1999, Geluso and Harner 2013a). Furthermore, trapping surveys are likely more reflective of movement than calling though both behaviors may be associated with reproduction (Spencer 1964, Kramer 1973). We observed *P. maculata* vocalization beginning in early March, often continuing into late May, and peaking in April. D~~There was d~~ifferential activity patterns were observed between the wet meadow and forested slough, with *P. maculata* chorusing increasing at approximately day of year 70 (10 March) at the wet meadow and at day of year 100 (9 April) at the forested slough. This spring phenological pattern of *P. maculata* overlaps with the migratory timing of endangered whooping cranes in the CPRV, which on average occurs from late March through mid-April, with a mean initial Julian day of 92 (+/- 1; 2 April; Silcock and Jorgenson 2020). As whooping cranes stop to refuel and

Formatted: Font: Italic

forage in sloughs and wet meadows along the Platte River, the overlapping phenology in conjunction with prior literature (Archibald and Meine, 1996, Bergeson et al. 2001, Geluso and Harner 2013b, Divets 2016) suggests that *P. maculata* and similar anuran species may be an available food resource for whooping cranes during their migration (Jones et al. 1981, Bergeson et al. 2001, Johnsgard 1983, Geluso et al. 2013, Caven et al. 2019b). However, more research is needed, as little is known -regarding whooping crane diet -this phenological potential interactions between cranes and prey throughout most of the Great Plains (Caven et al. 2019b)., needed, including an assessment of phenological shifts in prey availability in relation to migration timing. , especially related to changing phenology of these interactions.

We observed vocalization activity beginning in early March and often continuing into late May. *P. maculata* calling activity was predicted to begin around day of year 60 (1 March) in the wet meadow, and to increase later, approximately day of year 90, in the forested slough (10 April; Figure 4). In addition, activity generally peaked in mid-April and again in mid-May at the forested slough, and peak activity was broadly more stochastic and frequent at the wet meadow, often with near-consistent vocalizations from mid-March declining into April followed by another peak into May. Differing hydrological conditions at the two assessment wetlands may explain the temporal differences in peak calling activity between sites. Groundwater levela major driver of wetland inundation (Wesche et al. 1994, Davis et al. 2006) is generally highest within the CPRV in March and is a major driver of wetland inundation (Wesche et al. 1994, Davis et al. 2006). High groundwater levels may have provided an ideal level of inundation for breeding at the wet meadow site, but generally too much water for breeding at the forest slough wetland, which consisted of topographically deeper marsh habitat (Kantrud et al. 1989, Whiles and Goldowitz 1998, Goldowitz and Whiles 1999, Dodd et al. 2013). It is also of note that calling

activity increased following much higher weekly rain totals at the forested slough (3.0-6.0 cm; Figure 4) than at the wet meadow (0.2-0.5 cm; Figure 4), and that these large rain events most commonly occurred at the site in April when calling activity peaked (Figures 2, 3). It is possible that breeding activity also occurred in temporary pools outside of the forested slough following significant precipitation accumulation. This would explain why the most important variable at the forested slough was 7-day precipitation, while it was the 5th most important variable at the wet meadow. Furthermore, if breeding occurs in the forested slough within a narrow band of hydrological conditions as indicated by the quadratic relationship of *P. maculata* calling activity to streamflow, and also outside of this wetland in temporary pools following high rain events, it could explain the poorer performance of the Random Forest regression model. *P. maculata* only intermittently use the deeper wetland habitats associated with the forested slough as well as irregularly and ephemerally inundated depressions adjacent to the site as conditions are appropriate (Goldowitz and Whiles 1999, Dodd 2013).

In the Central Platte River Valley of Nebraska, prior studies have broadly documented similar phenological timing of spring *P. maculata* observations, however most studies did not begin until April (Jones et al. 1981, Goldowitz and Whiles 1999). Jones et al. (1981) found calling was highest in mid-April and chorusing declined by May 10th. Similarly, Goldowitz and Whiles (1999) found calling activity was highest in April. Geluso and Harner (2013a) observed *P. maculata* calling as early as 18 March (Geluso and Harner 2013a). The spring call phenology of *P. maculata* overlaps with the timing of Whooping Crane stopovers during spring migration in the CPRV. On average in the CPRV, Whooping Crane migration occurs from late March through mid-April, with most stopovers between 30 March and 21 April with a mean initial Julian day of 92 (+/- 1; 2 April; Silcock and Jorgenson 2020). As Whooping Cranes stop to refuel and forage

~~in sloughs and wet meadows along the Platte River, the overlapping phenology in conjunction with prior literature suggests that *P. maculata* and similar anuran species may be an available food resource for Whooping Cranes during their migration (Jones et al. 1981, Bergeson et al. 2001, Johnsgard 1983, Geluso et al. 2013). There are anecdotal and observational reports of Whooping Cranes eating reptiles and amphibians (Archibald and Meine, 1996, Geluso and Harner 2013b), however the limited numbers of Whooping Crane individuals, endangered species status, and extent of their geographic movement during migration make rigorous observations of predation challenging. Geluso et al. (2013) observed a Whooping Crane in the CPRV consuming a Plains Leopard Frog. Johnsgard (1983) suggests frog egg-masses may be prey for WHCR during migration, while Divets (2016) reports Whooping Crane in Louisiana eating anurans, including *Pseudacris* spp. tadpoles and froglets. Bergeson et al. (2001) documented adult Whooping Crane predating on *P. maculata* to feed to a colt on their breeding grounds in Wood Buffalo National Park, Canada. Published observations of Whooping Crane predation are limited during their migration, especially through Nebraska (Caven et al. 2019b), and more studies are needed to determine if *P. maculata* may be an important food resource there.~~

~~During our study,~~ Acoustic metrics indicated *P. maculata* vocalizations at the wet meadow ~~in 2015~~ were much lower in 2015 than in the following two years. ~~We~~ Upon examin~~ed~~ing spectrograms and listen~~ed~~ing to ~~the~~ audio recordings, ~~which revealed~~the chorusing of *P. maculata* was faint and sounded distant during spring of 2015, especially compared to 2016 and 2017, while other ~~. Other~~ sounds ~~during this time~~, such as r~~R~~ed-winged blackbirds (*Agelaius phoeniceus;* call frequencies of 1 – 5 kHz) and s~~S~~andhill~~s~~ c~~C~~ranes (*Antigone canadensis;* call frequencies of 0.5 – 8 kHz), ~~exhibit similar~~had similar sounding proximity and amplitude

between years. ~~,~~ T~~and t~~herefore, the observed decline seems explicit to *P. maculata*. S~~Although s~~pecific drivers of this decline are uncertain, albeit, we ~~surmise~~ suggest potential influences were related to events that occurred prior to spring 2015. In spring of 2013 and 2014, controlled burns were set to over 375 and 360 acres on Mormon Island, respectively, which contained much of the locally available wet meadow habitat. Although literature is sparse ~~with~~ regarding ~~various and species-specific~~ responses of anurans to prescribed fire (Wilgers 2006, Harner and Geluso 2011), the burns may have had ~~indirect and~~ direct impacts on *P. maculata* ~~activity~~, including mortality of adults, larva, or egg masses (Pilliod et al. 2003, Bowles and Jones 2013), or indirect impacts through alteration of habitat components, such as a rapid increase in water temperature, altered hydroperiod, changes in water chemistry, interstitial-filling of ash and sediments, or changes in vegetation density (Brown et al. 1990, Pilliod et al. 2003, Caldwell et al. 2013). ~~(Pilliod et al. 2003). The~~ As the area of wet meadow burned contains a high density of ephemeral and intermittent ~~shallow pools and sloughs that are inundated in the spring~~wetlands, and given that dispersal distances of *P. maculata* are limited and usually under 250 m (Spencer 1964, Werner et al. 2009, Scherer et al. 2012), ~~, which *P. maculata* generally prefers for breeding (Whiles and Goldowitz 1998, Goldowitz and Whiles 1999, Ballinger et al. 2010, Dodd et al. 2013). Therefore,~~ it is likely that a high density of *P. maculata* were present in this area at the times of the fires. ~~. Furthermore, with limited information on *P. maculata* responses to fire regimes, there is potential the species is immediately sensitive to fire disturbance, however, there could be long-term habitat-related advantages (Pilliod et al. 2003). After breeding or metamorphosis, *P. maculata* and similar *Pseudacris* species migrate into grasslands or other terrestrial habitats, however dispersal distances are limited and usually under 250 m and rarely exceed 600 m (Spencer 1964, Werner et al. 2009, Scherer et al. 2012). As dispersal patterns are linked across~~

Formatted: Font: Italic

the aquatic and terrestrial landscape, fire may alter habitat through modification of vegetation density or interstitial filling of ash and sediments (Pilliod et al. 2003). For anurans in general, habitat management through prescribed fire may have indirect medium- or long-term benefits, as it increases habitat heterogeneity, nutrient availability, reduces woody encroachment, and maintains vegetation species richness over time, while having direct and negative short-term impacts such as mortality of adults, larva, or egg masses (Pilliod et al. 2003, Bowles and Jones 2013). Prior studies report ambiguous findings, including declines in populations following fire (Driscoll and Roberts 1997), observations of anurans seeking refuge and fleeing from the sound of fire (Grafe et al. 2002), and species-specific responses to various burn regimes (Wilgers 2006). However, the response of anurans to prescribed burns is likely mediated by compounding influences, such as life stage, environmental conditions, topography, region, fire severity, vegetation, and time since burn (Pilliod et al. 2003, Westgate et al. 2017). Depending on conditions, fire may have consequential impacts including a rapid increase in water temperature, altered hydroperiod, or changes in water chemistry (Brown et al. 1990, Caldwell et al. 2013). Following a period of drought in 2012 and 2013, wet meadow habitat was drier than average during the spring of 2013 and 2014, reducing the availability of ponded water, and thus aquatic refugia from the fire, which could have increased both direct and indirect impacts on *P. maculata* (Wesche et al. 1994, Pilliod et al. 2003, Dave et al. 2006). It is possible the area was not fully recolonized until sometime before the spring 2016 breeding season. Relatedly, Browne et al. (2009) found that *P. maculata* abundance was associated with habitat features on a larger scale than other anurans in the Boreal Plains of Canada, and suggests that this reflects the comparatively large amount of terrestrial habitat adjacent to breeding ponds utilized throughout their lifecycle. For anurans in general, habitat management through prescribed fire may have

indirect medium However, prescribed fire may have or long-term benefits, as it increases habitat heterogeneity, nutrient availability, reduces woody encroachment, and maintains vegetation species richness over time , while having direct and negative short-term impacts such as mortality of adults, larva, or egg masses (Pilliod et al. 2003, Bowles and Jones 2013).

Another possible explanation for the decline in activity was intensive grazing (> 300 cow-calf pairs) from mid-April 2014 to early October of the wet meadowthatwhichintensively (> 300 cow-calf pairs) , potentially influencing *P. maculata* activity indirectly through defoliation as *Pseudacris* species generally prefer habitats with significant vegetative cover (>75%) of at least moderate height (> 30 cm) (Anderson et al. 1999, Mannan 2008). Alternatively, *P. maculata* activity could have been reduced in 2015 as a result of cChytrid fFungus (*Batrachochytrium dendrobatidis*). Harner et al. (2011, 2013) first documented the fungal disease in the CPRV at our study site in 2010 in American bullfrogs, then later in Woodhouse's tToads and pPlains lLeopard fFrogs. Although cChytrid fFungus was not documented in boreal chorus frogs in the CPRV at the time, high rates (> 80%) of *P. maculata* mortality resulting from Chytridiomycosis have been documented elsewhere throughout its range (Hyman 2012). . As *P. maculata* takes 1-2 years to reach sexual maturityMortality generally occurs 20 to 30 days after *B. dendrobatidis* exposure ,and higher water temperatures decrease infection rates and morbidity/mortality rates (Hyman 2012, Amburgey 2014). As *P. maculata* reproduction occurs soon after spring emergence, many individuals likely may have had time to lay eggs ahead of potential adult mortality from Chytridiomycosis, whichand as a result of warming waters likelycould have had minimalminimized potential impacts on larvae development (Hyman 2012). Therefore, as *P. maculata* takes 1-2 years to reach sexual maturity, high localized mortality in 2014 could have resulted in low localized calling during the following spring in

Formatted: Font: Italic

Formatted: Font: Italic

Formatted: Font: Italic

Formatted: Font: Italic

Formatted: Font: Italic

2015 -(Hyman 2012, Amburgey 2014). Our data provides some support for this theory as there was a slight uptick in calling during May compared to March and April of 2015, aligned with the time the first tadpoles hatched in 2014 would reach sexual maturity and potentially become reproductively active (Amburgey 2014). Given the lack of information on fire regime impacts to anurans, especially in grassland systems, further studies are necessary to understand species-specific responses and to guide sound conservation practices. While the cause is uncertain, the observed decrease in *P. maculata* vocalization emphasizes the importance of monitoring and the use of quantifiable measures, such as acoustic metrics, to identify disturbances which may be indicative of longer-term changes, management actions, or an abrupt shift in ecological systems.

In addition to fire, other management activities or a disease outbreak are possible explanations for the decreased *P. maculata* activity in 2015. Of note, the pasture that contained the wet meadow was grazed from mid-April 2014, following the controlled burn, to early October with 300 cow-calf pairs. Mannan (2008) found that defoliation resulting from mid-continent Light Geese (*Chen* spp.) grazing had a negative impact on *P. maculata* presence. Similarly, Anderson et al. (1999) found that anuran communities, including the spotted chorus frog (*Pseudacris clarkii*), preferred areas with more vegetative cover (> 75%) of at least moderate height (> 30 cm). An alternative hypothesis, *P. maculata* activity could have been reduced in 2015 as a result of a disease outbreak. Harner et al. (2011, 2013) first documented Chytrid Fungus (*Batrachochytrium dendrobatidis*) in the CPRV at our study site in 2010, first in American Bullfrogs, then later in Woodhouse's Toads and Plains Leopard Frogs. Harner et al. (2011, 2013) did not document Chytrid Fungus in Boreal Chorus Frogs. However, Hyman (2012) had documented high rates (> 80%) of *P. maculata* mortality resulting from Chytridiomycosis in Arizona. As *P. maculata* takes 1-2 years to reach sexual maturity, high

Formatted: Font: Italic

Formatted: Font: Italic

localized mortality in 2014 could have potentially resulted in low localized calling during the following spring in 2015 (Amburgey 2014). Our data provides some support for this theory as there was a slight uptick in calling during May compared to March and April of 2015, aligned with the time the first tadpoles hatched in 2014 would reach sexual maturity and potentially become reproductively active (Amburgey 2014).

Although we are unsure of the specific decrease in *P. maculata* calling observed in 2015, change in vegetation structure from fire or grazing may have influenced the species behavior. *P. maculata* use vegetation for various reasons; they will deposit egg masses beneath partially submerged or emergent vegetation, utilize branching vegetation for breeding displays, and vegetation may provide structural cover and protection from predators (Anderson et al. 1999, Mannan 2008, Ballinger et al. 2010). Anderson et al. (1999) found *P. clarkia* was found more often in playa wetlands with taller and denser vegetation that offered greater cover. *P. maculata* response to green chromatic coordinates, a vegetation index indicative of phenology, was predicted to peak at 0.35, which generally aligns with when vegetation begins to green-up, before declining steadily to minimal calling activity around 0.41, at which time vegetation would likely be dense and in full bloom, offering cover and protection for *P. maculata* post-breeding and natal dispersal into terrestrial environments (Wells 2010, Malzahn et al. *In Review*). Although random forest modeling utilizes bagging and thus is known to be more robust in handling complex and interacting data (Breiman 2001), some studies suggest that there is potential for bias in the measures of importance for highly correlated variables (Strobl et al. 2008). Given the high correlation of vegetation and Julian day (r = 0.8), we recognize the limitations of this approach and acknowledge the possibility for inflating the importance of these variables. However, considering the iterations of randomness introduced within random forest

Comment [EBB1]: Maybe take out most of this paragraph if necessary to cut more?

models, examination of the partial dependence plots, and the *a priori* importance of both variables, we chose to include both vegetation and Julian day. As a proxy for seasonality, both vegetation and Julian day may offer useful information into the phenological pattern of *P. maculata* activity. Individually, they may provide different insight into potential long-term decoupling from climatic shifts, such as earlier breeding activity (Julian day) or potential resource mismatches (vegetation) (Klaus and Lougheed 2013, Visser and Gienapp 2019).

~~Although~~ While there is a geographic difference in the timing of call activity based on latitude and elevation, *P. maculata* and similar *Pseudacris* species have been found to occupy a comparable temperature regime across their distribution. Our findings indicate *P. maculata* activity increased when average air temperature exceeded 5°C, an observation synonymous with prior studies across the geographic range of *P. maculata*. Oullete et al. (2009) found *P. maculata* vocalizations began when temperatures were greater than 5°C in the Rocky Mountains, while Saenz et al. (2006) found *Pseudacris* species in Texas called at temperatures ranging from 5° C to 22°C (41°F to 72°F). Clarke 1958 reported *P. maculata* vocalized between air temperature ranges of approximately 4.5°C to 32°C (40°F to 90°F). In addition, we found a decline in calling activity when temperatures dropped below 5°C, such as in late March of 2016 and mid-April of 2017. Relative to other anuran species, *P. maculata* has been shown to breed earlier and in colder temperatures ~~. For example, *Rana sylvatica* (Wood frogs) are known to chorus when air temperatures exceed 8-10°C~~ (Howard 1980). *Lithobates pipiens* (n~~N~~orthern l~~L~~eopard frogs) were observed initiating calling 8-11 days after ice melt on an ephemeral breeding pond (Sommers et al. 2018). From the time-lapse images, we approximated an ice-off date of the wet meadow during our study and found that *P. maculata* on average started calling approximately 5 days after ice-melt. Similar to the snow-melt driven ponds in higher elevations (Corn 2003), snow and

ice melt resulting from warming seasonal temperatures may act as an environmental cue (Corn and Muths 2002).

As *P. maculata* relies on ponded water for critical stages in their life cycle, including breeding, egg laying, and metamorphose, hydropattern is likely a key determinant of activity patterns (Saenz et al. 2006, Ballinger et al. 2010). Prior research shows *P. maculata* is adapted to local hydroperiod and lacks plasticity, suggesting their ability to respond to changes in hydropattern may be limited (Amburgey et al. 2012). Within the Central Platte River Valley, most wetlands are connected to the hydrology of the Platte River (Henszey and Wesche 1993), however, water and land use changes have greatly altered the remaining wetlands and their hydropattern (Sidle et al. 1989). Our results indicate hydropattern was an important variable at both the wet meadow and forested slough, with slight differences in the response of *P. maculata* calling likely a factor of site-specific hydrological conditions. In mid-April 2016 as well as the end of April and beginning of May 2017, *P. maculata* calling decreased precipitously at both sites, concurrent with a decline in wet meadow inundation and decrease in Platte River streamflow, suggesting the decrease in *P. maculata* vocalization was likely attributed to water availability. Calling activity increased following much higher weekly rain totals at the forested slough (3.0-6.0 cm; Figure 4) than at the wet meadow (0.2-0.5 cm; Figure 4), where these large rain events most commonly occurred at the site in April when calling activity peaked (Figures 2, 3). It is possible that breeding activity also occurred in temporary pools outside of the forested slough following significant precipitation accumulation, explained by the high importance rank of 7-day precipitation at the forested slough but less important at the wet meadow. However, seven-day rainfall accumulation was an important predictor of *P. maculata* calling activity at both sites, indicating precipitation regimes sufficient to inundate temporary and intermittent

Formatted: Font: Italic

wetlands likely act as an environmental cue for opportunistic *P. maculata* breeding activity (Saenz et al. 2006, Ballinger et al. 2010).

Hydropattern was an important variable at both sites, albeit, site specific differences in hydrology and resulting characteristics potentially influenced *P. maculata* calling at the wet meadow compared to the forested slough. Although calling activity generally peaked in April at both sites, peak activity was broadly more stochastic and frequent at the wet meadow, often with near-consistent vocalizations from mid-March declining into April followed by another peak into May. As groundwater, a major driver of wetland inundation (Wesche et al. 1994, Davis et al. 2006), is generally highest in March, high levels may have provided suitable inundation for breeding at the wet meadow site, but generally too much water for breeding at the forested slough, which consisted of topographically deeper marsh habitat (Kantrud et al. 1989, Whiles and Goldowitz 1998, Goldowitz and Whiles 1999, Dodd et al. 2013). Prior research suggests *P. maculata* prefers intermittent wetlands, likely as an avoidance of potential desiccation from quick-drying shallow wetlands and to avoid predation from fish and other frog species within more permanent bodies of water (Kolozsvary and Swihart 1999, Dodd 2013). In the CPRV, *P. maculata* was found to be most abundant in wetlands with water present 60-90% of the time (Goldowitz and Whiles 1999), while they were not observed in perennial waters (Geluso and Harner 2013a). -*P. maculata* calling activity was predicted to increase when water inundation was above 0.2 at the wet meadow, which remained inundated for most of the spring breeding season. At the forested slough, *P. maculata* activity was predicted to increase when streamflow reached 50 m^3/sec (~1766 ft^3/sec; Figure 4), and when streamflow exceeded this, *P. maculata* activity was predicted to decline. Our results suggest at the forested slough, streamflow above 50

m^3/sec may increase water depths in the wetland dominated by shallow and deep marsh habitats (~~See~~ Kantrud et al. 1989) ~~wetland~~ above preferred levels for boreal chorus frogs.

The forested slough has a more permanent hydroperiod and often year-round water availability with close proximity to the Platte River, creating suitable habitat for *Lithobates catesbeianus* (American bullfrog) and posing a potential predation risk to native species through temporary colonization of fish species (Skelly 1996, Goldowitz and Whiles 1999, Knapp and Matthews 2000). Comparatively, the wet meadow exhibits more intermittent hydrology not conducive to bullfrogs. Bullfrog vocalizations were heard semi-regularly in audio recordings at the forested slough, potentially influencing *P. maculata* activity through resource competition, predation, and disease transmission such as the fungal pathogen *B. dendrobatidis* (Pearl et al. 2004, Harner et al. 2013). Introductions of *L. catesbeianus* to aquatic systems have shown that native species of frogs will change their calling behavior in response to overlapping vocalization activity (Medeiros et al. 2016). At the forested slough, *P. maculata* vocalizations were relatively inactive during the day compared to the wet meadow, where we observed more consistent calling activity within a 24-hour period (Figure 2). Conversely, woodlands are not preferred habitat for *P. maculata*, which may provide further explanation for lower calling activity at the forested slough (Ballinger et al. 2010, Malzahn *In Review*).

~~As *P. maculata* relies on ponded water for critical stages in their life cycle, including breeding, egg laying, and metamorphose, hydropattern is likely a key determinant of activity patterns (Saenz et al. 2006, Ballinger et al. 2010). Prior research shows *P. maculata* is adapted to local hydroperiod and lacks plasticity, suggesting their ability to respond to changes in hydropattern may be limited (Amburgey et al. 2012). In a semi-arid region of the Great Plains, water and land use changes have greatly altered wetlands and their hydropattern within the Central Platte River~~

Formatted: Font: Italic

Formatted: Font: Italic

Formatted: Indent: First line: 0 cm

Valley (Sidle et al. 1989). Most of the wetlands and wet meadows are connected to the hydrology of the Platte River and exhibit fluctuating and seasonal hydrology, with hydroperiods ranging from temporary pools to permanent sloughs (Henszey and Wesche 1993). *P. maculata* occur in wetlands with varying hydroperiods, however, they are predominantly found in wetlands with intermediate hydrology (Kolozsvary and Swihart 1999). In the CPRV, Goldowitz and Whiles (1999) found *P. maculata* was most abundant at intermittent wetlands, where water was present 60-90% of the time. Similarly, Geluso and Harner (2013a) found *P. maculata* did not occupy perennial waters. Our results indicate similar findings, as hydropattern was an important variable at both the wet meadow and forested slough. In mid-April 2016 as well as the end of April and beginning of May 2017, *P.maculata* calling activity decreased precipitously at both sites, concurrent with a decline in wet meadow inundation and decrease in Platte River streamflow. This suggests the decrease in *P. maculata* vocalizations was likely attributed to water availability. At both sites, seven-day rainfall accumulation was an important predictor of *P. maculata* calling activity. This indicates precipitation regimes sufficient to inundate temporary and intermittent wetlands likely act as an environmental cue for opportunistic *P. maculata* breeding activity (Saenz et al. 2006, Ballinger et al. 2010).

Prior research suggests that predation, hydropattern, and interspecific competition may drive the presence, abundance, and distribution of *Pseudacris* species (Skelly 1996, Amburgey et al. 2014). During our study period, the wet meadow remained inundated for most of the *P. maculata* breeding season during most years, with calling activity predicted to increase when water inundation was above 0.2. At the forested slough, *P. maculata* activity was predicted to increase when streamflow reached 50 m^3/sec (~1766 ft^3/sec; Figure 4), and when streamflow exceeded this, *P. maculata* activity was predicted to decline. *P. maculata* prefers intermittent

Formatted: Font: Italic

wetlands, likely as an avoidance of potential desiccation from quick-drying shallow wetlands and to avoid predation from fish and other frog species within more permanent bodies of water (Dodd 2013). Our results suggest at the forested slough streamflow above 50 m^3/sec may increase water depths in the wetland above preferred levels for Boreal Chorus Frogs. This site exhibits a more permanent hydroperiod with close proximity to the Platte River. It is possible that the backwater forested slough near the river becomes temporarily hydrologically connected to the river at high flows, which may promote temporary colonization of the wetland by fish species that could pose a predation risk to *P. maculata* (Skelly 1996, Goldowitz and Whiles 1999, Knapp and Matthews 2000).

The more permanent hydroperiod and often year-round water availability at the forested slough is habitat for *Lithobates catesbeianus* (American Bullfrog), and bullfrog vocalizations were heard semi-regularly in audio recordings. Contrastingly, intermittent hydrology is not conducive to *L. catesbeianus* habitat, and therefore the species was not found within the wet meadow. *L. catesbeianus* is an introduced species in the CPRV and can have negative impacts on native anuran species through resource competition, predation, and disease transmission such as the fungal pathogen *B. dendrobatidis* (Pearl et al. 2004, Harner et al. 2013). Introductions of *L. catesbeianus* to aquatic systems have shown that native species of frogs will change their calling behavior in response to overlapping vocalization activity (Medeiros et al. 2016). The difference in habitat conditions, such as hydropattern and the presence of predators, may have contributed to the difference in overall *P. maculata* calling activity, with less vocalizations identified at the forested slough, as well as differences in 24-hour calling activity between habitats. At the wet meadow, we observed more consistent calling activity within a 24-hour period, where *P. maculata* vocalizations increased moderately between 07:00 and 13:00, but remained relatively

inactive during the day at the forested slough (Figure 2). An implication for management consideration, restored sloughs in the region are often deeper than many of the naturally occurring wetlands that have been lost in the region. Human-created restoration sloughs can provide perennial water sources when deep and/or connected to the river, similar to the forested slough in this study. Inadvertently, these restoration sloughs may allow for colonization of predators such as nonnative bullfrogs as well as fish, which may have negative impacts on native anurans like *P. maculata*. However, another potential reason for lower *P. maculata* calling activity at the forested slough is that woodlands do not provide preferred habitat (Ballinger et al. 2010). For instance, Malzahn (*In Review*) found that Boreal Chorus Frog abundance had a positive association with distance to nearest woodland in the CPRV.

5. Conclusions

Our findings indicate the spring calling activity of *P. maculata* follows a phenological pattern, initiating on average around day of year 70 and peaking from approximately day of year 100 to 120 in south-central Nebraska, USA. Furthermore, vocalization activity is associated with environmental variables and habitat condition, with predicted calling activity responding to temperatures above 5°C, weekly accumulated precipitation, and hydropattern. We detected greater vocalizations and near continuous diel calling activity in an intermittent wet meadow habitat compared to a forested slough with semi-permanent hydrology. These findings emphasize the importance of hydroperiod for the lifecycle of *P. maculata*. The small ephemeral wetlands preferred by *P. maculata* may be vulnerable to changes in land and water-use as cropland expands, water demands increase, and precipitation regimes change (Amburgey et al. 2012). As they rely on prairie-wetland ecotones, *P. maculata* may be a useful bioindicator reflecting

environmental conditions across both grassland and aquatic habitats (Knutson et al. 2000). Autonomous sound recorders and acoustic metrics are particularly useful for assessing anurans, as they are often elusive, nocturnal, small, yet vocal (Bee et al. 2010, Ulloa et al. 2019, Sugai et al. 2019). Concurrently, time-lapse cameras offer an approach to examine visual changes in habitat and landscape (Sonnentag 2012, Brinley Buckley et al. 2017). Many challenges still exist with implementing bioacoustics and multimodal monitoring, including processing time, learning curves, accuracy of classification, validation, loss of data due to passive technology malfunctions, integrating datasets, and handling, manipulating, and storing large amounts of data (Digby et al. 2013, Sugai et al. 2019). However, many of these limitations can be addressed with additional approaches such as manual listening and validation techniques. Additionally, multimodal monitoring can offer advantages for studying changing natural systems, including the ability to control frequency intervals and time duration, allowing for high frequency passive data collection over a long duration of time, the ability to have spatial replicates and collect data in remote or difficult to reach areas, minimal up-front human resources, and less time allocation than manual surveys or trapping. Declines in anuran populations have emphasized the need for increased monitoring to establish species-specific and geographically-relevant baseline references to characterize anuran phenology and activity patterns. More research is necessary to establish sound management and conservation practices, such as the effects of restoration activities that create permanent waterbodies in landscapes historically dominated by intermittent wetlands and anuran response to fire regimes and grazing. Integration of auditory and visual technologies into multimodal monitoring is a promising approach for understand species-specific responses to changing climatic and environmental conditions, altered habitat, and management practices.

6. Acknowledgements

This study was funded in part by the University of Nebraska Collaboration Initiative, the University of Nebraska at Kearney, the National Science Foundation under Grant No. DRL 1323615, and the United States Department of Agriculture under accession number 1016730. We thank Platte Basin Timelapse (PBT) for the use of imagery and The Crane Trust and Audubon's Rowe Sanctuary for land access. We specifically acknowledge Michael Forsberg and Michael Farrell for their contributions to PBT, as well as Simon Tye for assisting with maintaining sound recorders and Carol Bedoya for assistance with sound analysis.

7. Literature Cited

Abrahams, C. and Geary, M., 2020. Combining bioacoustics and occupancy modelling for improved monitoring of rare breeding bird populations. Ecological Indicators, 112, p.106131.

Alexander, J.S., McElroy, B., Huzurbazar, S., Elliott, C., and Murr, M.L. 2020. Deposition potential and flow- response dynamics of emergent sandbars in a braided river. Water Resources Research, 56(1), e2018WR024107.

Amburgey, S., Funk, W.C., Murphy, M. and Muths, E., 2012. Effects of hydroperiod duration on survival, developmental rate, and size at metamorphosis in ~~boreal chorus frog~~boreal chorus frog tadpoles (*Pseudacris maculata*). Herpetologica, 68(4), pp.456-467.

Amburgey, S. 2014. Species account for ~~Boreal chorus frog~~Boreal chorus frog (*Pseudacris maculata*). Colorado Partners in Amphibian and Reptile Conservation. http://www.coparc.org/colorado-amphibians---boreal-chorus-frog.html

Anderson, A.M., Haukos, D.A. and Anderson, J.T., 1999. Habitat use by anurans emerging and breeding in playa wetlands. Wildlife Society Bulletin, pp.759-769.

Formatted: Indent: Left: 0 cm, Hanging: 0.63 cm

Archibald, G. W. and C. D. Meine. 1996. Family Gruidae (Cranes), p. 60-89. In.], del Hoyo, A. Elliott, J. Sargatal (eds.). Handbook of the birds of the world, Vol. 3: Hoatzin to Auks. Lynx Barcelona, Spain.

Bee, M.A., Cook, J.M., Love, E.K., O'Bryan, L.R., Pettitt, B.A., Schrode, K. and Vélez, A., 2010. Assessing acoustic signal variability and the potential for sexual selection and social recognition in ~~boreal chorus frog~~boreal chorus frogs (*Pseudacris maculata*). Ethology, 116(6), pp.564-576.

Bergeson, D.G., Bradley, M. and Holroyd, G.L., 2001. Food items and feeding rates for wild whooping crane colts in Wood Buffalo National Park. North American Crane Workshop Proceedings. 46.

Both, C., Kaefer, I.L., Santos, T.G., and Cechin, S.T. 2008. An austral anuran assemblage in the Neotropics: seasonal occurrence correlated with photoperiod. Journal of Natural History, 42(3-4), pp 205-222.

Bowles, M.L., and Jones, M.D. 2013. Repeated burning of eastern tallgrass prairie increases richness and diversity, stabilizing late successional vegetation. Ecological Applications, 23(2), pp. 464-478.

Breiman, L., 2001. Random forests. Machine learning, 45(1), pp.5-32.

Bridges, A.S., and Dorcas, M.E. 2000. Temporal variation in anuran calling behavior: implications for surveys and monitoring programs. Copeia, 2000(2), pp 587-592.

Brinley Buckley, E.M, Allen, C.R., Forsberg, M., Farrell, M. and Caven, A.J., 2017. Capturing change: the duality of time-lapse imagery to acquire data and depict ecological dynamics. Ecology and Society, 22(3).

~~Brinley Buckley, E.M., Caven, A.J., Gottesman, B.L., Harner, M.J., Pijanowski, B.C. and Forsberg, M.L., 2018. Assessing biological and environmental effects of a total solar eclipse with passive multimodal technologies. Ecological Indicators, 95, pp.353-369.~~

Brodie, S., Allen-Ankins, S., Towsey, M., Roe, P. and Schwarzkopf, L., 2020. Automated species identification of frog choruses in environmental recordings using acoustic indices. Ecological Indicators, 119, p.106852.

Brown, J.K. 1990. Effects of fire on aquatic systems. In Wild trout IV: proceedings of the symposium. US Government Printing Office, Washington, DC, USA, pp. 106-110.'

Browne, C.L., Paszkowski, C.A., Foote, A.L., Moenting, A., and Boss, S.M. 2009. The relationship of amphibian abundance to habitat features across spatial scales in the Boreal Plains. Ecoscience, 16(2), pp. 209-223.

Buxton, R.T., Lendrum, P.E., Crooks, K.R. and Wittemyer, G., 2018. Pairing camera traps and acoustic recorders to monitor the ecological impact of human disturbance. Global Ecology and Conservation, 16, p.e00493.

Formatted: Indent: Left: 0 cm, First line: 0 cm

Caldwell, C.A., Jacobi, G.Z., Anderson, M.C., Parmenter, R.R., McGann, J., Gould, W.R., DuBey, R. and Jacobi, M.D. 2013. Prescribed-fire effects on an aquatic community of a southwest montane grassland system. North American Journal of Fisheries Management, 33(5), pp.1049-1062.

Caven, A.J., Buckley, E.M.B., King, K.C., Wiese, J.D., Baasch, D.M., Wright, G.D., Harner, M.J., Pearse, A.T., Rabbe, M., Varner, D.M. and Krohn, B. 2019a. Temporospatial shifts in Sandhill Crane staging in the Central Platte River Valley in response to climatic variation and habitat change. Monographs of the Western North American Naturalist, 11(1), pp.33-76.

Caven, A. J., Malzahn, J., Koupal, K. D., Buckley, E. M. B., Wiese, J. D., Rasmussen, R., and Steenson, C. 2019b. Adult Whooping Crane (*Grus americana*) consumption of juvenile channel catfish (*Ictalurus punctatus*) during the avian spring migration in the Central Platte River Valley, Nebraska, USA. Monographs of the Western North American Naturalist, 11(1), 14-23.

Caven, A. J., Varner, D. M., and Drahota, J. 2020. Sandhill Crane abundance in Nebraska during spring migration: making sense of multiple data points. Transactions of the Nebraska Academy of Sciences 40, pp 6–18

Center for Conservation Bioacoustics. 2019. Raven Pro: Interactive Sound Analysis Software (Version 1.6.1) [Computer software]. Ithaca, NY: The Cornell Lab of Ornithology. Available from http://ravensoundsoftware.com/.

Clarke, R.F., 1958. Ecological study of reptiles and amphibians in Osage County, Kansas. Emporia State Research Studies 7(1)

Conant, R. and J.T. Collins. 1991. A field guide to reptiles and amphibians: eastern and central North America. Houghton Mifflin Company. Boston, Massachusetts, USA.

Corn, P.S., Muths, E. and Iko, W.M., 2000. A comparison in Colorado of three methods to monitor breeding amphibians. Northwestern Naturalist, pp.22-30.

Corn, P. S., and Muths, E. 2002. Variable breeding phenology affects the exposure of amphibian embryos to ultraviolet radiation. Ecology, 83(11), pp. 2958-2963.

Corn, P.S., 2003. Amphibian breeding and climate change: importance of snow in the mountains. Conservation Biology, 17(2), pp.622-625.

Currier, P. J., and R. J. Henszey. 1996. Platte River Wet Meadows: A Primer on their Flora, Fauna, Hydrology, Management, and Restoration. Technical Report, US Fish and Wildlife Service (Project No. 14- 16- 0006- 90-917), Grand Island, NE.

Cutler, A., Cutler, D.R. and Stevens, J.R., 2012. Random forests. In Ensemble machine learning (pp. 157-175). Springer, Boston, MA.

Davis, C.A., J.E. Austin, and D.A. Buhl. 2006. Factors influencing soil invertebrate communities in riparian grasslands of the Central Platte River floodplain. Wetlands 26(2):438-454.

Degenhardt, W.G., Painter, C.W. and Price, A.H., 2005. Amphibians and reptiles of New Mexico. UNM Press.

Digby A, Towsey M, Bell BD, Teal PD. 2013. A practical comparison of manual and autonomous methods for acoustic monitoring. Methods in Ecology and Evolution 4: 675–683.

Dinets, V., 2016. Predation on amphibians and reptiles by reintroduced Whooping Cranes (*Grus americana*) in Louisiana. The American Midland Naturalist, 175(1), pp.134-137.

Dodd, C.K. 2013. Frogs of the United States and Canada. Johns Hopkins University Press, Baltimore, MD, USA. Vo1. 1 pp 371-384.

Driscoll, D.A. and Roberts, J.D., 1997. Impact of fuel- reduction burning on the frog *Geocrinia lutea* in southwest Western Australia. Australian Journal of Ecology, 22(3), pp.334-339.

Eschner, T.R., R.F. Hadley, and K.D. Crowley. 1983. Hydrologic and geomorphic studies of the Platte River Basin. U.S. Geological Survey Professional Paper 1277-A, United States Government Printing Office, Washington, DC. 297 ppFarina A, James P, Bobryk C, Pieretti N, Lattanzi E, McWilliam J. 2014. Low cost (audio) recording (LCR) for advancing soundscape ecology towards the conservation of sonic complexity and biodiversity in natural and urban landscapes. Urban Ecosystems 17: 923–944.

Fuhlendorf, S. D., Engle, D. M., Kerby, J. A. Y., and Hamilton, R. 2009. Pyric herbivory: rewilding landscapes through the recoupling of fire and grazing. Conservation Biology, 23(3), pp 588-598.

Fogell, D.D., 2010. Field guide to the amphibians and reptiles of Nebraska. University of Nebraska-Lincoln.

Gasc A, Francomano D, Dunning JB, Pijanowski BC. 2017. Future directions for soundscape ecology: The importance of ornithological contributions. Auk 134: 215–228

Geluso, K., Krohn, B.T., Harner, M.J. and Assenmacher, M.J., 2013. Whooping cranes consume plains leopard frogs at migratory stopover sites in Nebraska. Prairie Naturalist, 45, pp.91-93.

Geluso, K. and Harner, M.J., 2013a. Reexamination of Herpetofauna on Mormon Island, Hall County, Nebraska, with Notes on Natural History. Transactions of the Nebraska Academy of Sciences & Affiliated Societies, 33.

Geluso, K., and M.J. Harner. 2013b. Nerodia clarkii clarkii (Gulf Saltmarsh Watersnake): Predation. Herpetological Review 44:156-157.

Goldowitz, B.S. and Whiles, M.R., 1999. Investigations of Fish, Amphibians and Aquatic Invertebrate Species Within the Middle Platte River System. Final Report, Platte Watershed Program Cooperative Agreement X99708101. USEPA region VII, Kansas City, Missouri, USA.

Grafe, T.U., Doebler, S. and Linsenmair, K.E., 2002. Frogs flee from the sound of fire. Proceedings of the Royal Society of London. Series B: Biological Sciences, 269(1495), pp.999-1003.

Grant, E.H.C., Miller, D.A., Schmidt, B.R., Adams, M.J., Amburgey, S.M., Chambert, T., Cruickshank, S.S., Fisher, R.N., Green, D.M., Hossack, B.R. and Johnson, P.T., 2016. Quantitative evidence for the effects of multiple drivers on continental-scale amphibian declines. Scientific reports, 6(1), pp.1-9.

Grant, T.J., Otis, D.L. and Koford, R.R., 2015. Short- term anuran community dynamics in the Missouri River floodplain following an historic flood. Ecosphere, 6(10), pp.1-16.

Hafner, S, and Katz, J. 2018. monitoR: Acoustic template detection in R. R package version 1.0.7. < http://www.uvm.edu/rsenr/vtcfwru/R/?Page=monitoR/monitoR.htm >

Harner, M.J. and K. Geluso. 2011. Response of herpetofauna to grazing and fire in wet, tallgrass prairies along the Platte River. Nebraska State Wildlife Grant Final Report submitted to the Nebraska Game and Parks Commission, Lincoln, Nebraska, USA. 58 p.

Harner, M.J., Merlino, J.N. and Wright, G.D., 2013. Amphibian chytrid fungus in Woodhouse's toads, plains leopard frogs, and American bullfrogs along the Platte River, Nebraska, USA. Herpetological Review, 44(3), pp.459-461.

Henszey, R.J. and Wesche, T.A., 1993. Hydrologic Components Influencing the Condition of Wet Meadows Along the Central Platte River, Nebraska. Rep. Neb. Game Park. Comm.

Hillman, S.S., Withers PC, Drewes RC, Hillyard SD. 2009. Ecological and environmental physiology of amphibians. New York, NY: Oxford University Press.

Horn, J.D., Joeckel, R.M., and Fielding, C.R. 2012. Progressive abandonment and planform changes of the central Platte River in Nebraska, central USA, over historical timeframes. Geomorphology, 139, pp. 372-383.

Howard, R.D., 1980. Mating behavior and mating success in wood frogs, *Rana sylvatica*. Anim Behav 28:705–716

Hyman, O. 2012. Ecology of Chytridiomycosis in ~~Boreal Chorus Frog~~Boreal chorus frogs (*Pseudacris maculata*). Dissertation, Arizona State University, Tempe, AZ, USA.

Johansson, J., Kristensen, N.P., Nilsson, J.Å. and Jonzén, N. 2015. The eco- evolutionary consequences of interspecific phenological asynchrony–a theoretical perspective. Oikos, 124:102-112.

Johnsgard, P. A. 1983. Cranes of the world. Indiana University Press, Bloomington, Indiana

Jones, S.M., Ballinger, R.E. and Nietfeldt, J.W., 1981. Herpetofauna of Mormon Island Preserve, Hall County, Nebraska. The Prairie Naturalist, 13(2), pp.33-41.

Kantrud, H.A., Krapu, G.L. and Swanson, G.A., 1989. Prairie basin wetlands of the Dakotas: a community profile (Vol. 85, No. 7). US Department of the Interior, Fish and Wildlife Service, Research and Development.

Knapp, R.A., and K.R. Matthews. 2000. N~~n~~on-native fish introductions and the decline of the mountain yellowlegged frog from within protected areas. Conservation Biology 14: 428–438.

Kaul, R.B., Sutherland, D., Rolfsmeier, S. 2011. The flora of Nebraska. School of Natural Resources, University of Nebraska-Lincoln, NE, USA.

Klaus, S.P. and Lougheed, S.C., 2013. Changes in breeding phenology of eastern Ontario frogs over four decades. Ecology and evolution, 3(4), pp.835-845.

Knutson, M.G., Sauer, J.R., Olsen, D.A., Mossman, M.J., Hemesath, L.M. and Lannoo, M.J., 2000. Landscape associations of frog and toad species in Iowa and Wisconsin, USA. Journal of the Iowa Academy of Science: JIAS, 107(3-4), pp.134-145.

Kolozsvary, M.B. and Swihart, R.K., 1999. Habitat fragmentation and the distribution of amphibians: patch and landscape correlates in farmland. Canadian Journal of Zoology, 77(8), pp.1288-1299.

Kramer, D. C., 1973. Movements of western chorus frogs *Pseudacris triseriata triseriata* tagged with Co 60. Journal of Herpetology, 7, pp. 231-235.

LaGrange, T.G. 2005. A Guide to Nebraska's Wetlands and their Conservation Needs. Nebraska Game and Parks Commission, Lincoln, USA.

Liaw, A. and Wiener, M., 2002. Classification and regression by randomForest. R news, 2(3), pp.18-22.

Formatted: Font: Italic

Lingle, G.R., G.A. Wingfield, and J.W. Ziewitz. 1991. The migration ecology of Whooping Cranes in Nebraska, USA. Pages 395–401 in J. Harris, editor, Proceedings of the 1987 International Crane Workshop. International Crane Foundation, Baraboo, WI.

Lofts, B. 1974. Reproduction. In: Lofts B (ed) Physiology of the amphibia, vol II. Academic Press, New York, pp 107–218

Machado, R.B., Aguiar, L., and Jones, G. 2017. Do acoustic indices reflect the characteristics of bird communities in the savannas of Central Brazil?. Landscape and Urban Planning, 162,pp 36-43.

Madalozzo, B., Santos, T.G., Santos, M.B., Both, C., and Cechin, S. 2017. Biodiversity assessment: selecting sampling techniques to access anuran diversity in grassland ecosystems. Wildlife Research, 44(1), pp 78-91.

Mannan, R.N., 2008. An assessment of survey methodology, calling activity, and habitat associations of Wood Frogs (*Rana sylvatica*) and ~~Boreal Chorus Frog~~Boreal chorus frogs (*Pseudacris maculata*) in a tundra biome. Doctoral dissertation, Texas Tech University, Lubbock, Texas, USA.

Malzahn, J., A.J. Caven, S. Warren, B. Ostrom, and D. Ferraro. *In Review*. Habitat associations of various herpetofauna in the Central Platte River Valley, Nebraska, with notes on morphometric characteristics. Platte River Whooping Crane Maintenance Trust, Inc., Wood River, NE, USA, pp. 1-40.

Matthews, J.H., Funk, W.C. and Ghalambor, C.K., 2013. Demographic approaches to assessing climate change impact: an application to pond-breeding frogs and shifting hydropatterns. Wildlife conservation in a changing climate, pp.58-85.

Menne, M.J., I. Durre, B. Korzeniewski, S. McNeal, K. Thomas, X. Yin, S. Anthony, R. Ray, R.S. Vose, B.E.Gleason, and T.G. Houston, 2012. Global Historical Climatology Network - Daily (GHCN-Daily), Version 3.26 NOAA National Centers for Environmental Information http://doi.org/10.7289/V5D21VHZ [Accessed 18 April 2019].

Meyer, C.K., and M.R. Whiles. 2008. Macroinvertebrate communities in restored and natural Platte River slough wetlands. Journal of the North American Benthological Society 27:626-639.

Mushet, D.M, Euliss Jr, N.H, and Stockwell, C.A. 2012. Mapping Anuran Habitat Suitability to Estimate Effects of Grassland and Wetland Conservation Programs. Copeia, 2012(2), pp. 321-330.

Muths, E., Scherer, R.D., Amburgey, S.M., Matthews, T., Spencer, A.W. and Corn, P.S., 2016. First estimates of the probability of survival in a small-bodied, high-elevation frog (~~Boreal Chorus Frog~~Boreal chorus frog, *Pseudacris maculata*), or how historical data can be useful. Canadian Journal of Zoology, 94(9), pp.599-606.

Nelson, G.L., and Graves, B.M. 2004. Anuran population monitoring: comparison of the North American Amphibian Monitoring Program's calling index with mark-recapture estimates for *Rana clamitans*. Journal of Herpetology, 38(3), pp. 355-359.

Oseen, K.L. and Wassersug, R.J., 2002. Environmental factors influencing calling in sympatric anurans. Oecologia, 133(4), pp.616-625.

Ospina, O.E., Villanueva-Rivera, L.J., Corrada-Bravo, C. and Aide, T.M., 2013. Variable response of anuran calling activity to daily precipitation and temperature: implications for climate change. Ecosphere, 4(4), pp.1-12.

Ouellet , M., Fortin, C. and Grimard, M.J., 2009. Distribution and habitat use of the ~~Boreal Chorus Frog~~Boreal chorus frog (Pseudacris maculata) at its extreme northeastern range limit. Herpetological Conservation and Biology, 4(2), pp.277-284.

Pearl, C.A., Adams, M.J., Bury, R.B. and McCreary, B., 2004. Asymmetrical effects of introduced bullfrogs (*Rana catesbeiana*) on native ranid frogs in Oregon. Copeia, 2004(1), pp.11-20.

Pearse, A. T., Krapu, G. L., Cox, R. R., and Davis, B. E. 2011. Spring-migration ecology of Northern Pintails in south-central Nebraska. Waterbirds, 34(1), pp 10-18.

Pekel, J.F., Vancutsem, C., Bastin, L., Clerici, M., Vanbogaert, E., Bartholomé, E. and Defourny, P., 2014. A near real-time water surface detection method based on HSV transformation of MODIS multi-spectral time series data. Remote sensing of environment, 140, pp.704-716.

Pijanowski, B.C., Villanueva-Rivera, L.J., Dumyahn, S.L., Farina, A., Krause, B.L., Napoletano, B.M., Gage, S.H. and Pieretti, N., 2011. Soundscape ecology: the science of sound in the landscape. BioScience, 61(3), pp.203-216.

Pilliod, D.S., Bury, R.B., Hyde, E.J., Pearl, C.A. and Corn, P.S., 2003. Fire and amphibians in North America. Forest ecology and management, 178(1-2), pp.163-181.

R Core Team. 2020. R: A language and environment for statistical computing. R Foundation for Statistical Computing, Vienna, Austria. URL https://www.R-project.org/.

Reichert, B., and Lausen, C., Loeb, S., Weller, T., Allen, R., Britzke, E., Hohoff, T., Siemers, J., Burkholder, B., Herzog, C., and Verant, M., 2018, A guide to processing bat acoustic data for the North American Bat Monitoring Program (NABat): U.S. Geological Survey Open-File Report 2018–1068, 33 p., https://doi.org/10.3133/ofr20181068.

Richardson, A.D., Hufkens, K., Milliman, T., Aubrecht, D.M., Chen, M., Gray, J.M., Johnston, M.R., Keenan, T.F., Klosterman, S.T., Kosmala, M. and Melaas, E.K., 2018. Tracking vegetation phenology across diverse North American biomes using PhenoCam imagery. Scientific data, 5, p.180028.

Ryan, T.J. and Winne, C.T., 2001. Effects of hydroperiod on metamorphosis in *Rana sphenocephala*. The American Midland Naturalist, 145(1), pp.46-53.

Saenz, D., Fitzgerald, L.A., Baum, K.A. and Conner, R.N., 2006. Abiotic correlates of anuran calling phenology: the importance of rain, temperature, and season. Herpetological Monographs, 20(1), pp.64-82.

Scherer, R.D., Muths, E. and Noon, B.R., 2012. The importance of local and landscape-scale processes to the occupancy of wetlands by pond-breeding amphibians. Population Ecology, 54(4), pp.487-498.

Schindelin, J., Arganda-Carreras, I., Frise, E., Kaynig, V., Longair, M., Pietzsch, T., Preibisch, S., Rueden, C., Saalfeld, S., Schmid, B. and Tinevez, J.Y., 2012. Fiji: an open-source platform for biological-image analysis. Nature methods, 9(7), pp.676-682.

Schneider, D.C. 2009. Quantitative ecology: measurement, models and scaling. Academic press, Elsevier, London, UK.

Seburn, D.C., Gunson, K. and Schueler, F.W., 2014. Apparent widespread decline of the ~~Boreal Chorus Frog~~Boreal chorus frog (*Pseudacris maculata*) in eastern Ottawa. The Canadian Field-Naturalist, 128(2), pp.151-157.

Sidle, J.G., E.D. Miller, and P.J. Currier. 1989. Changing habitats in the Platte River valley of Nebraska. Prairie Naturalist 21:91–104.

Silcock, W.R., and J.G. Jorgensen. 2020. Whooping Crane (*Grus americana*), Version 1.0. In Birds of Nebraska — Online**.** www.BirdsofNebraska.org

Skelly, D.K. 1996. Pond drying, predators, and the distribution of *Pseudacris* tadpoles. Copeia 1996(3), pp. 599–605.

Smith, N.D. 1971. Transverse bars and braiding in the lower Platte River, Nebraska. Geological Society of America Bulletin, 82(12), pp.3407-3420.

Sodhi, N.S., Bickford, D., Diesmos, A.C., Lee, T.M., Koh, L.P., Brook, B.W., Sekercioglu, C.H. and Bradshaw, C.J., 2008. Measuring the meltdown: drivers of global amphibian extinction and decline. PloS one, 3(2), p.e1636.

Sommers, M.D., Randall, L.A. and Barclay, R.M.R., 2018. Effects of environmental variables on the calling behaviour of Northern Leopard Frogs (*Lithobates pipiens*) in Alberta, Canada. Canadian Journal of Zoology, 96(2), pp.163-169.

Sonnentag, O., Hufkens, K., Teshera-Sterne, C., Young, A.M., Friedl, M., Braswell, B.H., Milliman, T., O'Keefe, J. and Richardson, A.D., 2012. Digital repeat photography for phenological research in forest ecosystems. Agricultural and Forest Meteorology, 152, pp.159-177.

Spencer A.W. 1964. The Relationship of Dispersal and Migration to Gene Flow in the ~~Boreal Chorus Frog~~Boreal chorus frog. Ph.D. dissertation, Colorado State University, Fort Collins, CO, USA.

Steelman, C.K. and Dorcas, M.E., 2010. Anuran calling survey optimization: developing and testing predictive models of anuran calling activity. Journal of Herpetology, 44(1), pp.61-68.

Stevens, C.E., Diamond, A.W., & Gabor, T.S. 2002. Anuran call surveys on small wetlands in Prince Edward Island, Canada restored by dredging of sediments. Wetlands, 22(1), pp. 90-99.

Strobl, C., Boulesteix, A.L., Kneib, T., Augustin, T. and Zeileis, A., 2008. Conditional variable importance for random forests. BMC bioinformatics, 9(1), p.307.

Sueur, J. and Farina, A., 2015. Ecoacoustics: the ecological investigation and interpretation of environmental sound. Biosemiotics, 8(3), pp.493-502.

Sugai, L.S.M., Silva, T.S.F., Ribeiro Jr, J.W. and Llusia, D., 2019. Terrestrial passive acoustic monitoring: review and perspectives. BioScience, 69(1), pp.15-25.

Todd, M.J., Cocklin, R.R., and Dorcas, M.E. 2003. Temporal and spatial variation in anuran calling activity in the western Piedmont of North Carolina. Journal of the North Carolina Academy of Science, 119(3), pp 103-110.

Visser, M.E. and Gienapp, P., 2019. Evolutionary and demographic consequences of phenological mismatches. Nature Ecology & Evolution, 3(6), pp.879-885.

Vrtiska, M. P., and Sullivan, S. 2009. Abundance and distribution of lesser snow and Ross's geese in the Rainwater Basin and central Platte River Valley of Nebraska. Great Plains Research, 147-155.

Weir, L.A. and Mossman, M.J., 2005. North American Amphibian Monitoring Program (NAAMP). In M. J. Lannoo (ed.), Amphibian Declines: Conservation Status of United States Species, pp. 307-313. University of California Press, Berkeley, California, USA

Werner, E.E., Relyea, R.A., Yurewicz, K.L., Skelly, D.K. and Davis, C.J., 2009. Comparative landscape dynamics of two anuran species: climate- driven interaction of local and regional processes. Ecological Monographs, 79(3), pp.503-521.

Wesche, T.A., O.D. Skinner, and R.J. Henszey. 1994. Platte River wetland hydrology study. Final report prepared for U.S. Bureau of Reclamation by Wyoming Water Resources Center, University of Wyoming, Laramie, Wyoming, 165 p.

Westgate, M.J., MacGregor, C., Scheele, B.C., Driscoll, D.A. and Lindenmayer, D.B., 2018. Effects of time since fire on frog occurrence are altered by isolation, vegetation and fire frequency gradients. Diversity and Distributions, 24(1), pp.82-91.

Whiles, M.R., and B.S. Goldowitz. 1998. Biological responses to hydrologic fluctuation in wetland sloughs of the Central Platte River. Proceedings of the Platte River Basin Ecosystem Symposium 9:3-18.

Whiles, M.R., and B.S. Goldowitz. 2005. Macroinvertebrate communities in Central Platte River wetlands: patterns across a hydrologic gradient. Wetlands 25:462-472.

Whitby, M.D., Carter, T.C., Britzke, E.R. and Bergeson, S.M., 2014. Evaluation of mobile acoustic techniques for bat population monitoring. Acta Chiropterologica, 16(1), pp.223-230.

Wilgers, D.J. and Horne, E.A., 2006. Effects of different burn regimes on tallgrass prairie herpetofaunal species diversity and community composition in the Flint Hills, Kansas. Journal of herpetology, 40(1), pp.73-84.

Williams, G. P. 1978. The Case of the Shrinking Channels: The North Platte and Platte Rivers in Nebraska. Circulars of the United States Geological Survey. No. 781

Data in Brief

Click here to download Data in Brief: Datainbrief_ChorusFrogEnvironmental_9.14.20.zip
